# Cytoprotective Polyketides from Sponge-Derived Fungus *Lopadostoma pouzarii*

**DOI:** 10.3390/molecules27217650

**Published:** 2022-11-07

**Authors:** Phan Thi Hoai Trinh, Anton N. Yurchenko, Olga O. Khmel, Trang Vo Thi Dieu, Ngo Thi Duy Ngoc, Elena V. Girich, Alexander S. Menshov, Natalya Y. Kim, Ekaterina A. Chingizova, Tran Thi Thanh Van, Jong Seok Lee, Hyi-Seung Lee, Ekaterina A. Yurchenko

**Affiliations:** 1 Nhatrang Institute of Technology Research and Application, Vietnam Academy of Science and Technology, Nha Trang 650000, Vietnam; 2 G.B. Elyakov Pacific Institute of Bioorganic Chemistry, Far Eastern Branch of Russian Academy of Science, Prospect 100-Letiya Vladivostoka, 159, Vladivostok 690022, Russia; 3 Institute of High Technologies and Advanced Materials, Far Eastern Federal University, 10 Ajax Bay, Russky Island, Vladivostok 690922, Russia; 4 Marine Natural Products Chemistry Laboratory, Korea Institute of Ocean Science and Technology, Busan 49111, Korea

**Keywords:** marine fungi, *Lopadostoma pouzarii*, sea sponge, secondary metabolites, polyketides, cytotoxicity, cardioprotection

## Abstract

The new polyketides lopouzanones A and B, as well as the new 1-*O*-acetyl and 2-*O*-acetyl derivatives of dendrodochol B, were isolated from the sponge-derived marine fungus *Lopadostoma pouzarii* strain 168CLC-57.3. Moreover, six known polyketides, gliorosein, balticolid, dendrodolide G, dihydroisocoumarine, (–)-5-methylmellein, and dendrodochol B, were identified. The structures of the isolated compounds were determined by a combination of NMR and ESIMS techniques. The absolute configurations of the lopouzanones A and B were determined using the Mosher’s method. The cytotoxicity of the isolated compounds against human prostate cancer cells PC-3 and normal rat cardiomyocytes H9c2 was investigated. Gliorosein showed weak DPPH radical-scavenging activity and in vitro cardioprotective effects toward rotenone toxicity and CoCl_2_-mimic hypoxia.

## 1. Introduction

The marine fungi associated with marine invertebrates, especially sponges, along with the fungi of marine sediments and marine plants, are the main producers of new biologically active compounds [1].

From 1998 to 2017, fungi were found to be an important component of sponge-derived microbes—up to 73%, while actinomycetes and bacteria account for 16% and 11% of sponge-derived microbes [2]. Additionally, nearly a fifth of all new compounds from marine fungi were isolated from sponge-associated ones from 2010 to 2020 [1,3,4,5].

The association between sponges and fungi is very interesting for study because the level of relationship between these two groups of organisms is not entirely clear [6]. Perhaps the sponge and its associated fungi are linked by symbiosis, or there is a different type of interaction. Sponges have been studied for a long time as a source of cytotoxic substances [7]. However, several cases are known where the substances isolated from a sponge were then found in the microbials isolated from this sponge. For instance, antimalarial alkaloid manzamine A, isolated from a marine sponge *Acanthostrongylophora* aff. *ingens* [8], was later isolated from its symbiont *Micromonospora* sp. [9]. 1,2-Bis(1H-indol-3-yl) ethane-1,2-dione, an indole alkaloid from the marine sponge *Smenospongia* sp. [10], was later isolated from marine fungus *Penicillium vinaceum*, associated with marine sponge *Hyrtios erectus* [11].

The *Lopadostoma pouzarii* fungus was first isolated from the *Fraxini excelsioris* tree and identified as a new species in the *Lopadostoma* genus (Xylariaceae, Xylariales) [12]. Later two cultures of this fungus were sequenced and found not to belong to *Lopadostoma*, but they perhaps belong to another genus in the family Xylariaceae, probably *Whalleya* [13]. Nonetheless, both the *Lopadostoma* and the *Whalleya* genera have apparently not been studied much in terms of their secondary metabolites [14]. Only antimicrobial azaphilone pigments sassafrins A-D were isolated from another *Lopadostomaceae* fungus *Creosphaeria sassafras* [15]. Other Xylariales fungi are more studied and produced most of the various polyketides [14].

In the continuation of our investigation of the biodiversity and secondary metabolites of Vietnamese marine fungi [16,17], the *Lopadostoma pouzarii* strain 168CLC-57.3 was isolated from an unidentified marine sponge and cultured for low-molecular weight metabolite isolation.

**Figure 1:**
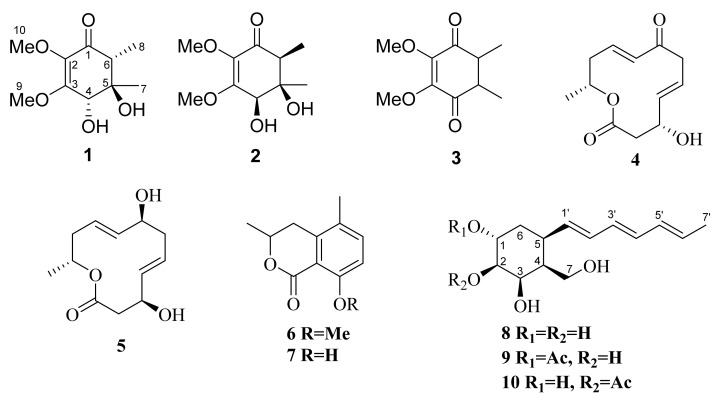
Structures of isolated compounds **1–10**.

Herein, we report the isolation, structure elucidation, and biological activity of ten compounds, including four new ones (Figure 1) from the marine sponge-derived fungus *L. pouzarii*.

## 2. Results

### 2.1. Isolation and Identification of Compounds ***1***–***10***

The molecular formula of compound **1** was determined as C_10_H_16_O_5_, based on the HRESIMS data (*m/z* 239.0890 [M+Na]^+^) corresponding with the ^13^C NMR data. An analysis of ^1^H and ^13^C NMR data (Table 1, Figures S1–S5 from Supplementary Materials) of **1** using DEPT and HSQC techniques revealed the presence of two methoxy-(*δ*_C_ 57.6, *δ*_H_ 3.92; *δ*_C_ 59.3, *δ*_H_ 3.47), two methyl (*δ*_C_ 12.9, *δ*_H_ 1.08; *δ*_C_ 20.6, *δ*_H_ 1.11), and two methine (*δ*_C_ 72.9, *δ*_H_ 4.14; *δ*_C_ 51.4, *δ*_H_ 2.25) groups, as well as a quaternary *sp^3^*-carbon (*δ*_C_ 72.8) and three quaternary *sp^2^*-carbons (*δ*_C_ 196.4; *δ*_C_ 160.6; *δ*_C_ 134.7), chemical shifts of which represented a typical case of α,β-unsaturated ketone. In addition, there were two hydroxyl protons (*δ*_H_ 4.76, *δ*_H_ 5.74) in the structure.

**Figure 2:**
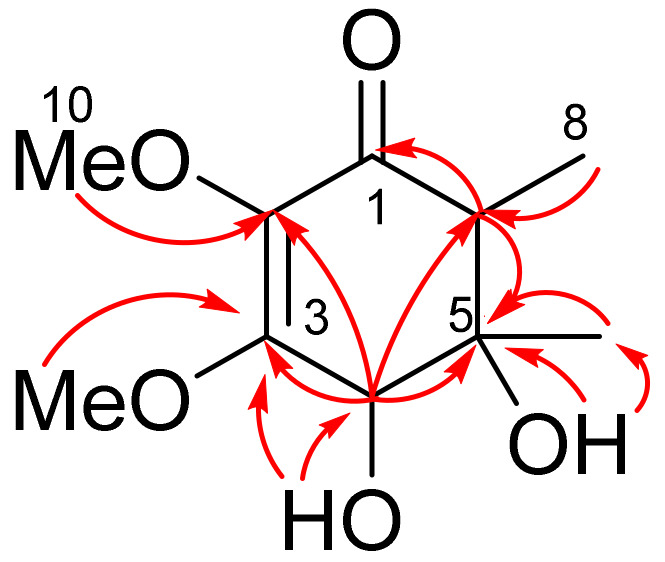
Key HMBC correlations in lopouzanone A (**1**).

The HMBC correlations (Figure 2 and Figure S4 from Supplementary Materials) from H-6 (*δ*_H_ 2.25) to C-1 (*δ*_C_ 196.4) and C-5 (*δ*_C_ 72.8), as well as from H-4 (*δ*_C_ 4.14) to C-2 (*δ*_C_ 134.7), C-3 (*δ*_C_ 160.6), and C-5 showed a structure of a cyclohexenone ring in **1**. The locations of the methyl groups at C-5 (*δ*_C_ 72.8) and C-6 (*δ*_C_ 51.4), the methoxyl groups at C-2 (*δ*_C_ 134.7) and C-3 (*δ*_C_ 160.6), and the hydroxyl groups at C-4 (*δ*_C_ 72.9) and C-5 were determined by HMBC correlations from C_3_-7 (*δ*_H_ 1.11) to C-5, from C_3_-8 (*δ*_H_ 1.08) to C-6, from C_3_-9 (*δ*_H_ 3.92) to C-3, from C_3_-10 (*δ*_H_ 3.47) to C-2, from 4-OH (*δ*_C_ 5.74) to C-3 and C-4, and from 5-OH (*δ*_C_ 4.76) to C-5 and C-7 (*δ*_C_ 20.6).

**Figure 3:**
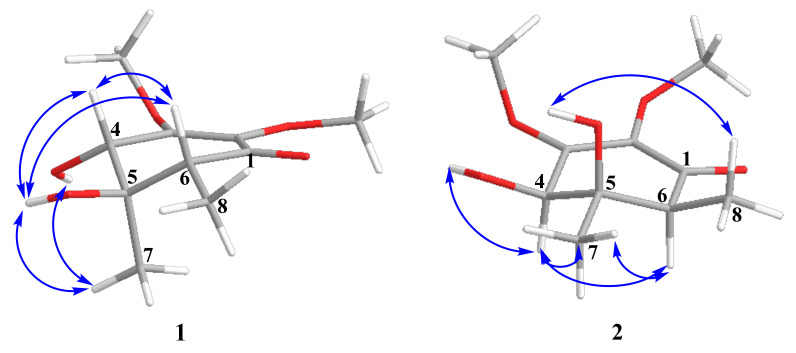
Key ROESY correlations in compounds **1** and **2**.

**Figure 4:**
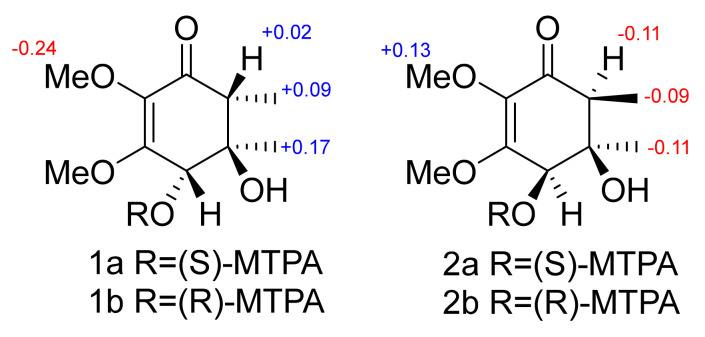
∆δ(δ_S_-δ_R_) values (in ppm) for MTPA esters of **1** and **2**.

The relative configurations of **1** were assigned based on ROESY correlations (Figure 3 and Figure S6 from Supplementary Materials) between H-4 (δ_H_ 4.14), H-6 (δ_H_ 2.20), and 5-OH (δ_H_ 4.76) and between H_3_-7 and 4-OH. The absolute configuration of **1** was established by the modified Mosher’s method [18]. The esterification of **1** with (*S*)- and (*R*)-MTPA chloride occurred at the C-4 hydroxy group to yield the (*R*)-and (*S*)-MTPA esters **1a** and **1b**, respectively. The observed chemical shift differences Δδ(δ*_S_*-δ*_R_*) (Figure 4 and Figures S8–S11 from Supplementary Materials) indicated the 4*S* configuration, and therefore, the absolute configurations of **1** were established as 4*S*,5*R*,6*R*. Compound **1** was named lopouzanone A.

The molecular formula of compound **2** was determined as C_10_H_16_O_5_ (same as **1**), based on the HRESIMS data (*m*/*z* 239.0889 [M+Na]^+^) corresponding with the ^13^C NMR data. An analysis of the NMR data of **2** (Table 1, Figures S12–S16 from Supplementary Materials) and a comparison them with those for lopouzanone A (**1**) showed the chemical shift differences, especially in the chiral part of the molecule. On the other hand, the HMBC correlations of **2** (Figure S15 from Supplementary Materials) were in complete agreement with those for **1**, which proved the same planar structure.

The relative configurations of **2** were assigned based on the ROESY correlations (Figure 3 and Figure S17 from Supplementary Materials) between H-4 (δ_H_ 4.25), H-6 (δ_H_ 2.32), and H_3_-7 (δ_H_ 1.19). The absolute configuration of **2** was established by the modified Mosher’s method [18]. The esterification of **2** with (*S*)- and (*R*)-MTPA chloride occurred at the C-4 hydroxy group to yield the (*R*)-and (*S*)-MTPA esters **2a** and **2b**, respectively. The observed chemical shift differences Δδ(δ*_S_*-δ*_R_*) (Figure 4) indicated the 4*R* configuration, and therefore, the absolute configurations of **2** were established as 4*R*,5*R*,6*S*. Compound **2** was named lopouzanone B.

The molecular formulas of compounds **9** and **10** were determined as C_16_H_24_O_5_ on the basis of the HRESIMS data (*m*/*z* 319.1516 [M+Na]^+^) and confirmed by the ^13^C NMR data. The NMR data of **9** were very close to those for the known dendrodochol B (**8**) [19], with the exception of the proton and carbon signals of the methines at C-1 and C-2 and the methylene at C-6 (Table 2, Figures S34–S40 from Supplementary Materials). These changes, the characteristic signals of the acetate group (*δ*_C_ 172.9, 21.2; δ_H_ 2.05), and the molecular mass difference (42 mass units) between **8** and **9** suggested the acetylation of the hydroxy-group at C-1. This was proved by HMBC (Figure S39 from Supplementary Materials) from H-1 (δ_H_ 3.79) to C-8 (*δ*_C_ 172.9). The NMR spectra of compound **10** (Table 2, Figures S42–S45 from Supplementary Materials) suggested the presence of an acetate group at C-2, which was proved by HMBC from H-2 to C-8. Thus, compounds **9** and **10** were named 1-*O*-acetyl dendrodochol B and 2-*O*-acetyl dendrodochol B, respectively. An analysis of the vicinal constants for compounds **8**, **9,** and **10** revealed the same relative configurations of all stereocenters. Moreover, the absolute stereochemistry of **9** and **10** was assumed to be the same as previously described for dendrodochol B (**8**) (1*R*,2*S*,3*R*,4*S*,5*R*) based on their obvious biosynthetic relationship.

In addition to the new compounds **1**, **2**, **9,** and **10**, six known polyketides were isolated from this fungal strain: gliorosein (**3**) [20], balticolid (**4**) [21], dendrodolide G (**5**) [22], dihydroisocoumarine (**6**) [23], (–)-5-methylmellein (**7**) [24], and dendrodochol B (**8**) [19].

### 2.2. Biological Activity of Isolated Compounds

Compounds **6** and **7** were isolated in an insufficient amount; so, their biological activity was not estimated. The radical-scavenging activity of compounds **1–5**, **8**, and **9** was determined by DPPH assay (Table 3). Gliorosein (**3**) showed a statistically significant DPPH-radical scavenging activity and decreased the amount of DPPH radical by 13.9% at a concentration of 100 µM.

The cytotoxic activity of compounds **1–5**, **8**, and **9** toward human prostate cancer PC-3 cells and the rat normal cardiomyocytes H9c2 line is presented in Table 3. The investigated compounds showed a moderate or weak cytotoxic activity. All the compounds at a concentration of 100 µM showed the same cytotoxicity for the rat normal cardiomyocytes H9c2, and none of them decreased H9c2 cell viability by more than by 42%. At a concentration of 10 µM, all the compounds decreased the H9c2 viability by 3–15%. The compounds **1**, **2**, **5**, **8**, and **9** showed a weak cytotoxic activity toward the human prostate cancer PC-3 cells. They decreased the PC-3 cell viability by only 27–32% at a concentration of 100 µM; so, its concentrations of 50% inhibition were not calculated. The compounds **3** and **4** were more toxic for PC-3 cells, with the IC_50_ of 58.9 µM and 38.9 µM, respectively.

**Figure 5:**
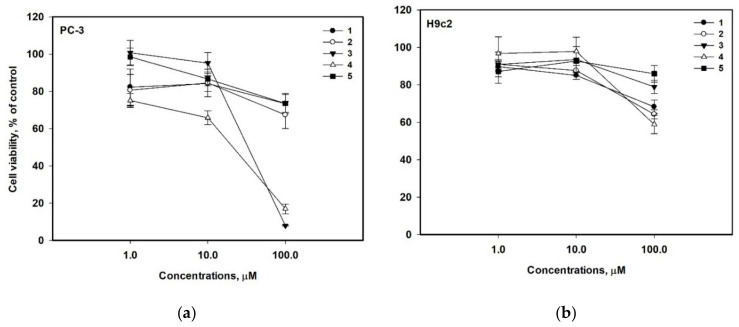
Cytotoxic activity of compounds **1–5** at different concentrations toward PC-3 (**a**) and H9c2 (**b**) cells.

The cytotoxic activity of compounds **1–5** at different concentrations is presented in Figure 5.

**Figure 6:**
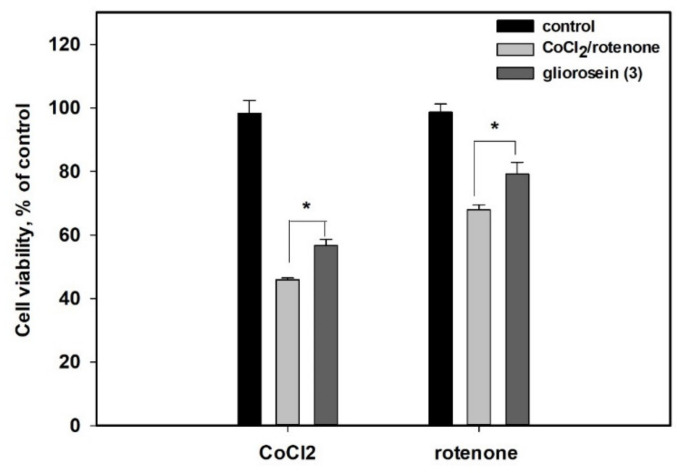
The cytoprotective activity of gliorosein (**3**) at 10 µM against CoCl_2_-mimic hypoxia and rotenone toxicity in H9c2 cells. The data are presented as a mean ± standard error of mean (SEM). * The differences are significant with *p* ≤ 0.05.

The cardioprotective effects of the isolated compounds against rotenone and cobalt chloride (II) were investigated. The influence of compound **3** at a nontoxic concentration of 10 µM on the viability of rotenone- and CoCl_2_-treated cardiomyocytes H9c2 is presented in Figure 6. The treatment of H9c2 cells with CoCl_2_ decreased the viability of H9c2 cells by 54.1%. Compound **3** at 10 µM increased the viability of CoCl_2_-treated H9c2 cells by 23.5%.

The treatment of H9c2 cells with rotenone decreased the viability of H9c2 cells by 32.1%. Compound **3** at a same concentration increased the viability of the rotenone-treated H9c2 cells by 16.6%. Both of these results were statistically significant. Other isolated compounds were not effective in the experiments.

## 3. Discussion

So, ten polyketides were isolated from the *Lopadostoma pouzarii* fungus, which was isolated from the marine environment for the first time. Known gliorosein (**3**) was reported earlier only from terrestrial fungi *Gliocladium* spp. [25].

**Figure 7:**
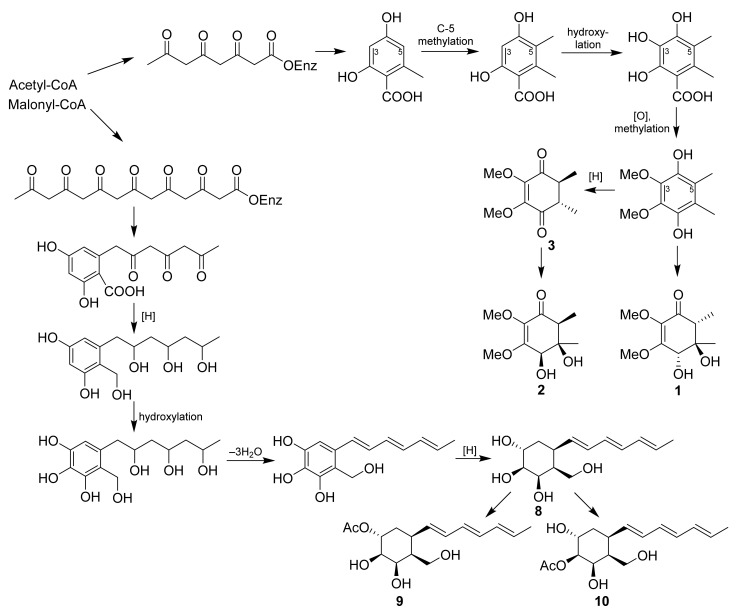
Proposal biosynthetic pathway for compounds **1**–**3** and **8**–**10**.

The biosynthesis of isolated new compounds was suggested (Figure 7). We supposed that the biogenesis of lopouzanones A and B was very probably related with gliorosein (**3**). As was investigated earlier [26], gliorosein originates from tetraketyde 5-methylorsellinic acid. A hydroxylation of the C-5 atom of gliorosein (**3**) gives lopouzanone B (**2**), and the reduction and hydroxylation of the direct precursor of gliorosein gives lopouzanone A (**1**). Dendrodochol B (**8**) and its derivatives **9** and **10** obviously originate from the heptaketyde precursor that cyclizes to acyl orsellinic acid, the main structural features of which are in structures of **8**–**10**. The consistent reduction, hydroxylation, side chain dihydroxylation, and stereoselective reduction of the aromatic ring yield dendrodochol B and its acetylated derivatives.

This is the first report about the selective cytotoxic activity of gliorosein (**3**) and balticolide (**4**) against the human prostate cancer PC-3 cells. Earlier, gliorosein was reported to be an antimicrobial compound [27], and selective cytotoxic activity against the HCT116 cell line was reported for the closely related macrolides balticolide [21] and dendrodolide E [22].

At a nontoxic concentration, gliorosein (**3**) showed cardioprotective activity against the rotenone- and CoCl_2_-induced damage of the cardiomyocytes H9c2.

The treatment of cultured cells with CoCl_2_ is well known and widely used in the in vitro model of hypoxia [28]. Hypoxia causes various pathological processes in the cells, including oxidative stress via the blocking of the mitochondrial respiratory chain, which results in an increase in the reactive oxygen species levels into cells [29]. CoCl_2_ also induces oxidative stress into H9c2 cardiomyocytes via ROS formation [30]. The natural isoflavonoid rotenone used for the in vitro and in vivo modeling of Parkinson’s disease inhibits the mitochondrial complex I, resulting in oxidative stress and cell damage [31]. The inhibition of mitochondrial complex I in H9c2 cardiomyocytes also alters their pro/antioxidant system balance and cell viability [32].

The antioxidant defense against oxidative stress in fungi as well as other eukaryotes is a network of enzymatic superoxide dismutase, catalases, and the thioredoxin system. The fungal response to oxidative stress is therefore complex and necessitates the collaborative involvement of cognate regulators such as the HOG pathway, Yap1, and Skn7 [33]. Nonenzymatic radical-scavenging secondary metabolites have overlapping roles in intercepting ROS and in turning over the cellular buffer of reductants. Polyketides are one of these radical-scavenging compounds. Gliorosein (**3**) showed a weak radical-scavenging activity in a cell-free assay and can protect H9c2 cells from rotenone- and hypoxia-induced death via ROS scavenging. The anti-radical effect of gliorosein (**3**) in the DPPH test was not very high, but it was statistically significant. This effect is difficult to correlate directly with its intracellular action on ROS since the difference in the concentration of free radicals in the cell and in the DPPH test can be significant. Earlier, we observed how other orsellinic acid derivatives showed a weak activity in the DPPH test and were effective in a cellular model of Parkinson’s disease and were also associated with oxidative stress and ROS [34]. Nevertheless, the cardioprotective properties of gliorosein (**3**) in hypoxia conditions need future investigations.

## 4. Materials and Methods

### 4.1. General

Optical rotations were measured on a Perkin-Elmer 343 polarimeter (Perkin Elmer, Waltham, MA, USA). UV spectra were recorded on a Shimadzu UV-1601PC spectrometer (Shimadzu Corporation, Kyoto, Japan) in methanol. CD spectra were measured with a Chirascan-Plus CD spectrometer (Leatherhead, UK) in methanol. NMR spectra were recorded on Bruker DPX-300, Bruker DPX-500, Bruker DPX-600, and Bruker DRX-700 spectrometers (Bruker BioSpin GmbH, Rheinstetten, Germany), using TMS as an internal standard. Low-pressure liquid column chromatography was performed using ODS gel (12 nm, S-75 µM, YMC CO., Kyoto, Japan). HRESIMS spectra were measured on a Maxis Impact mass spectrometer (Bruker Daltonics GmbH, Rheinstetten, Germany).

Low-pressure liquid column chromatography was performed using silica gel (50/100 μm, Imid Ltd., Krasnodar, Russia). Plates precoated with silica gel (5–17 μm, 10 cm × 10 cm, Imid Ltd., Krasnodar, Russia), and 60 RP-18 F_254_S silica gel (20 cm × 20 cm, Merck KGaA, Darmstadt, Germany) was used for thin-layer chromatography. Preparative HPLC was carried out on an HPLC system consisting of a PrimeLine Binary pump (Analytical Scientific Instruments, Inc., El Sobrante, CA, USA) with a RI-101 refractometer (Shoko Scientific Co. Ltd., Yokohama, Japan) and a Shimadzu LC-20 chromatograph (Shimadzu USA Manufacturing, Canby, OR, USA) with a Shimadzu RID-20A refractometer (Shimadzu Corporation, Kyoto, Japan) using the semi-preparative columns YMC-Pack-ODS-A, 250 × 10 mm, 5 µm) (YMC Corporation, Kyoto, Japan) and YMC ODS-AM (YMC Co., Ishikawa, Japan) (5 µm, 10 mm × 250 mm) and YMC SIL (YMC Co., Ishikawa, Japan) (5 µm, 10 mm × 250 mm) columns.

### 4.2. Fungal Material and Fermentation

The fungal strain 168CLC-57.3 was isolated from an unidentified sponge sample collected at Cu Lao Cham Island, Quang Nam, Vietnam. The fungus was identified as *Lopadostoma pouzarii* by gene sequence analysis of the ITS region. According to BLAST analysis, this fungus had the highest similarity to the *L. pouzarii* strain LPO (89.88%, GenBank accession number KC774601). The strain is currently stored in the NITRA Collection of Marine Microorganisms of the Nhatrang Institute of Technology Research and Application, Vietnam, under code 168CLC-57.3 (GenBank accession number MH752441).

The fungus was cultured in 80 × 500 mL Erlenmeyer flasks, each containing rice (20.0 g), yeast extract (20.0 mg), KH_2_PO_4_ (10 mg), and natural seawater from the Nha Trang Bay (40 mL) at 28 °C for three weeks.

### 4.3. Extraction and Isolation

The fungal mycelia and medium were extracted with EtOAc (24.0 L) and then evaporated in vacuo to yield a crude extract (6.0 g). The crude extract was separated on C18-reversed-phase silica gel (ODS) by flash column chromatography using a gradient of MeOH/H_2_O (1:4 to 100% MeOH, each fraction 500 mL) to yield seven fractions. The fraction eluted with 40% MeOH was purified with a semi-preparative reversed-phase HPLC (3.0 mL/min, RI detector, 10% MeOH/H_2_O) to obtain **1** (7.6 mg, *t*_R_ = 18 min), **2** (4.8 mg, *t*_R_ = 32 min), and **3** (3.4 mg, *t*_R_ = 7 min). Compound **4** (3.8 mg, *t*_R_ = 24 min) and **5** (1.8 mg *t*_R_ = 42 min) were purified from the fraction eluted with 60% MeOH through a semi-preparative reversed-phase HPLC (3.0 mL/min, RI detector, 30% MeOH/H_2_O). The fraction eluted with 70% MeOH was separated by a semi-preparative reversed-phase HPLC (3.0 mL/min, RI detector) using an isocratic elution with 35% MeOH in H_2_O to yield **6** (1.3 mg). Compounds **8** (8.2 mg, *t*_R_ = 34 min) and **9** (2.9 mg, *t*_R_ = 42 min) were isolated from the fraction MeOH–H_2_O (80:20) by a semi-preparative reversed-phase HPLC (45% MeOH/H_2_O, 3.0 mL/min). The fraction eluted with 90% MeOH was purified by HPLC on a YMC-Pack-ODS-A column eluting with MeOH–H_2_O (55:45) to obtain **7** (12.0 mg, *t*_R_ = 26 min) and **10** (4.1 mg, *t*_R_ = 43 min).

### 4.4. Isolated Compounds

Lopouzanone A (**1**): amorphous solid; [α]_D_^20^ − 59.3 (c 0.03, MeOH); CD (c 0.51, MeOH), λ_max_ (∆ε) 207 (3.24), 266 (−2.32), 316 (–1.05) nm, (Figure S7 from Supplementary Materials); UV (MeOH) λ_max_ (log ε) 298 (3.88), 198 (4.17), 238 (3.44) nm; ^1^H and ^13^C NMR data, see Table 1, (Figures S1–S6 from Supplementary Materials); HRESIMS *m*/*z* 239.0890 [M + Na]^+^ (calcd. for C_10_H_16_O_5_Na, 239.0890, Δ 0 ppm).

Lopouzanone B (**2**): amorphous solid; [α]_D_^20^ +164.95 (c 0.097, MeOH); CD (c 0.83, MeOH), λ_max_ (∆ε) 203 (−3.75), 262 (−2.20), 306 (3.23) nm, (Figure S18 from Supplementary Materials); UV (MeOH) λ_max_ (log ε) 266 (4.06), 195 (3.71), 226 (3.25) nm; ^1^H and ^13^C NMR data, see Table 1, (Figures S12–S17 from Supplementary Materials); HRESIMS *m*/*z* 239.0889 [M + Na]^+^ (calcd. for C_10_H_16_O_5_Na, 239.0890, Δ 0.42 ppm).

1-*O*-acetyl dendrodochol B (**9**): amorphous solid; [α]_D_^20^ +130.0 (c 0.01, MeOH); CD (c 0.34, MeOH), λ_max_ (∆ε) 212 (−1.16), 219 (−1.26), 255 (−0.30), 283 (−0.25) nm, (Figure S41 from Supplementary Materials); UV (MeOH) λ_max_ (log ε) 270 (3.52), 234 (3.99), 198 (3.75), 260 (3.50), 207 (3.72) nm; ^1^H and ^13^C NMR data, see Table 2, (Figures S36–S40 from Supplementary Materials); HRESIMS *m*/*z* 319.1516 [M + Na]^+^ (calcd. for C_16_H_24_O_5_Na, 319.1516, Δ 0.0 ppm).

2-*O*-acetyl dendrodochol B (**10**): amorphous solid; [α]_D_^20^ +120.1 (c 0.01, MeOH); ^1^H and ^13^C NMR data, see Table 2, (Figures S42–S45 from Supplementary Materials); HRESIMS *m*/*z* 319.1515 [M + Na]^+^ (calcd. for C_16_H_24_O_5_Na, 319.1516, Δ 0.31 ppm).

### 4.5. DPPH Scavenging Assay

The compounds were dissolved in DMSO, and the solutions (120 µL) were dispensed into wells of a 96-well microplate. In all, 30 µL of the DPPH (Sigma-Aldrich, Steinheim, Germany) solution in MeOH (1.5 × 10^−4^ M) was added to each well. The concentrations of the compounds in mixture were 10 and 100 µM. Pure DMSO was used as a control. The ascorbic acid was used as a positive control. The mixture was shaken and left to stand for 30 min, and the absorbance of the resulting solution was measured at 520 nm with a microplate reader MultiscanFC (ThermoLabsystems Inc., Beverly, MA, USA). The radical scavenging activity of all compounds at 100 µM was presented as a percent of the DMSO data.

### 4.6. Bioassays

#### 4.6.1. Cell Culture

The human cancer PC-3 cells were purchased from ATCC (Manassas, VA, USA). The rat cardiomyocytes H9c2 cells were kindly provided by Prof. Dr. Gunhild von Amsberg from Martini-Klinik Prostate Cancer Center, University Hospital Hamburg-Eppendorf, Hamburg, Germany.

#### 4.6.2. Cell Viability Assay

The PC-3 and H9c2 cells were seeded at concentrations of 5 × 10^3^ cell/well and 3 × 10^3^ cell/well, respectively, and the experiments were started after 24 h. The compounds at concentrations up to 100 µM were added into the wells for 48 h, and the viability of the cells was measured by an MTT (3-(4,5-dimethylthiazol-2-yl)-2,5-diphenyltetrazolium bromide) assay, which was performed according to the manufacturer’s instructions (Sigma-Aldrich, Munich, Germany). All compounds were diisolved with DMSO so that the final concentration of DMSO in the cell culture was not more than 1%. Moreover, DMSO was used as a control.

The results were presented as a percent of the control data, and the concentration of the cell viability inhibition on 50% (IC_50_) was calculated.

#### 4.6.3. Cardioprotective Activity of Compounds in CoCl_2_-Mimic Hypoxia

The H9c2 cells were treated with a dH_2_O-solution of CoCl_2_ at a concentration of 500 µM for 2 h. Then, compounds at a concentration of 10 µM were added for 46 h. The viability of the H9c2 cells was measured by an MTT assay.

#### 4.6.4. Cardioprotective Activity of Compounds against Rotenone-Induced Toxicity

The H9c2 cells were treated with rotenone (0.1% DMSO solution) at a concentration of 10 µM for 1 h. Then, compounds at a concentration of 10 µM were added for 23 h. The viability of the H9c2 cells was measured by an MTT assay.

### 4.7. Statistical Data Evaluation

All the data were obtained in three independent replicates, and the calculated values were expressed as mean ± standard error mean (SEM). A Student’s *t*-test was performed using SigmaPlot 14.0 (Systat Software Inc., San Jose, CA, USA) to determine the statistical significance. The differences were considered statistically significant at *p <* 0.05.

## 5. Conclusions

Thus, the new polyketides lopouzanones A and B, as well as the new 1-*O*-acetyl and 2-*O*-acetyl derivatives of dendrodochol B, were isolated from the sponge-derived marine fungus *Lopadostoma pouzarii* strain 168CLC-57.3. Moreover, six known polyketides, gliorosein, balticolid, dendrodolide G, dihydroisocoumarine, (–)-5-methylmellein, and dendrodochol B, were identified. The isolated compounds showed a weak cytotoxicity against human prostate cancer cells PC-3 and normal rat cardiomyocytes H9c2. Moreover, gliorosein showed in vitro cardioprotective effects toward rotenone toxicity and CoCl_2_-mimic hypoxia.

## Figures and Tables

**Table 1:** NMR data (700 MHz, DMSO-d_6_, *δ* in ppm) for compounds **1** and **2**.

Position	1		2	
	δ_C_, mult	δ_H_ (*J* in Hz)	δ_C_, mult	δ_H_ (*J* in Hz)
1	196.4, C		194.9, C	
2	134.7, C		135.8, C	
3	160.6, C		159.5, C	
4	72.9, CH	4.14, d (6.4)	71.9, CH	4.25, d (7.4)
5	72.8, C		73.7, C	
6	51.4, CH	2.20, q (7.4)	49.1, CH	2.30, q (6.9)
7	20.6, CH_3_	1.11, s	24.1, CH_3_	1.19, s
8	12.9, CH_3_	1.08, d (7.4)	8.6, CH_3_	1.05, d (6.9)
9	57.6, CH_3_	3.92, brs	58.4, CH_3_	3.93, s
10	59.3, CH_3_	3.47, brs	59.4, CH_3_	3.48, s
4-OH		5.74, d (6.4)		5.32, d (7.4)
5-OH		4.76, brs		4.26, s

**Table 2:** NMR data (600 MHz, methanol-d_4_, *δ* in ppm) for compounds **8–10**.

Position	8		9		10	
	δ_C_, mult	δ_H_ (J in Hz)	δ_C_, mult	δ_H_ (J in Hz)	δ_C_, mult	δ_H_ (J в Гц)
1	70.3, CH	3.79, ddd (12.5, 11.2, 4.7)	74.1, CH	5.05, ddd (12.0, 10.0, 4.9)	67.6, CH	4.02, ddd (11.7, 10.0, 4.9)
2	78.4, CH	3.24, dd (9.4; 2.8)	75.5, CH	3.49, dd (9.8, 2.8)	81.1, CH	4.53, dd (9.9, 2.7)
3	71.7, CH	4.15, t (2.4)	71.6, CH	4.20, t (2.8)	68.9, CH	4.27, t (2.7)
4	48.3, CH	1.42, m	48.1, CH	1.45, m	48.0, CH	1.49, m
5	37.5, CH	2.36, tdd (12.5, 9.4, 4.0)	37.2, CH	2.40, tdd (12.5, 9.0, 4.2)	37.2, CH	2.36, tdd (12.5, 9.4, 4.0)
6	40.9, CH_2_	1.83, ddd (12.5, 4.7, 4.0) 1.25, q (12.5)	37.6, CH_2_	a: 1.89, dt (12.5, 4.5) b: 1.29, q (12.5)	40.9, CH_2_	a: 1.91, ddd (12.5, 4.9, 4.0) b: 1.33, q (12.5)
7	62.4, CH_2_	3.57, d (6.5)	62.2, CH_2_	3.59, m	62.0, CH_2_	3.55, m
8			172.9, C		172.8, C	
9			21.2, CH_3_	2.05, s	21.1, CH_3_	2.12, s
1′	137.0, CH	5.43, dd (14.2, 9.0)	136.2, CH	5.42, dd (14.1, 9.0)	136.4, CH	5.44, dd (14.1, 9.0)
2′	131.3, CH	6.03–6.13, m	131.1, CH	6.05–6.13, m	131.1, CH	6.05–6.13, m
3′	133.1, CH	6.03–6.13, m	133.1, CH	6.05–6.13, m	133.1, CH	6.05–6.13, m
4′	132.9, CH	6.03–6.13, m	133.0, CH	6.05–6.13, m	133.0, CH	6.05–6.13, m
5′	132.3, CH	6.03–6.13, m	132.7, CH	6.05–6.13, m	132.5, CH	6.05–6.13, m
6′	129.9, CH	5.68, dq (14.2, 6.9)	130.0, CH	5.69, dq (14.2, 6.9)	130.0, CH	5.68, dq (14.1, 6.9)
7′	18.3, CH_3_	1.75, d (6.9)	18.3, CH_3_	1.75, d (6.9)	18.3, CH_3_	1.75, d (6.8)

**Table 3:** DPPH radical scavenging and cytotoxic activities of compounds **1–5**, **8**, and **9**.

Compound	DPPH Radicals,	IC_50_, µM	
	% of Control ^1^	PC-3	H9c2
**1**	93.8 ± 0.5	>100	>100
**2**	100.2 ± 1.0	>100	>100
**3**	86.1 ± 1.8 *	58.9 ± 1.5	>100
**4**	98.6 ± 3.4	38.9 ± 1.9	>100
**5**	98.9 ± 1.4	>100	>100
**8**	99.4 ± 3.3	>100	>100
**9**	102.7 ± 1.8	>100	>100
Ascorbic acid	10.5 ± 3.2		

* The differences between this and the control are significant with *p* ≤ 0.05. ^1^ The effect of compounds at a concentration of 100 µM.

## Data Availability

Not applicable.

## Supplementary Materials

The following supporting information can be downloaded at: https://www.mdpi.com/article/10.3390/molecules27217650/s1, Figure S7, Figure S18, Figure S25 and Figure S41: ECD spectra of compounds **1**–**3** and **9**, Figures S1–S6, S12–S17, S23, S24, S26–S40, S42–S45: NMR spectra of compounds **1**–**10**, Figures S8–S11, S19–S22: NMR spectra of MTPA-esters of compounds **1** and **2**, Figures S46–S49: HRESIMS spectra of compounds **1**, **2**, and **8**–**10**.

## Sample Availability

Samples of the compounds are not available.
