# Supplementary material for: Cytoprotective Polyketides from Sponge-Derived Fungus Lopadostoma pouzarii
Source: Molecules. 2022 Nov 7;27(21):7650. doi: 10.3390/molecules27217650 (PMC9655818; doi:10.3390/molecules27217650)

Supplementary Materials

Cytoprotective polyketides from sponge-derived fungus *Lopadostoma pouzarii*

Phan Thi Hoai Trinh[1,#], Anton N. Yurchenko[2,#,*], Olga O. Khmel[3], Trang Vo Thi Dieu[1], Ngo Thi Duy Ngoc[1], Elena V. Girich[2], Alexander S. Menshov[2], Natalya Y. Kim[2], Ekaterina A. Chingizova [2], Tran Thi Thanh Van[1], Jong Seok Lee[4], Hyi-Seung Lee[4] and Ekaterina A. Yurchenko [2,*]

[1] Nhatrang Institute of Technology Research and Application, Vietnam Academy of Science and Technology, Nha Trang 650000, Vietnam

[2] G.B. Elyakov Pacific Institute of Bioorganic Chemistry Far Eastern Branch of Russian Academy of Science, prospect 100-letiya Vladivostoka, 159, Vladivostok 690022, Russia

[3] Institute of High Technologies and Advanced Materials, Far Eastern Federal University, 10 Ajax Bay, Russky Island, Vladivostok 690922, Russia

[4] Korea Institute of Ocean Science and Technology, 385 Haeyang-ro, Yeongdo-gu, Busan 49111, Korea

*** Correspondence:** eyurch@piboc.dvo.ru (E.A.Y.); yurchenkoan@piboc.dvo.ru (A.N.Y.)

# These authors contributed equally to this work

Abstract: The new polyketides lopouzanones A and B as well as new 1-*O*-acetyl and 2-*O*-acetyl derivatives of dendrodochol B were isolated from sponge-derived marine fungus *Lopadostoma pouzarii* strain 168CLC-57.3. Moreover, six known polyketides gliorosein, balticolid, dendrodolide G , dihydroisocumarine, (–)-5-methylmellein and dendrodochol B were identified. Structures of isolated compounds were determined by a combination of NMR and ESIMS techniques. The absolute configurations of lopouzanones A and B were determined using the Mosher's method. The cytotoxicity of isolated compounds against human prostate cancer cells PC-3 and normal rat cardiomyocytes H9c2 were investigated. Gliorosein showed a weak DPPH radical scavenging activity and *in vitro* cardioprotective effects toward rotenone toxicity and $CoCl_2$-mimic hypoxia.

Keywords: marine fungi; sea sponge; secondary metabolites; polyketides; cytotoxicity; cardioprotection.

Figure S1. ^{1}H NMR spectrum of lopouzanone A (1) ... 4
Figure S2. ^{13}C NMR spectrum of lopouzanone A (1) ... 5
Figure S3. ^{1}H-^{1}H COSY spectrum of lopouzanone A (1) ... 6
Figure S4. HMBC spectrum of lopouzanone A (1) ... 7
Figure S5. HSQC spectrum of lopouzanone A (1) ... 8
Figure S6. ROESY spectrum of lopouzanone A (1) ... 9
Figure S7. CD spectrum of lopouzanone A (1) ... 10
Figure S8. ^{1}H NMR spectrum for (R)-MTPA esters of lopouzanone A (1) ... 11
Figure S9. ^{1}H-^{1}H COSY spectrum for (R)-MTPA esters of lopouzanone A (1) ... 12
Figure S10. ^{1}H NMR spectrum for (S)-MTPA esters of lopouzanone A (1) ... 13
Figure S11. COSY spectrum for (S)-MTPA esters of lopouzanone A (1) ... 14
Figure S12. ^{1}H NMR spectrum of lopouzanone B (2) ... 15
Figure S13. ^{13}C NMR spectrum of lopouzanone B (2) ... 16
Figure S14. ^{1}H-^{1}H COSY spectrum of lopouzanone B (2) ... 17
Figure S15. HMBC spectrum of lopouzanone B (2) ... 18
Figure S16. HSQC spectrum of lopouzanone B (2) ... 19
Figure S17. ROESY spectrum of lopouzanone B (2) ... 20
Figure S18. CD spectrum of lopouzanone B (2) ... 21
Figure S19. ^{1}H NMR spectrum for (R)-MTPA esters of lopouzanone B (2) ... 22
Figure S20. ^{1}H-^{1}H COSY spectrum for (R)-MTPA esters of lopouzanone B (2) ... 23
Figure S21. ^{1}H NMR spectrum for (S)-MTPA esters of lopouzanone B (2) ... 24
Figure S22. ^{1}H-^{1}H COSY spectrum for (S)-MTPA esters of lopouzanone B (2) ... 25
Figure S23. ^{1}H NMR spectrum of gliorosein (3) ... 26
Figure S24. ^{13}C NMR spectrum of gliorosein (3) ... 27
Figure S25. CD spectrum of gliorosein (3) ... 28
Figure S26. ^{1}H NMR spectrum of balticolid (4) ... 29
Figure S27. ^{13}C NMR spectrum of balticolid (4) ... 30
Figure S28. ^{1}H NMR spectrum of dendrodolide G (5) ... 31
Figure S29. ^{13}C NMR spectrum of dendrodolide G (5) ... 32
Figure S30. ^{1}H NMR spectrum of dihydroisocoumarine (6) ... 33
Figure S31. ^{13}C NMR spectrum of dihydroisocoumarine (6) ... 34
Figure S32. ^{1}H NMR spectrum of (–)-5-methylmellein (7) ... 35
Figure S33. ^{13}C NMR spectrum of (–)-5-methylmellein (7) ... 36

Figure S34. ^{1}H NMR spectrum of dendrodochol B (8) 37
Figure S35. ^{13}C NMR spectrum of dendrodochol B (8) 38
Figure S36. ^{1}H NMR spectrum of 1-O-acetyldendrodochol B (9) 39
Figure S37. ^{13}C NMR spectrum of 1-O-acetyldendrodochol B (9) 46
Figure S38. ^{1}H-^{1} spectrum of 1-O-acetyldendrodochol B (9) 47
Figure S39. HMBC spectrum of 1-O-acetyldendrodochol B (9) 48
Figure S40. HSQC spectrum of 1-O-acetyldendrodochol B (9) 49
Figure S41. CD spectrum of 1-O-acetyldendrodochol B (9) 50
Figure S42. ^{1}H NMR spectrum of 2-O-acetyldendrodochol B (10) 51
Figure S43. ^{13}C NMR spectrum of 2-O-acetyldendrodochol B (10) 58
Figure S44. HMBC spectrum of 2-O-acetyldendrodochol B (10) 59
Figure S45. HSQC spectrum of 2-O-acetyldendrodochol B (10) 60
Figure S46. HR (+)ESI MS spectrum of lopouzanone A (1) 61
Figure S47. HR (+)ESI MS spectrum of lopouzanone B (2) 61
Figure S48. HR (+)ESI MS spectrum of dendrodochol B (8) 62
Figure S49. HR (+)ESI MS spectrum of 1-O-acetyldendrodochol B (9) 62
Figure S50. HR (+)ESI MS spectrum of 2-O-acetyldendrodochol B (10) 62

Figure S1. ^{1}H NMR spectrum of lopouzanone A (1)

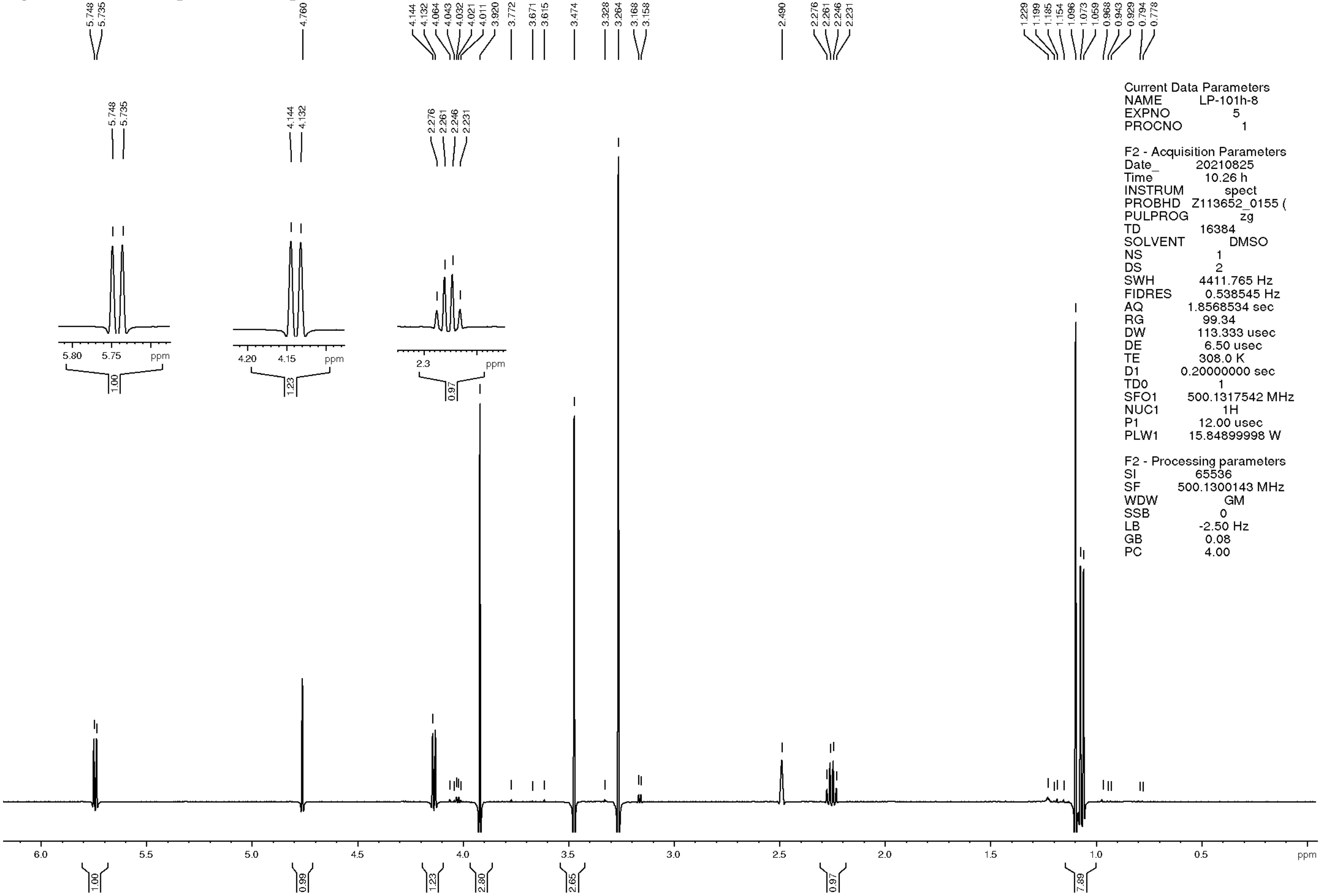

Figure S2. ^{13}C NMR spectrum of lopouzanone A (1)

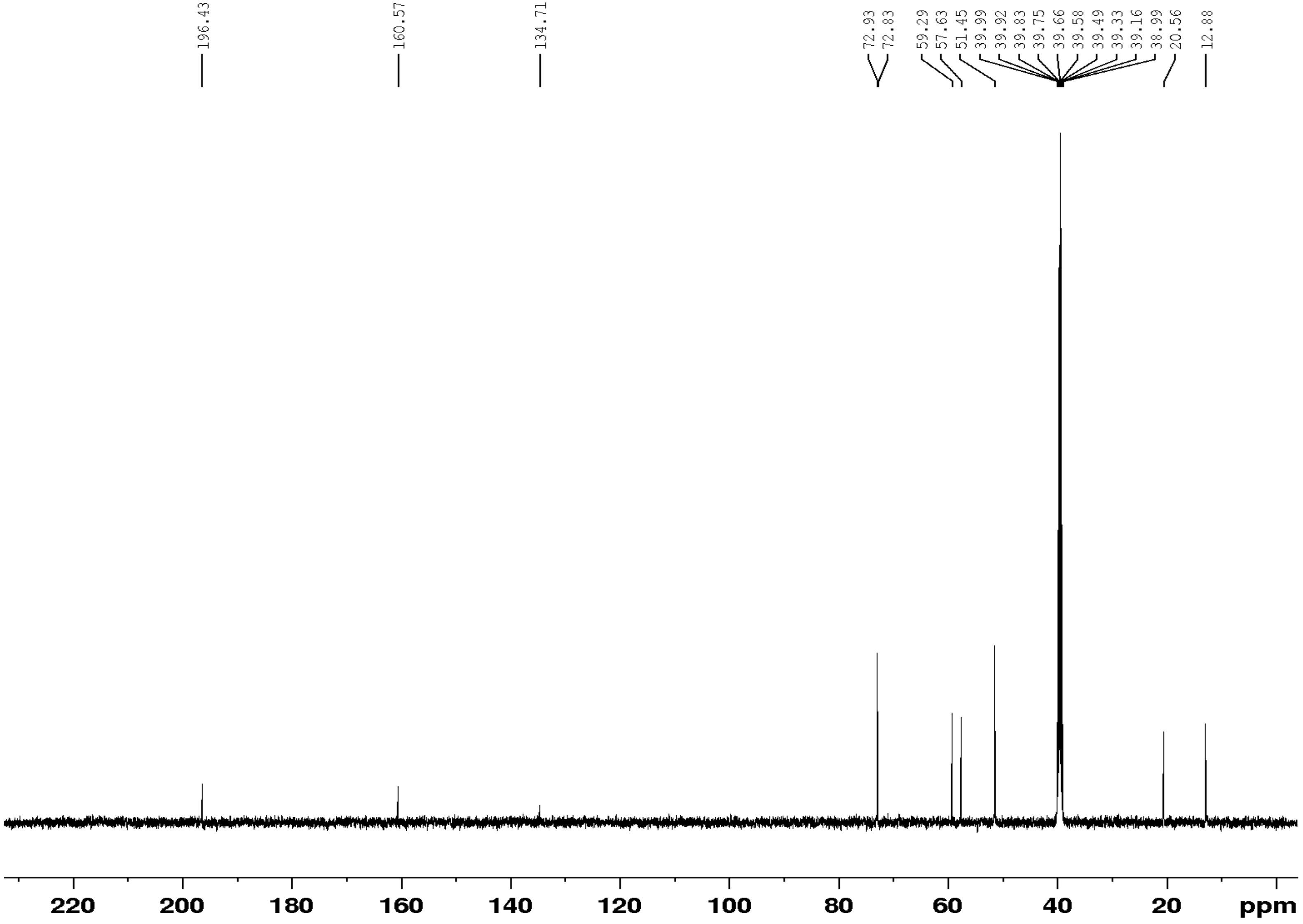


```
Current Data Parameters
NAME            LP-101h-8
EXPNO                  11
PROCNO                  1

F2 - Acquisition Parameters
Date_            20210825
Time                10.26 h
INSTRUM             spect
PROBHD    Z113652_0155 (
PULPROG              zgpg
TD                  32768
SOLVENT              DMSO
NS                    331
DS                      4
SWH             29761.904 Hz
FIDRES           1.816522 Hz
AQ              0.5505024 sec
RG                 196.84
DW                 16.800 usec
DE                   6.50 usec
TE                  308.1 K
D1             2.00000000 sec
D11            0.03000000 sec
TD0                  4096
SFO1          125.7722512 MHz
NUC1                  13C
P1                  11.88 usec
PLW1          79.43299866 W
SFO2          500.1320005 MHz
NUC2                   1H
CPDPRG[2          waltz16
PCPD2               80.00 usec
PLW2          15.84899998 W
PLW12          0.35659999 W
PLW13          0.17937000 W

F2 - Processing parameters
SI                  65536
SF            125.7578629 MHz
WDW                    EM
SSB                     0
LB                   2.00 Hz
GB                      0
PC                   1.30
```

Figure S3. ^1H-^1H COSY spectrum of lopouzanone A (1)

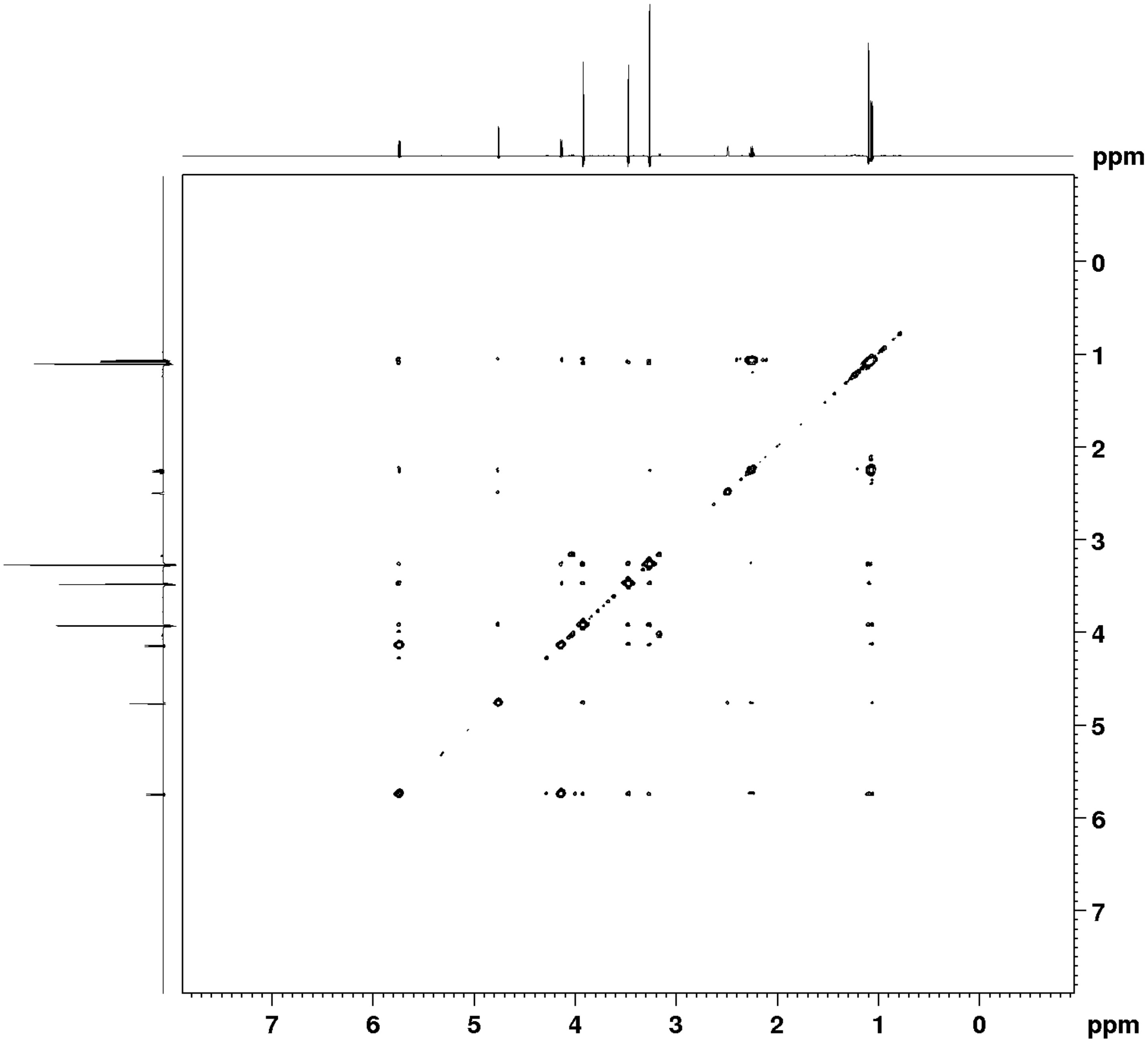


```
Current Data Parameters
NAME              LP-101h-8
EXPNO                    21
PROCNO                    1

F2 - Acquisition Parameters
Date_              20210825
Time                  10.40 h
INSTRUM               spect
PROBHD     Z113652_0155 (
PULPROG           cosygpqf
TD                     2048
SOLVENT                DMSO
NS                        4
DS                       16
SWH                4411.765 Hz
FIDRES             4.308364 Hz
AQ                0.2321067 sec
RG                    99.34
DW                  113.333 usec
DE                     6.50 usec
TE                    308.2 K
D0               0.00000300 sec
D1               1.20000005 sec
D13              0.00000400 sec
D16              0.00020000 sec
IN0              0.00022660 sec
SPARSELI                  0
TDav                      1
SFO1            500.1317542 MHz
NUC1                     1H
P0                     6.00 usec
P1                    12.00 usec
PLW1            15.84899998 W
GPNAM[1]         SMSQ10.100
GPZ1                  10.00 %
P16                 1000.00 usec

F1 - Acquisition parameters
TD                      128
SFO1               500.1318 MHz
FIDRES            68.954102 Hz
SW                    8.824 ppm
FnMODE                   QF

F2 - Processing parameters
SI                     2048
SF              500.1300143 MHz
WDW                   QSINE
SSB                       0
LB                        0 Hz
GB                        0
PC                     1.00

F1 - Processing parameters
SI                     2048
MC2                      QF
SF              500.1300143 MHz
WDW                   QSINE
SSB                       0
LB                        0 Hz
GB                        0
```

Figure S4. HMBC spectrum of lopouzanone A (1)

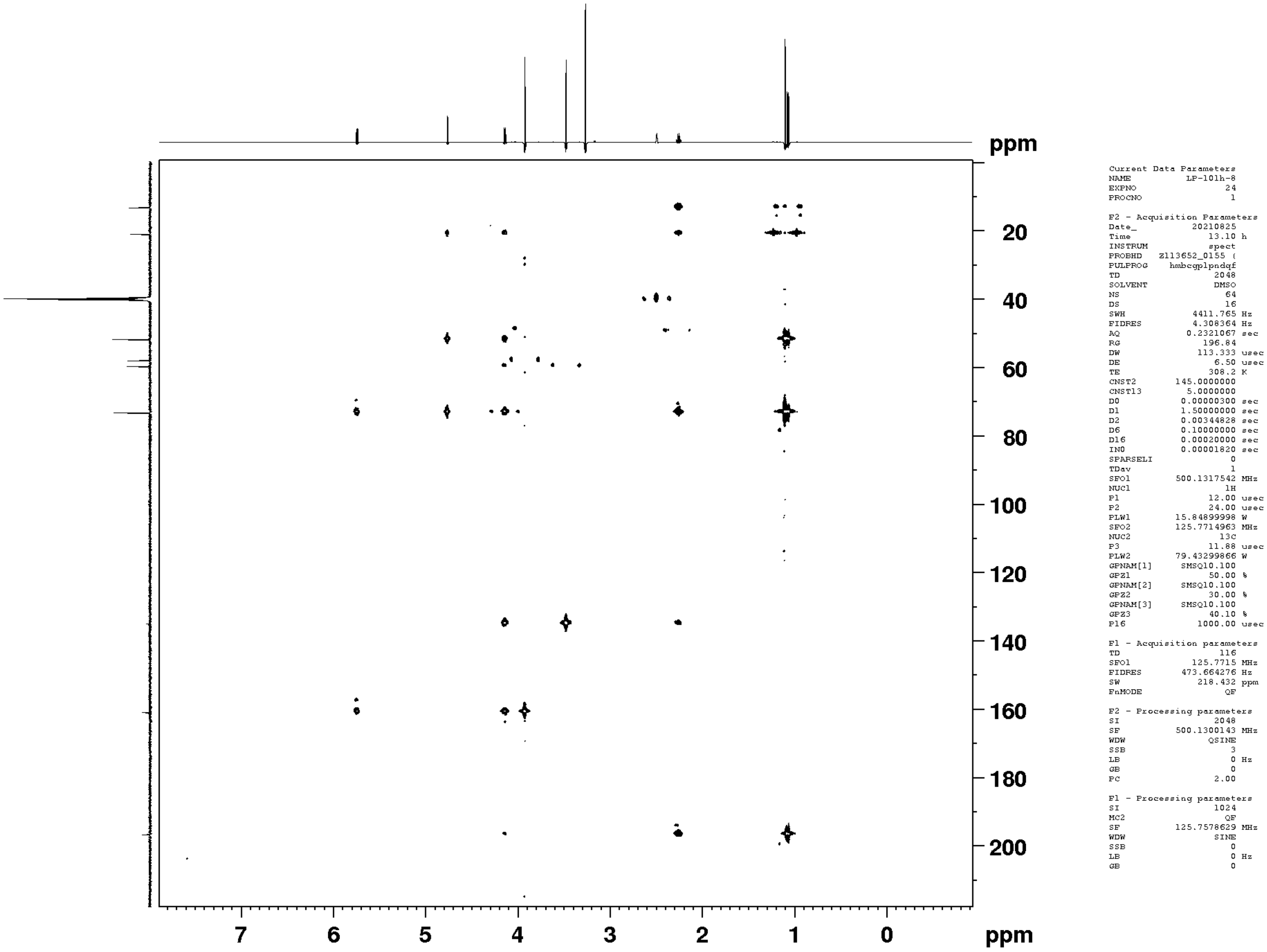

Figure S5. HSQC spectrum of lopouzanone A (1)

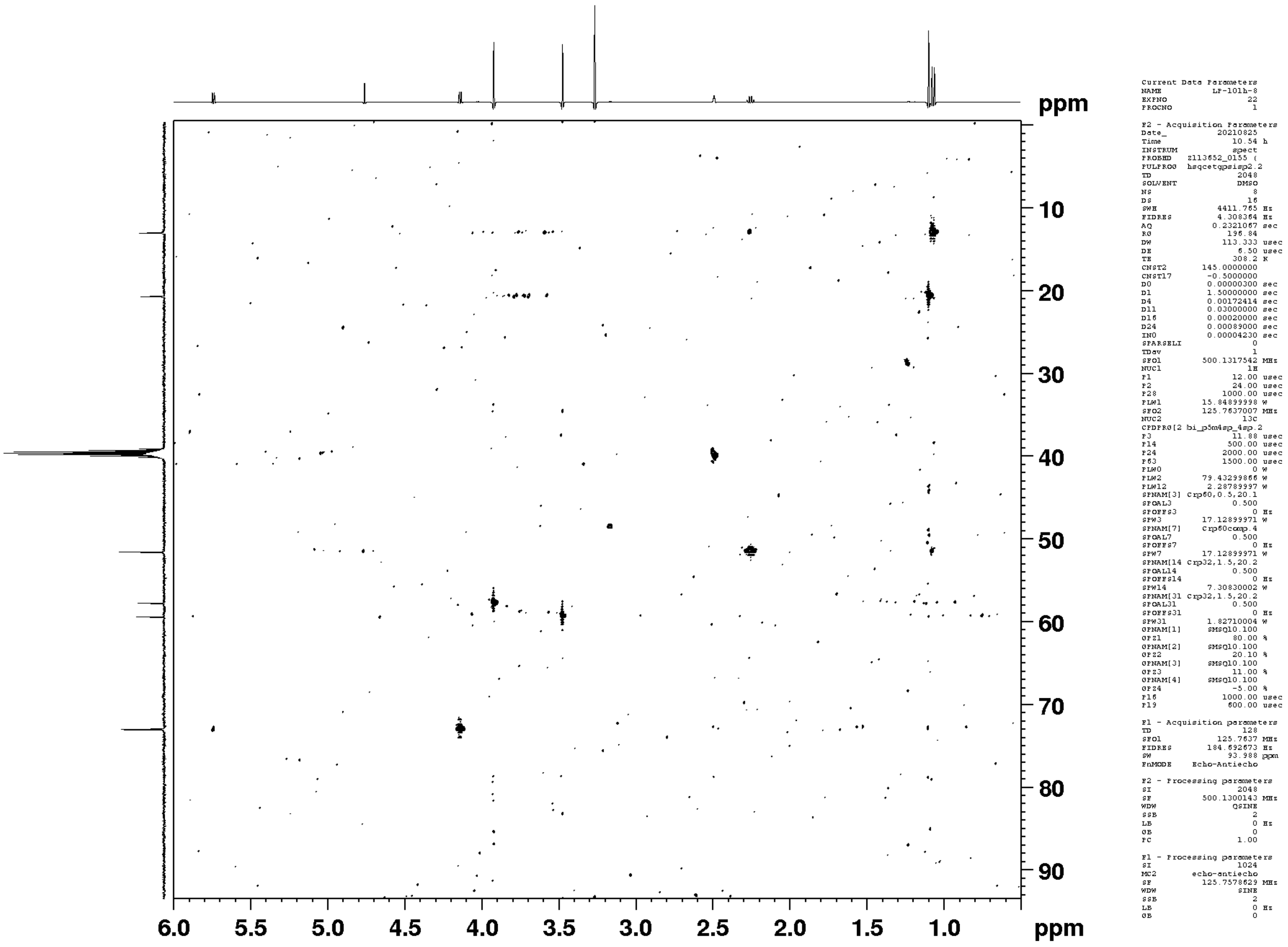

Figure S6. ROESY spectrum of lopouzanone A (1)

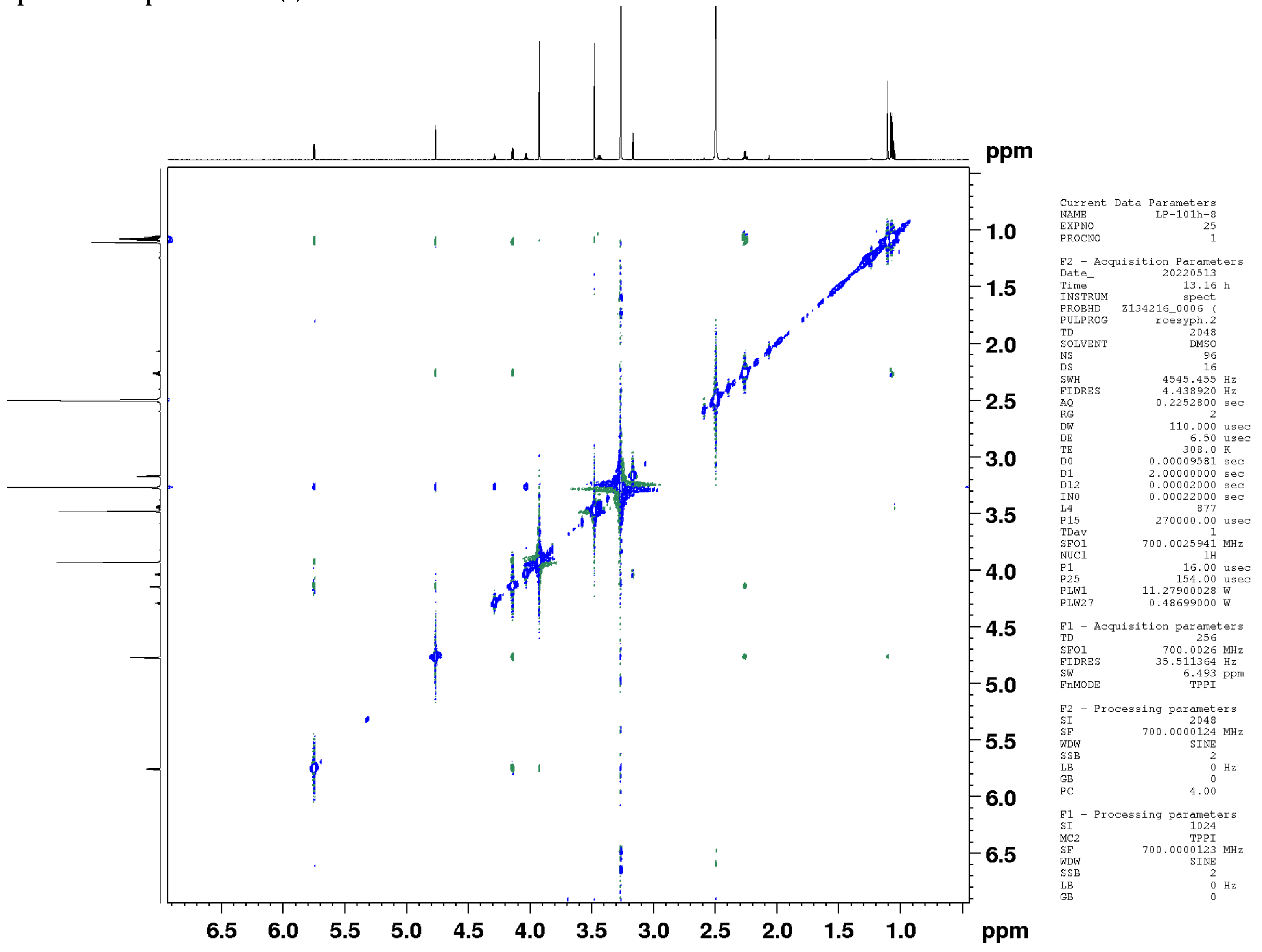

Figure S7. CD spectrum of lopouzanone A (1)

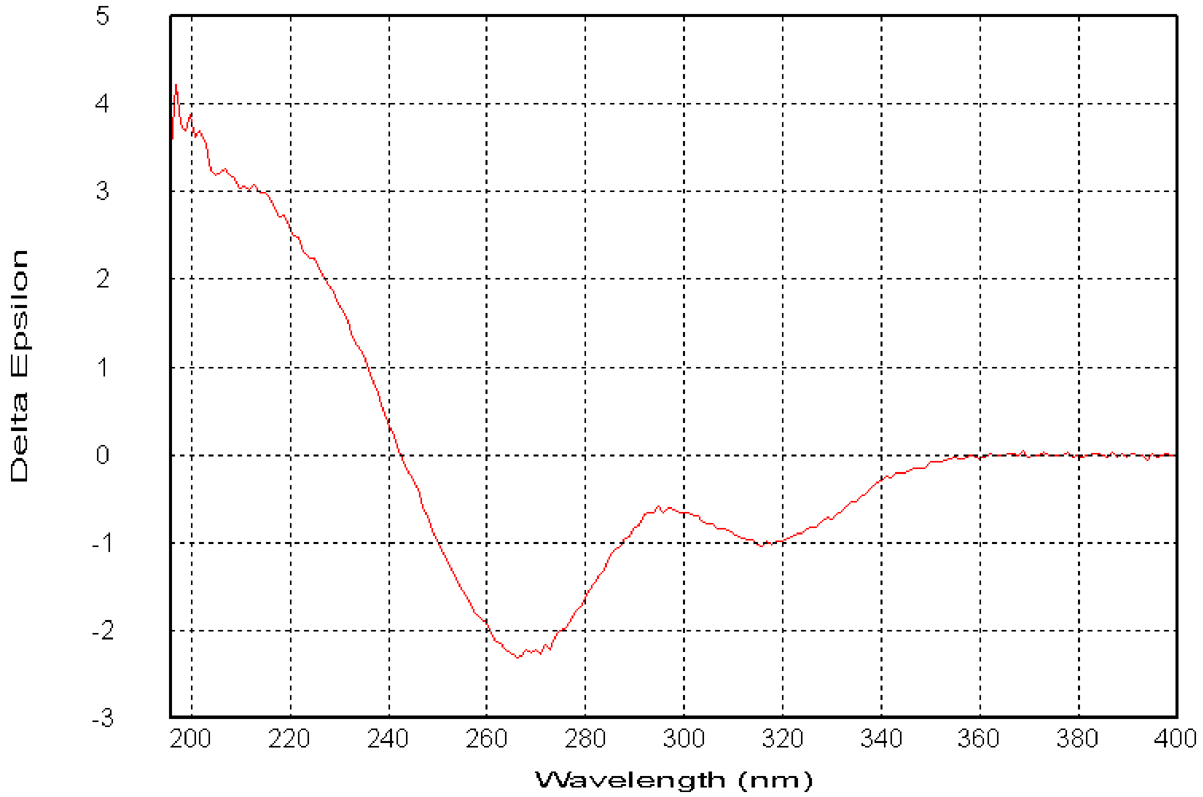

Figure S8. ^{1}H NMR spectrum for (R)-MTPA esters of lopouzanone A (1)

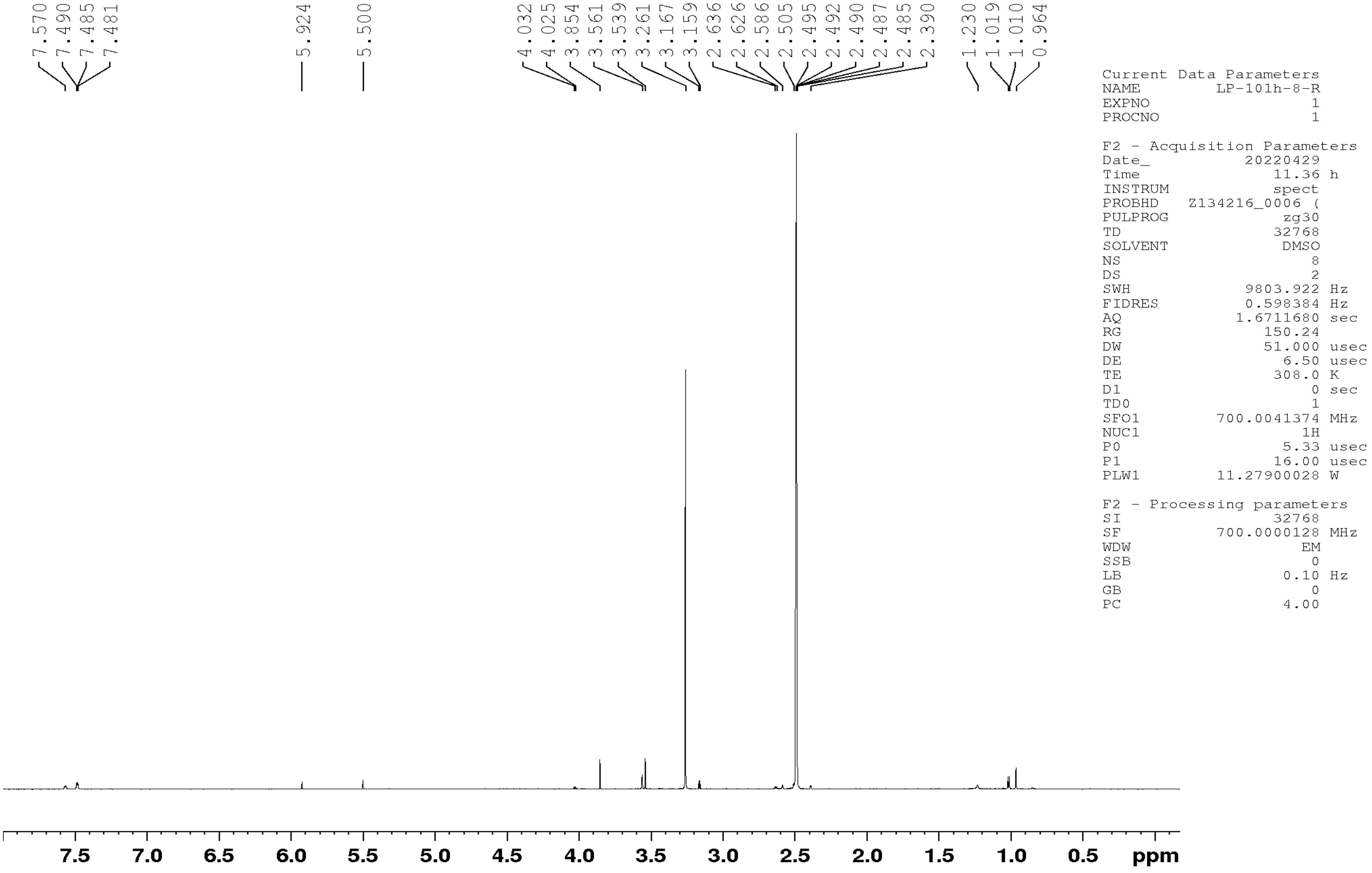

Figure S9. ^{1}H-^{1}H COSY spectrum for (R)-MTPA esters of lopouzanone A (1)

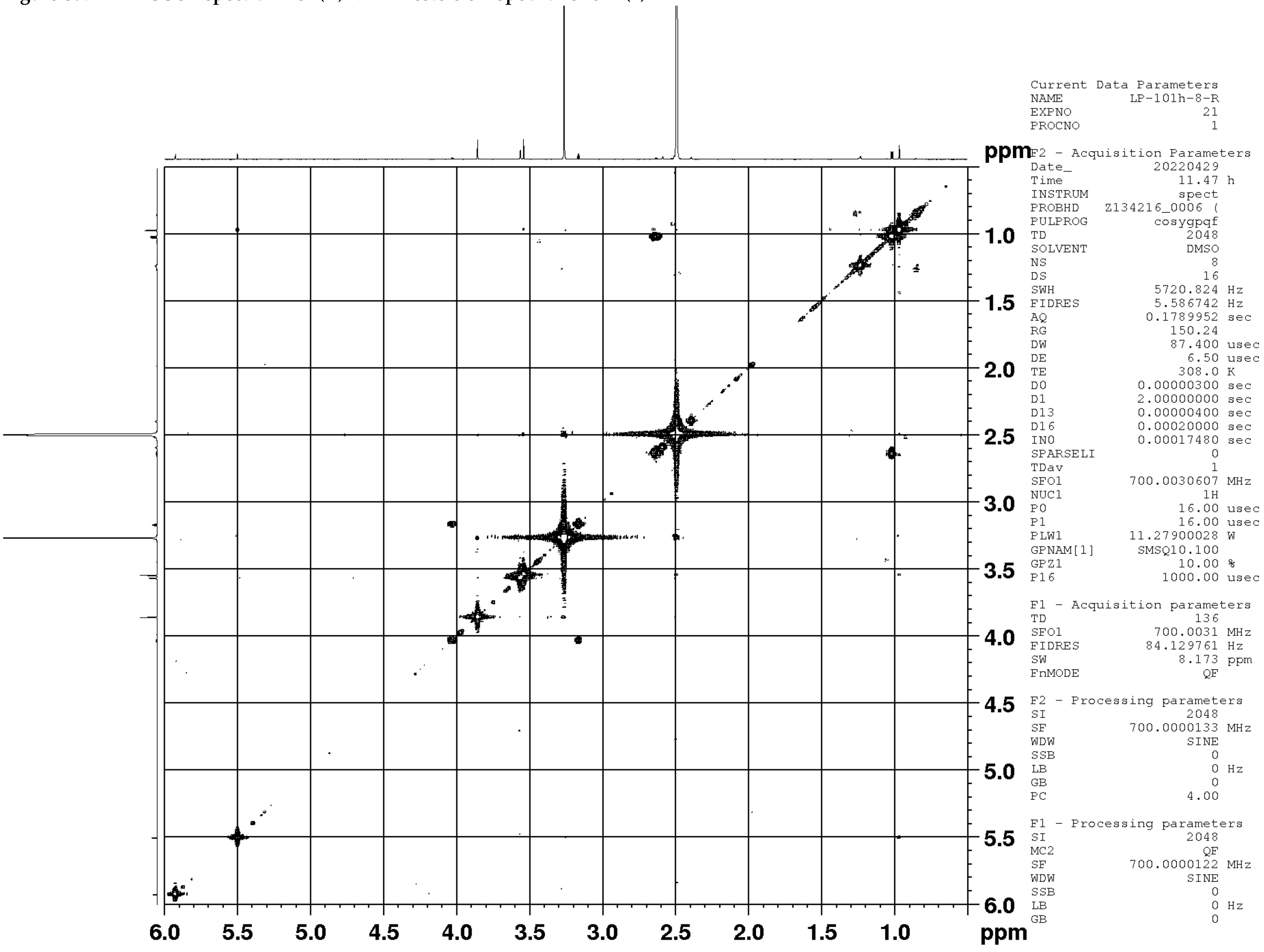

Figure S10. ¹H NMR spectrum for (S)-MTPA esters of lopouzanone A (1)

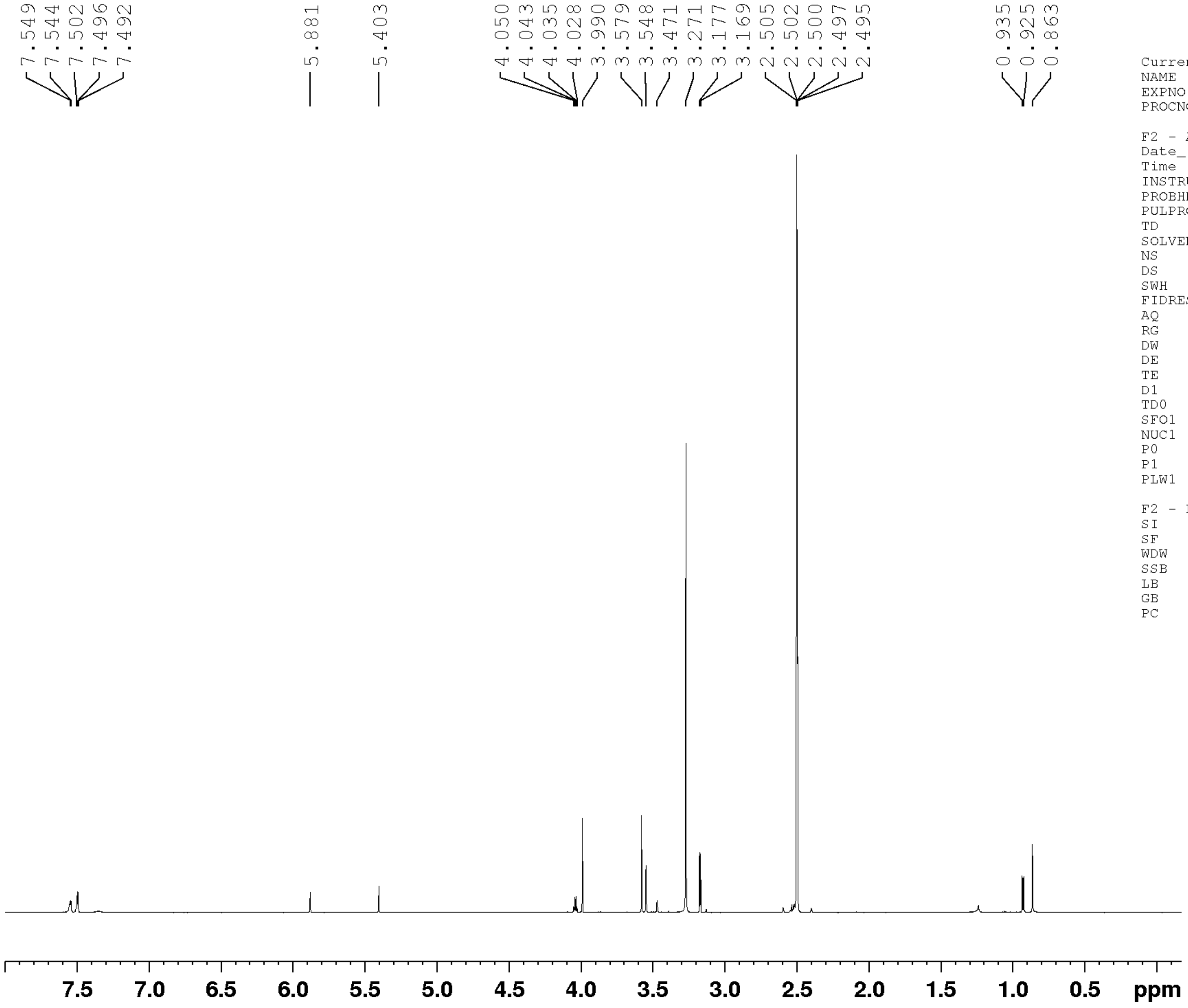

Current Data Parameters
NAME LP-101h-8-S
EXPNO 3
PROCNO 1

F2 - Acquisition Parameters
Date_ 20220429
Time 9.19 h
INSTRUM spect
PROBHD Z134216_0006 (
PULPROG zg30
TD 32768
SOLVENT DMSO
NS 16
DS 2
SWH 9803.922 Hz
FIDRES 0.598384 Hz
AQ 1.6711680 sec
RG 150.24
DW 51.000 usec
DE 6.50 usec
TE 308.0 K
D1 0 sec
TD0 1
SFO1 700.0041374 MHz
NUC1 1H
P0 5.33 usec
P1 16.00 usec
PLW1 11.27900028 W

F2 - Processing parameters
SI 32768
SF 700.0000058 MHz
WDW EM
SSB 0
LB 0.10 Hz
GB 0
PC 4.00

Figure S11. COSY spectrum for (S)-MTPA esters of lopouzanone A (1)

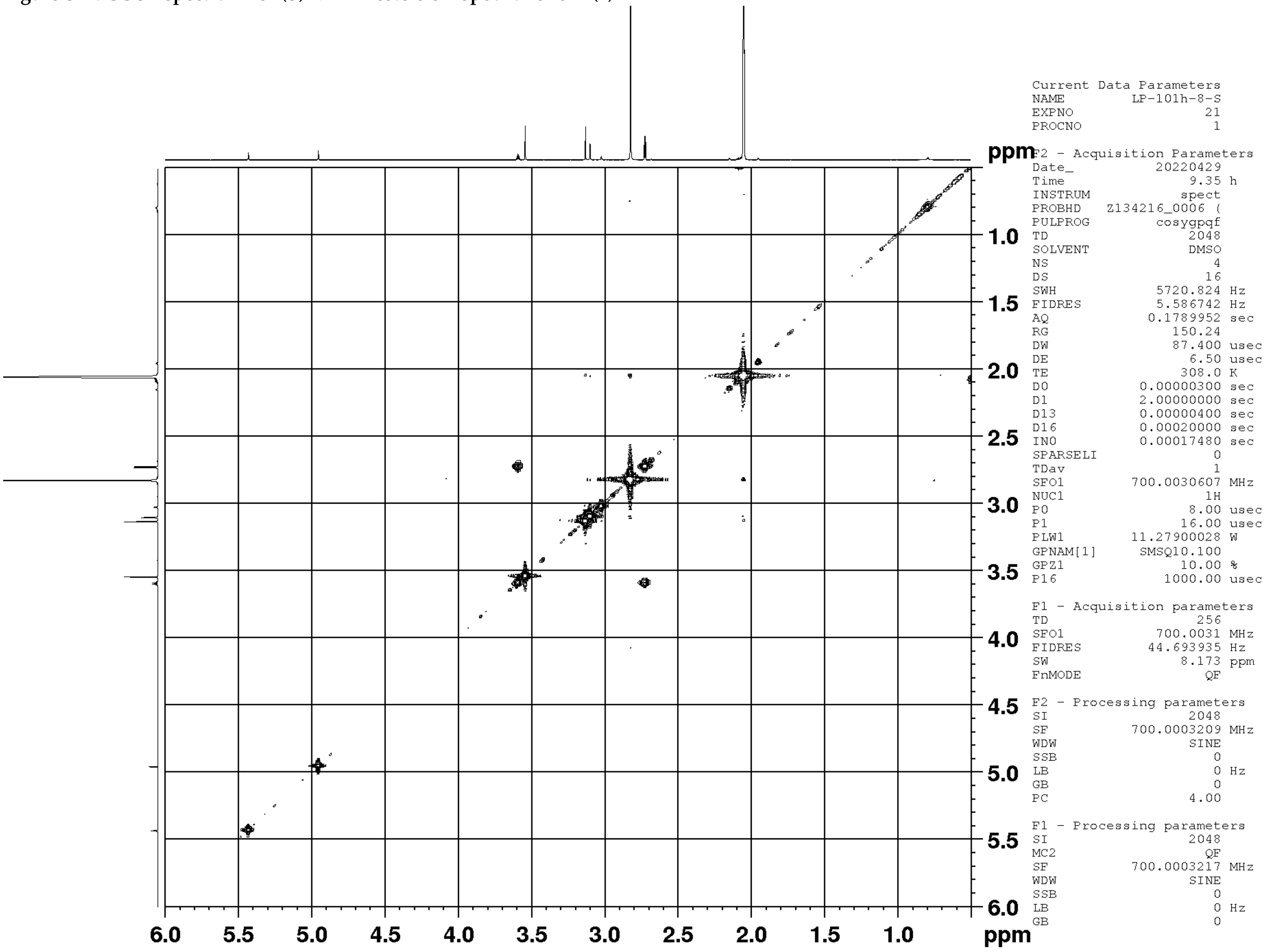

Figure S12. ^{1}H NMR spectrum of lopouzanone B (2)

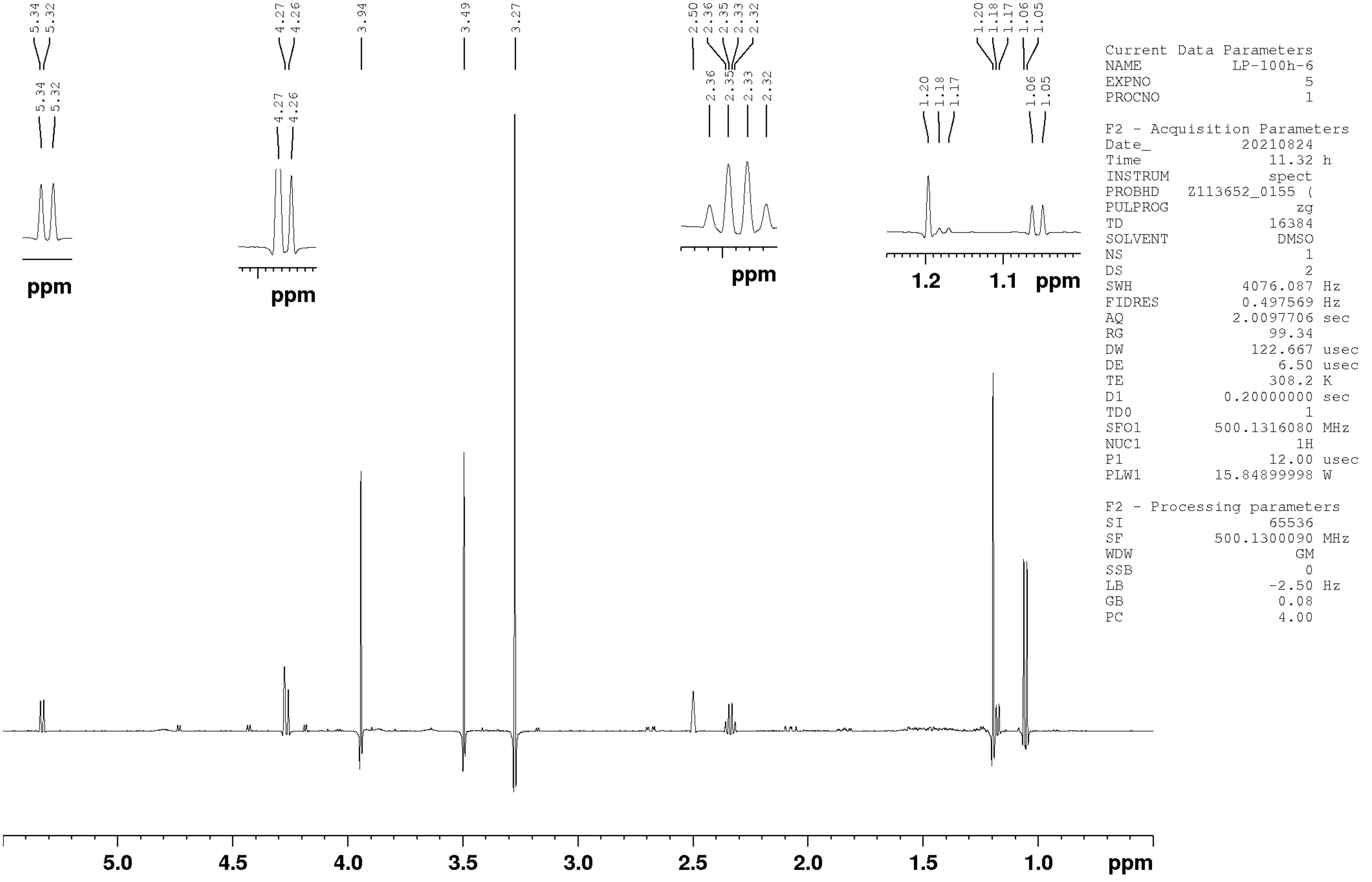

Figure S13. ^{13}C NMR spectrum of lopouzanone B (2)

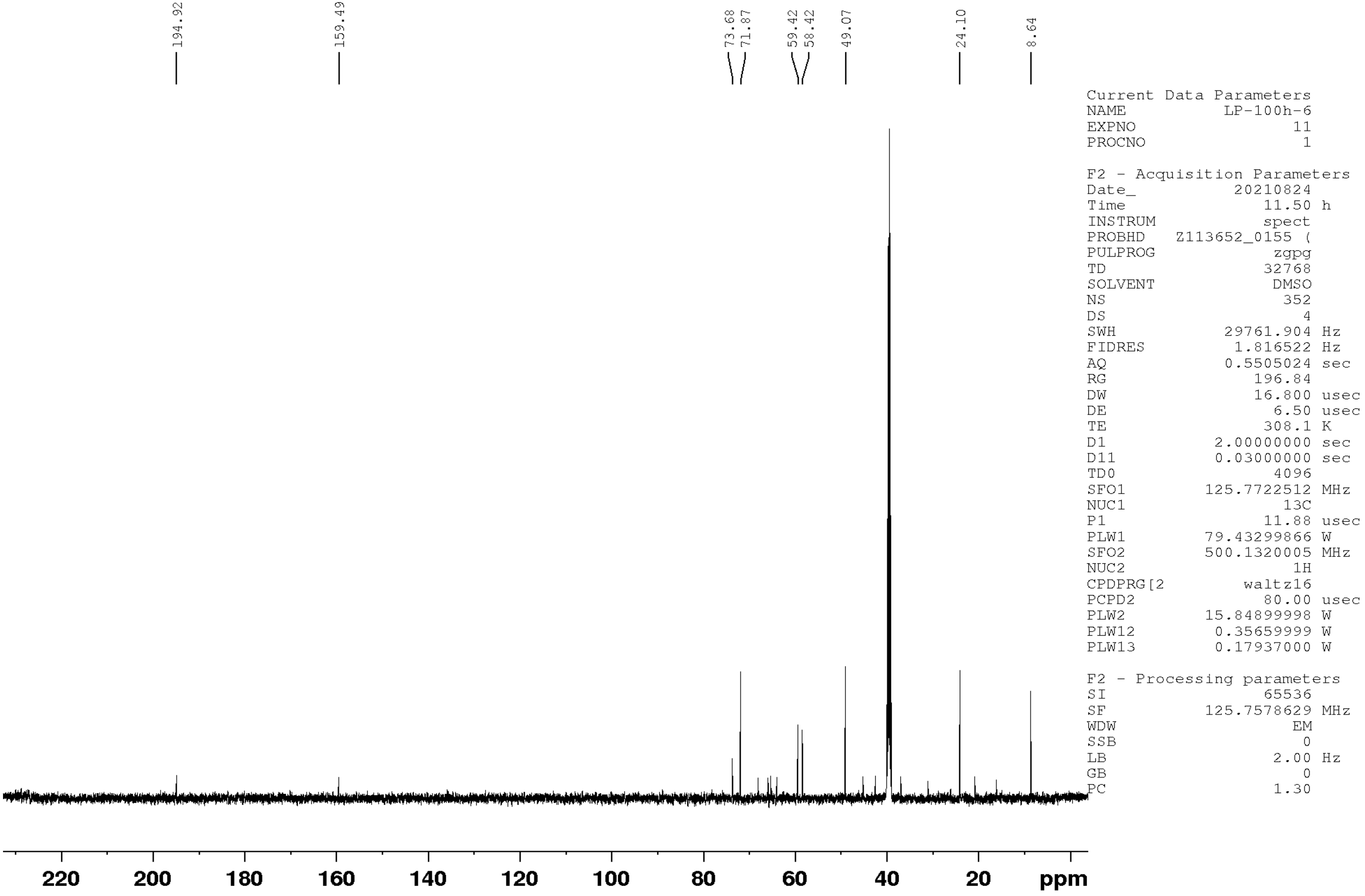

Figure S14. ^{1}H-^{1}H COSY spectrum of lopouzanone B (2)

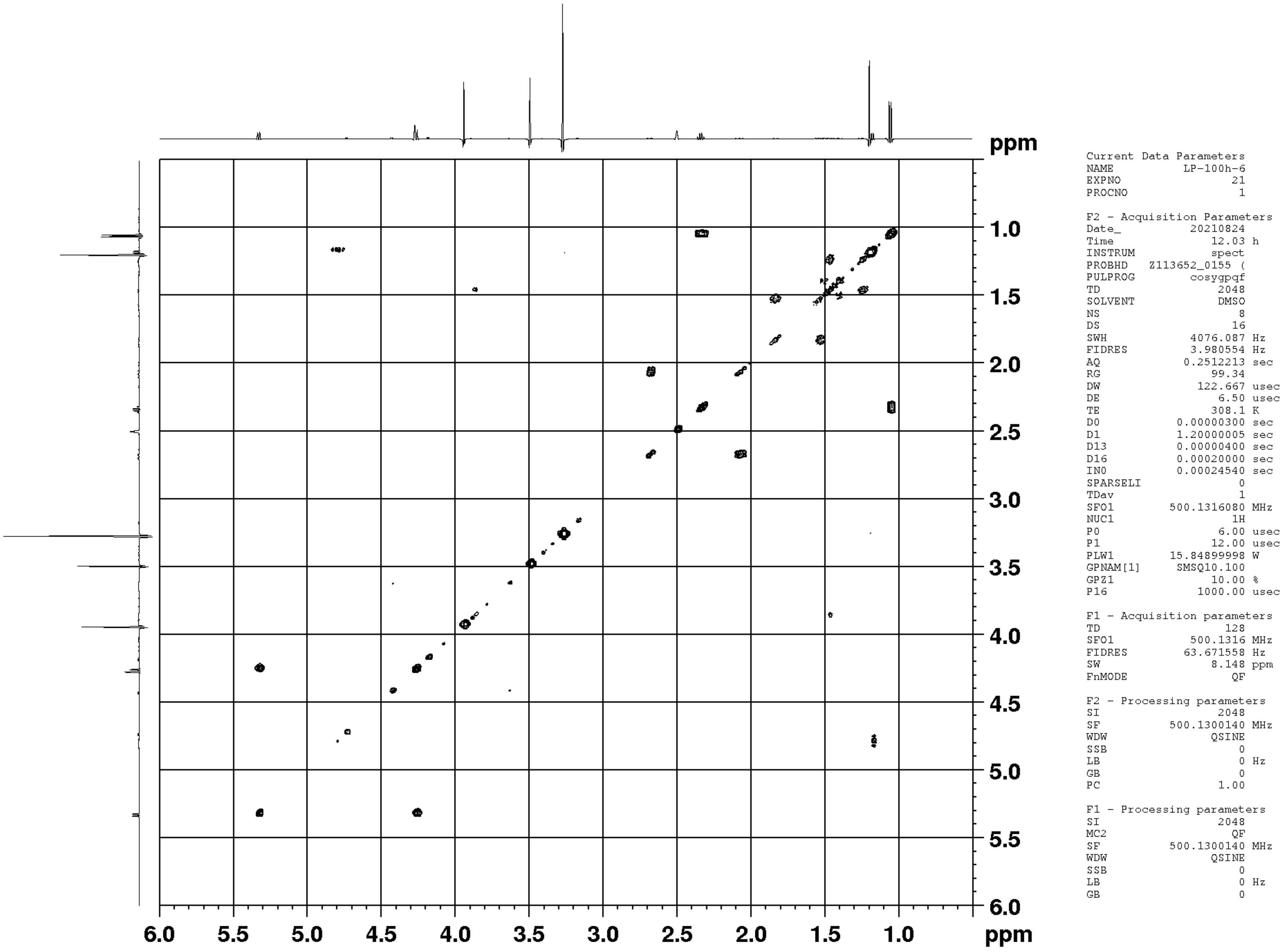

Figure S15. HMBC spectrum of lopouzanone B (2)

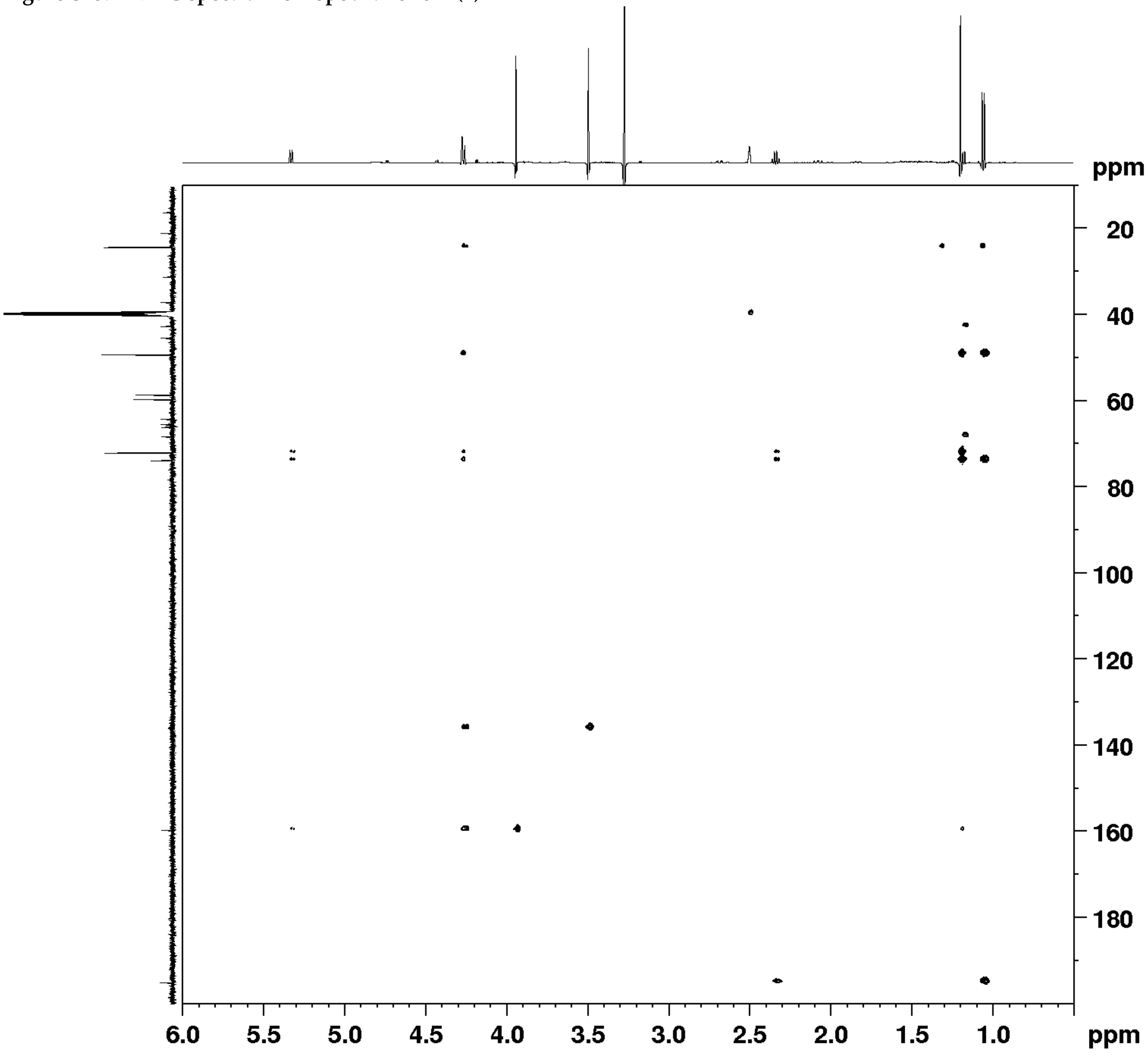


```
Current Data Parameters
NAME          LP-100h-6
EXPNO                24
PROCNO                1

F2 - Acquisition Parameters
Date_          20210824
Time              16.31 h
INSTRUM           spect
PROBHD   Z113652_0155 (
PULPROG    hmbcgplpndqf
TD                 2048
SOLVENT            DMSO
NS                   64
DS                   16
SWH            4076.087 Hz
FIDRES         3.980554 Hz
AQ            0.2512213 sec
RG               196.84
DW              122.667 usec
DE                 6.50 usec
TE                308.2 K
CNST2       145.0000000
CNST13        5.0000000
D0           0.00000300 sec
D1           1.50000000 sec
D2           0.00344828 sec
D6           0.10000000 sec
D16          0.00020000 sec
IN0          0.00001860 sec
SPARSELI              0
TDav                  1
SFO1        500.1316080 MHz
NUC1                 1H
P1                12.00 usec
P2                24.00 usec
PLW1        15.84899998 W
SFO2        125.7712355 MHz
NUC2                13C
P3                11.88 usec
PLW2        79.43299866 W
GPNAM[1]     SMSQ10.100
GPZ1              50.00 %
GPNAM[2]     SMSQ10.100
GPZ2              30.00 %
GPNAM[3]     SMSQ10.100
GPZ3              40.10 %
P16             1000.00 usec

F1 - Acquisition parameters
TD                  128
SFO1           125.7712 MHz
FIDRES       420.026886 Hz
SW              213.735 ppm
FnMODE               QF

F2 - Processing parameters
SI                 2048
SF          500.1300140 MHz
WDW               QSINE
SSB                   3
LB                    0 Hz
GB                    0
PC                 2.00

F1 - Processing parameters
SI                 1024
MC2                  QF
SF          125.7578629 MHz
WDW                SINE
SSB                   0
LB                    0 Hz
GB                    0
```

Figure S16. HSQC spectrum of lopouzanone B (2)

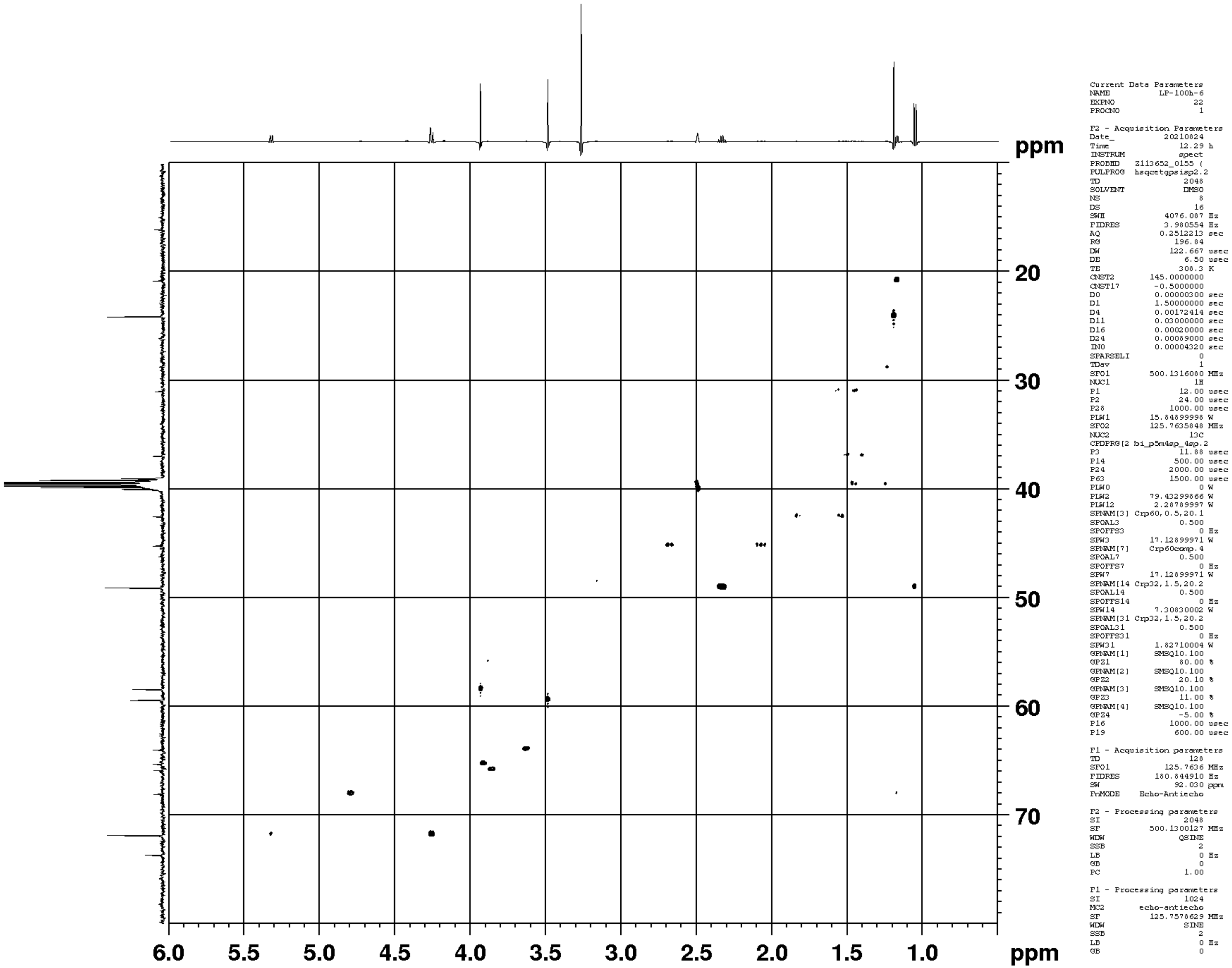

Figure S17. ROESY spectrum of lopouzanone B (2)

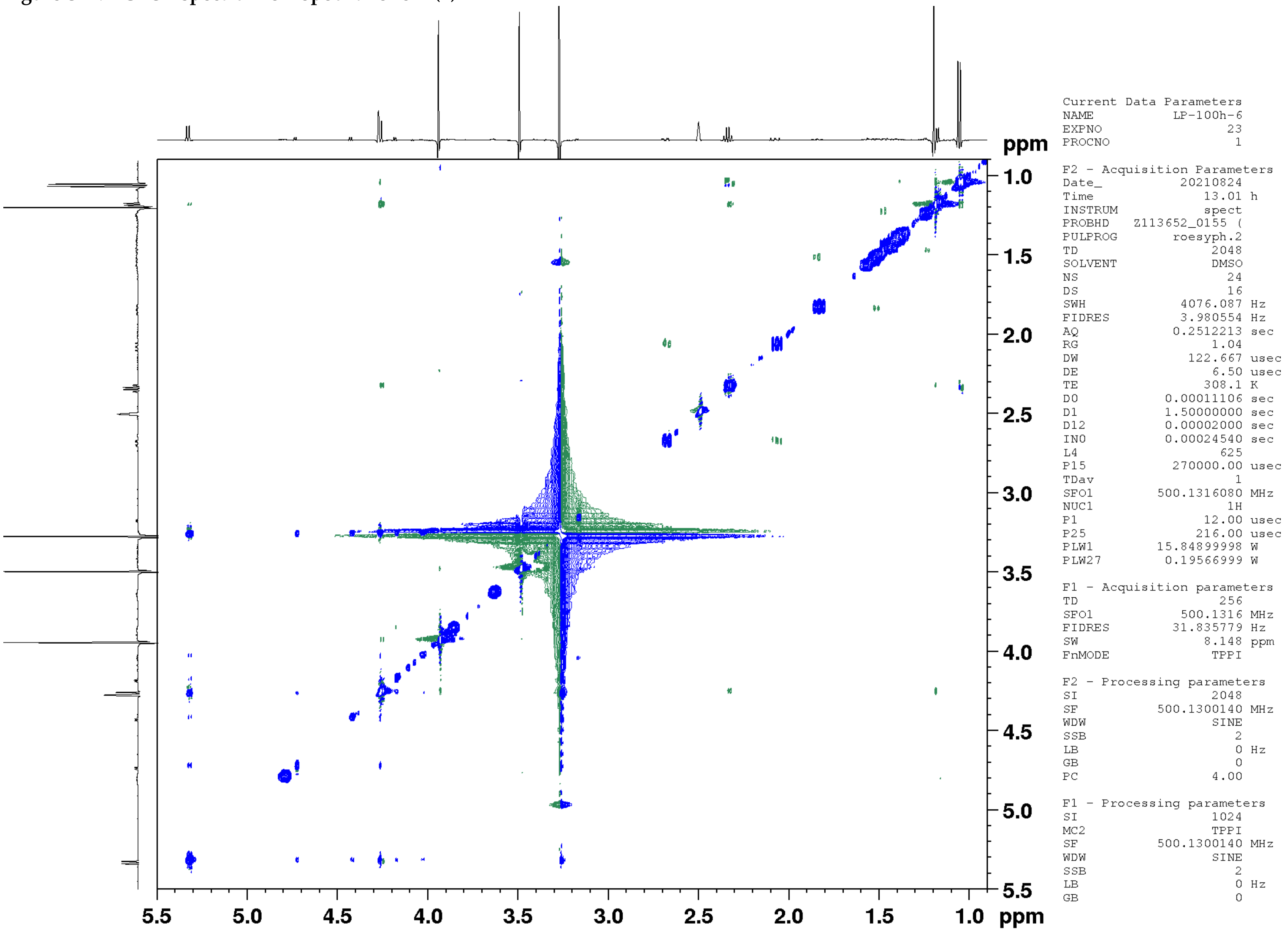

Figure S18. CD spectrum of lopouzanone B (2)

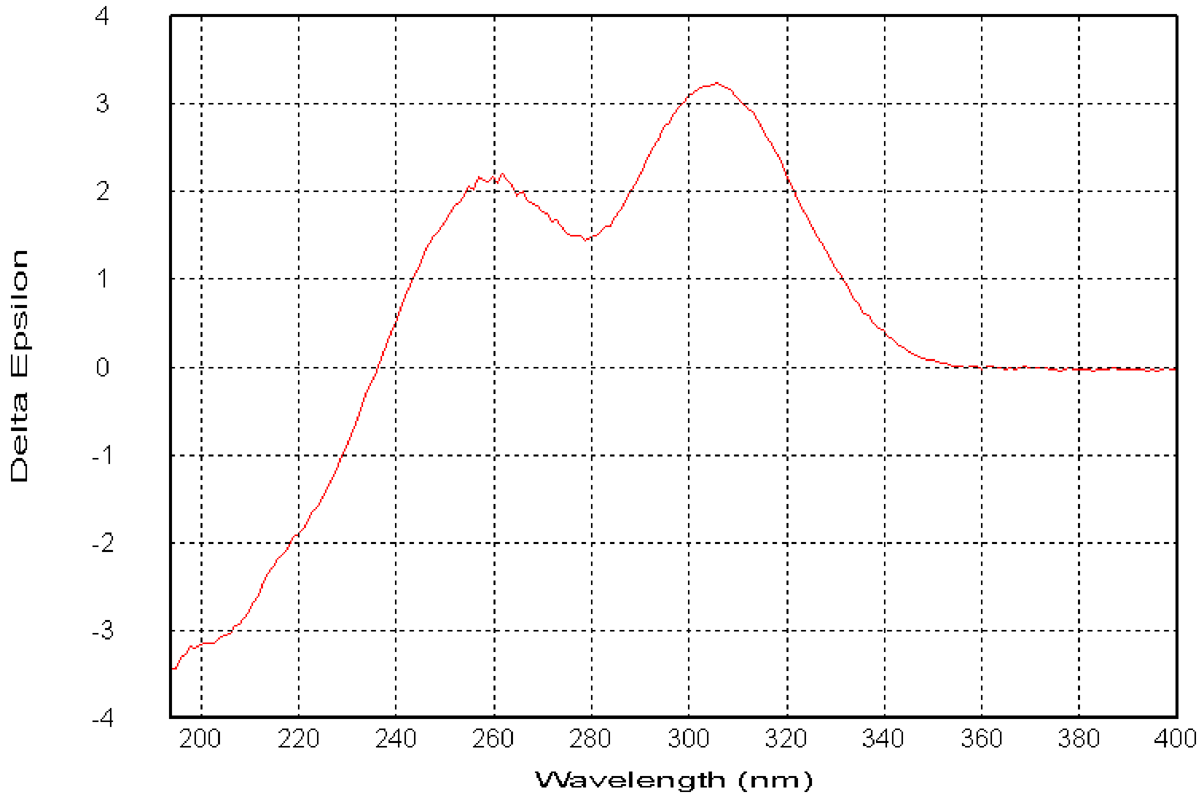

Figure S19. ^{1}H NMR spectrum for (R)-MTPA esters of lopouzanone B (2)

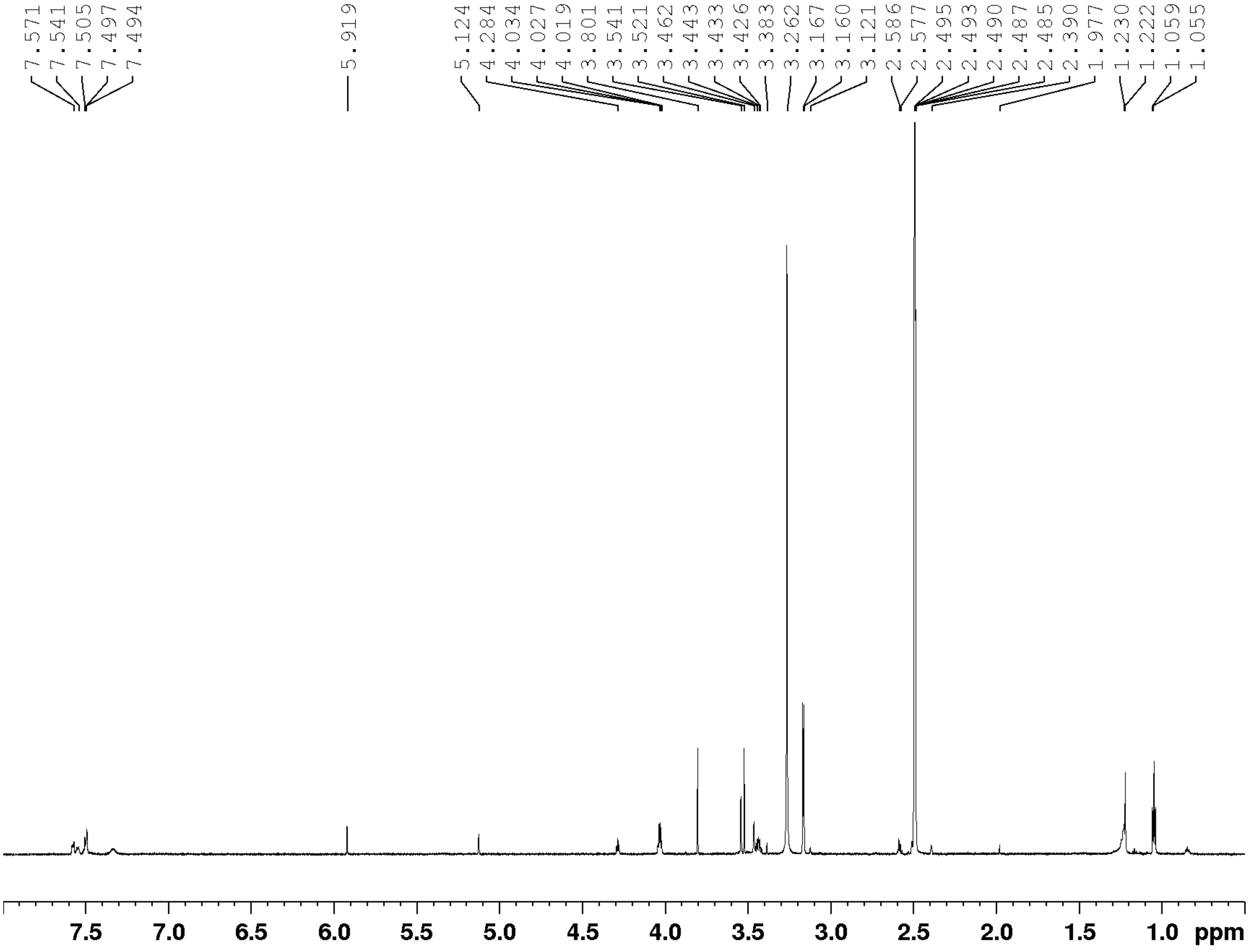


```
Current Data Parameters
NAME        LP-107h-1-S
EXPNO                 5
PROCNO                1

F2 - Acquisition Parameters
Date_          20220429
Time              10.32 h
INSTRUM           spect
PROBHD   Z134216_0006 (
PULPROG            zg30
TD                32768
SOLVENT            DMSO
NS                    2
DS                    2
SWH            5707.763 Hz
FIDRES         0.348374 Hz
AQ            2.8704767 sec
RG               150.24
DW               87.600 usec
DE                 6.50 usec
TE                308.0 K
D1                    0 sec
TD0                   1
SFO1       700.0031840 MHz
NUC1                 1H
P0                 5.33 usec
P1                16.00 usec
PLW1        11.27900028 W

F2 - Processing parameters
SI                32768
SF         700.0000127 MHz
WDW                  EM
SSB                   0
LB                 0.10 Hz
GB                    0
PC                 4.00
```

Figure S20. ¹H-¹H COSY spectrum for (R)-MTPA esters of lopouzanone B (2)

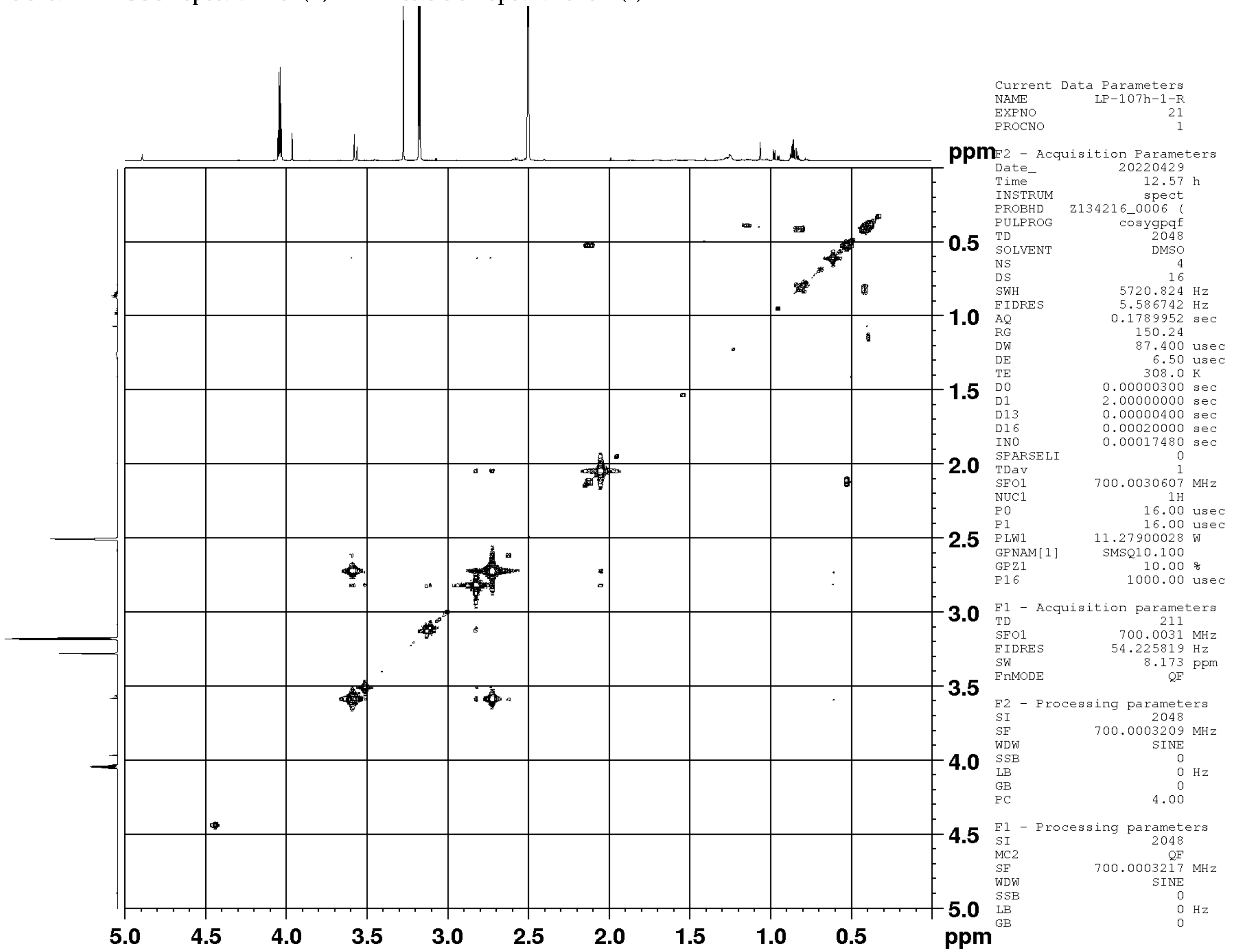

Figure S21. ¹H NMR spectrum for (S)-MTPA esters of lopouzanone B (2)

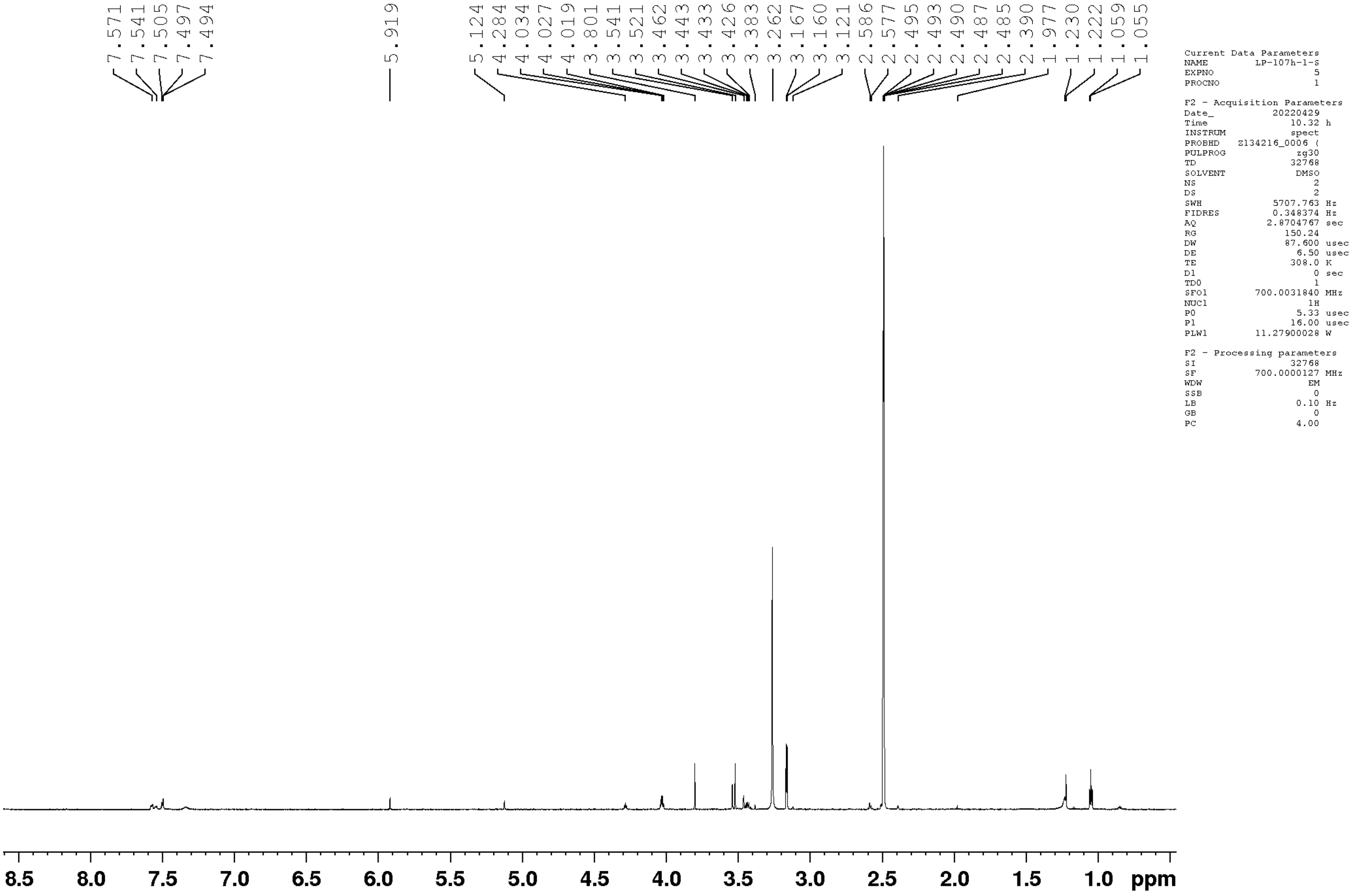

Figure S22. ^{1}**H-**^{1}**H COSY spectrum for (S)-MTPA esters of lopouzanone B (2)**

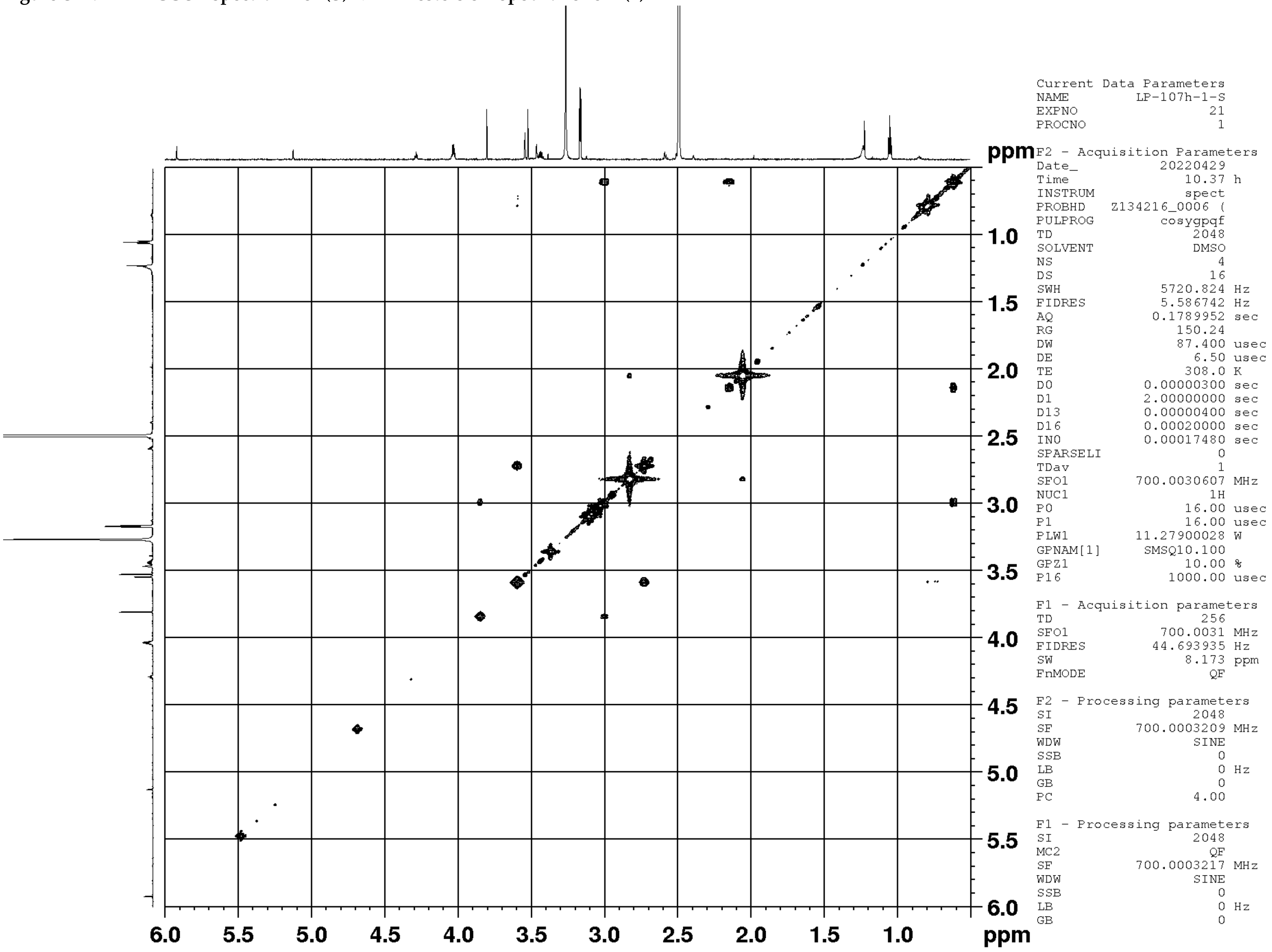

Figure S23. ¹H NMR spectrum of gliorosein (3)

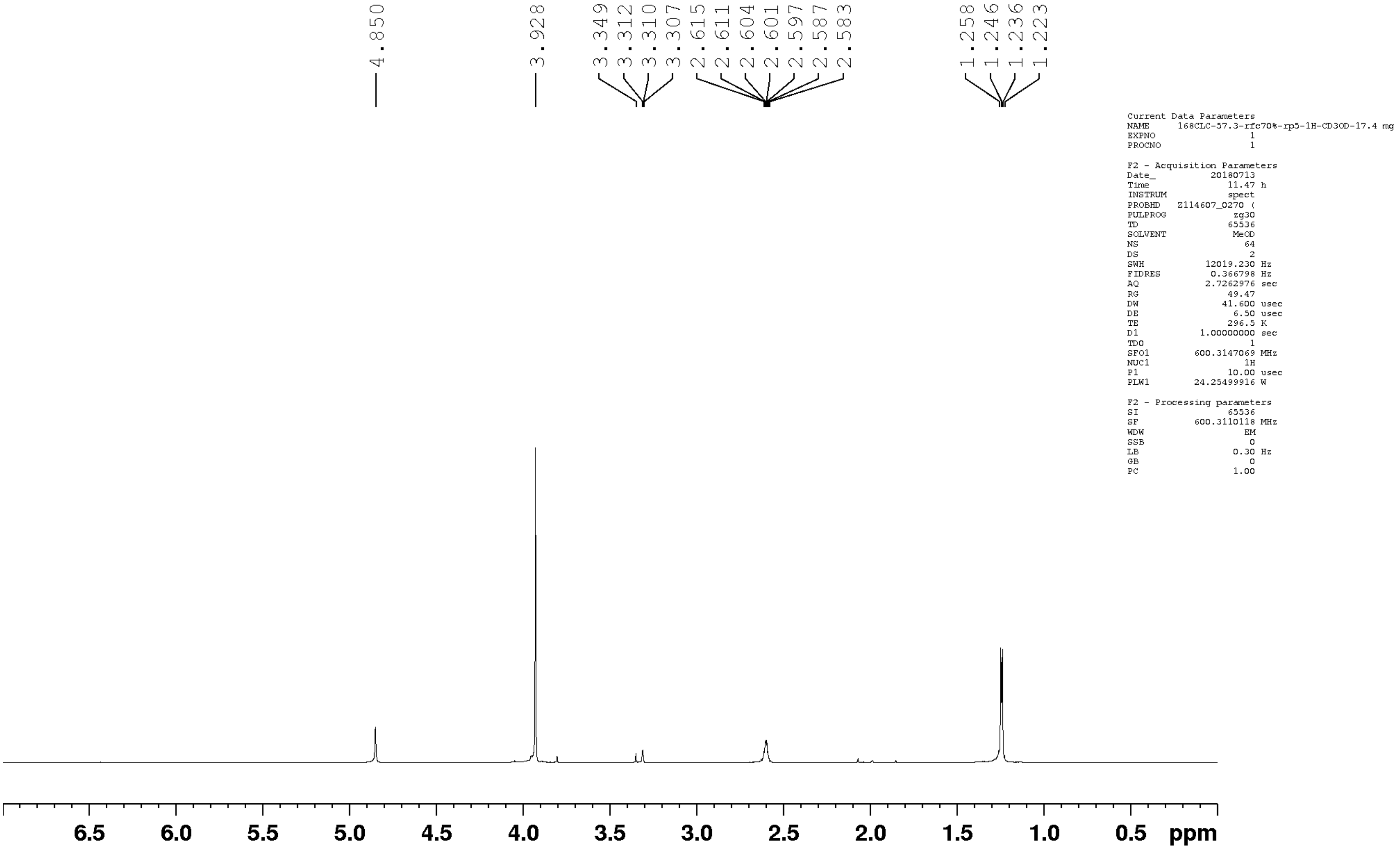

Figure S24. ^{13}C NMR spectrum of gliorosein (3)

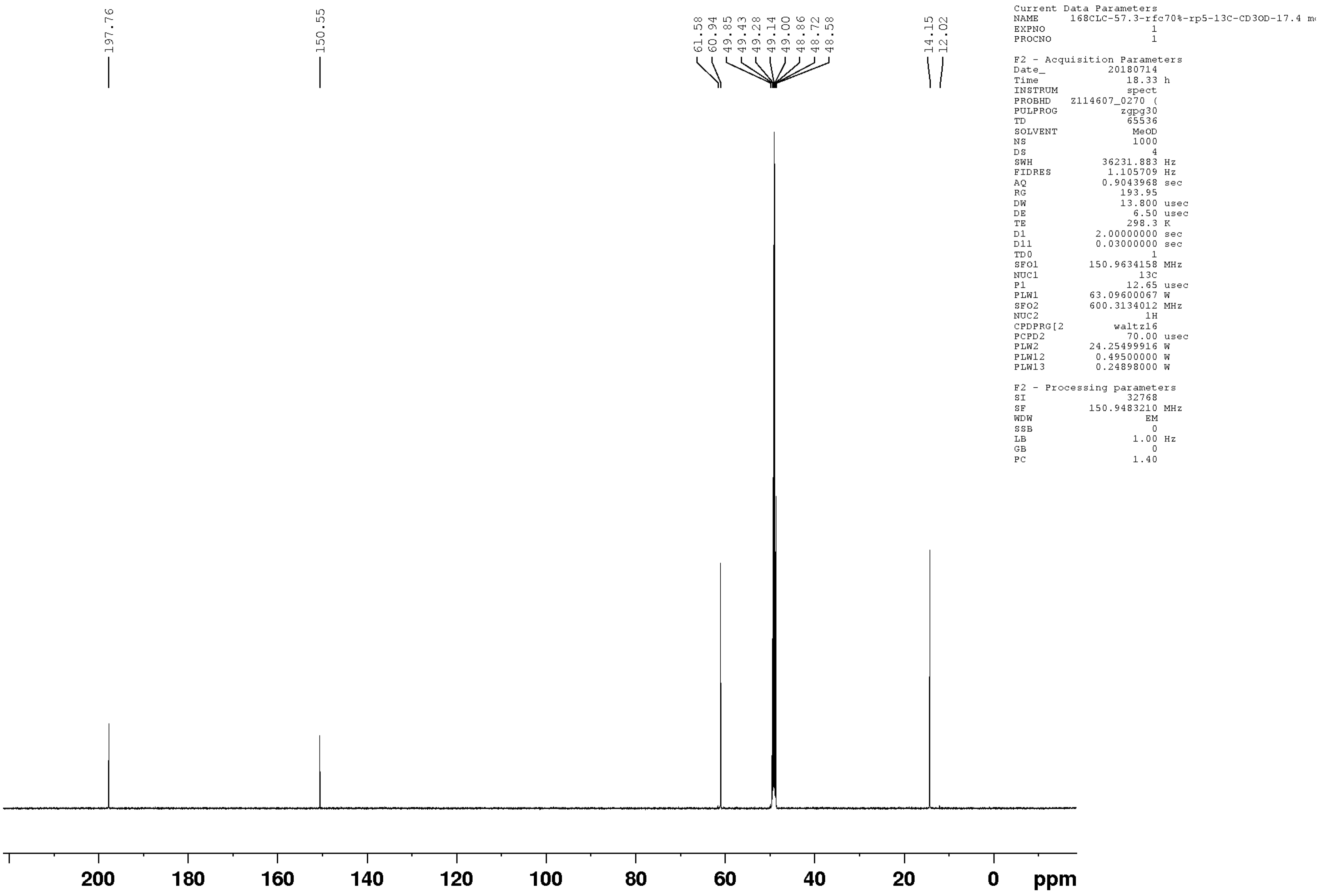

Figure S25. CD spectrum of gliorosein (3)

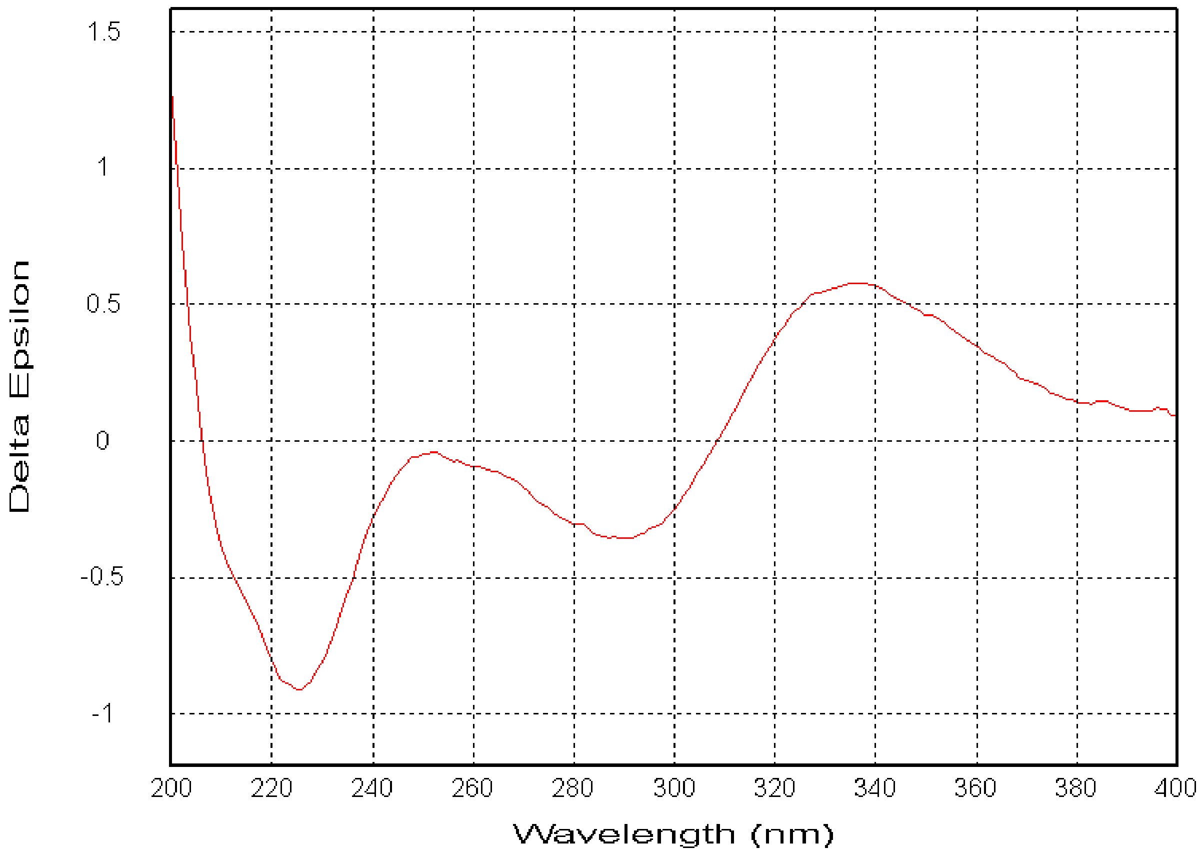

Figure S26. ^1H NMR spectrum of balticolid (4)

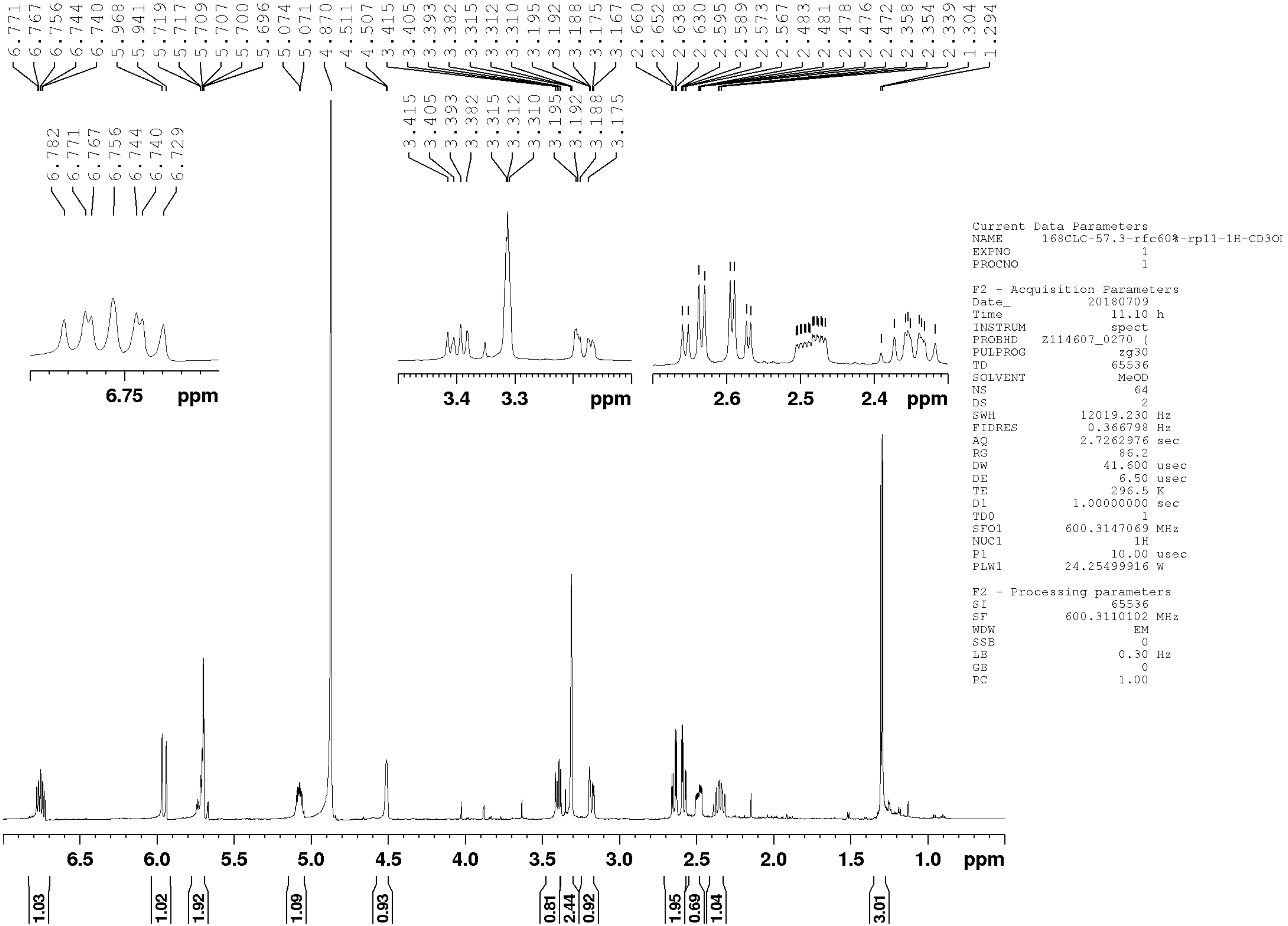

Figure S27. ^{13}C NMR spectrum of balticolid (4)

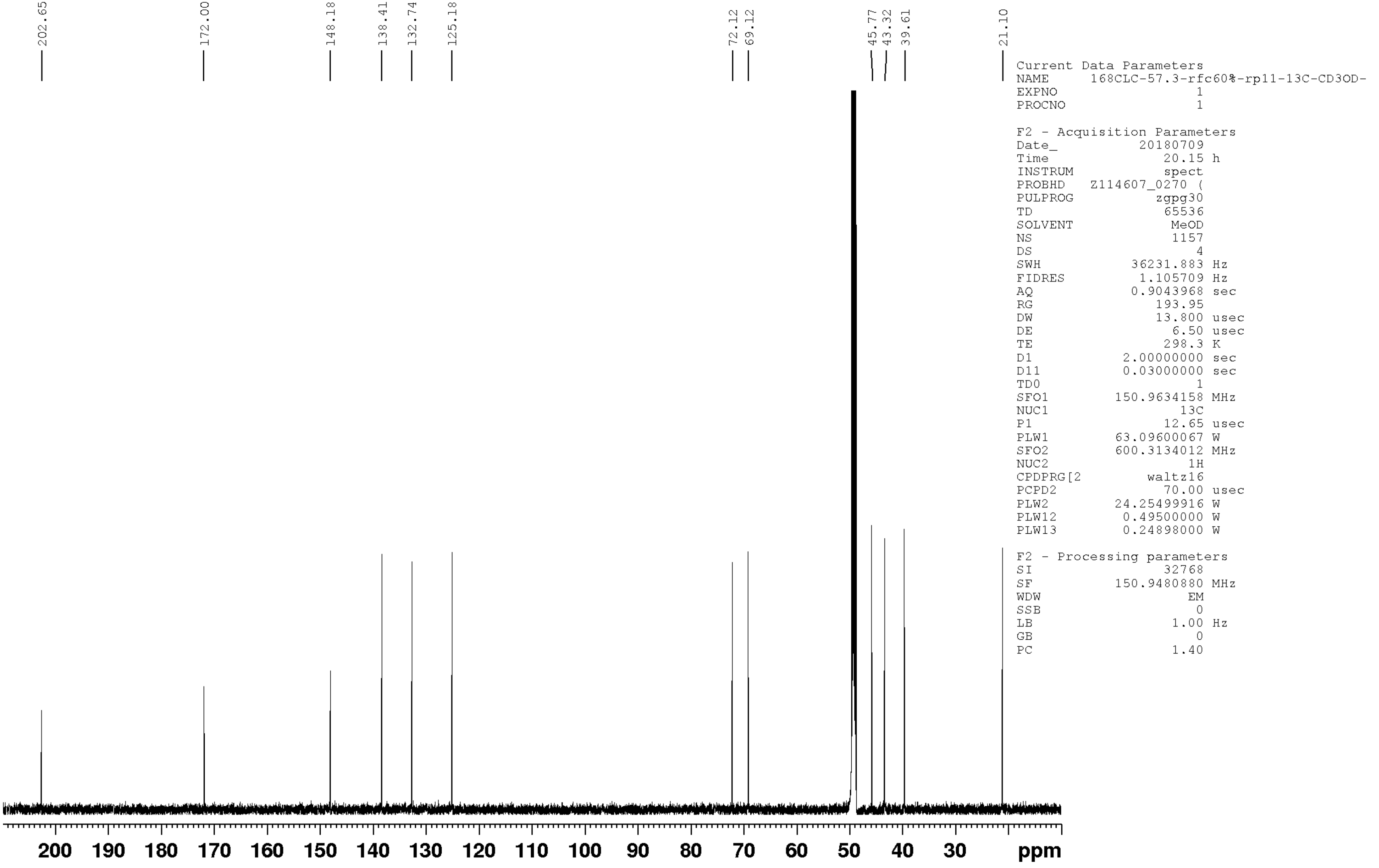

Figure S28. ^{1}H NMR spectrum of dendrodolide G (5)

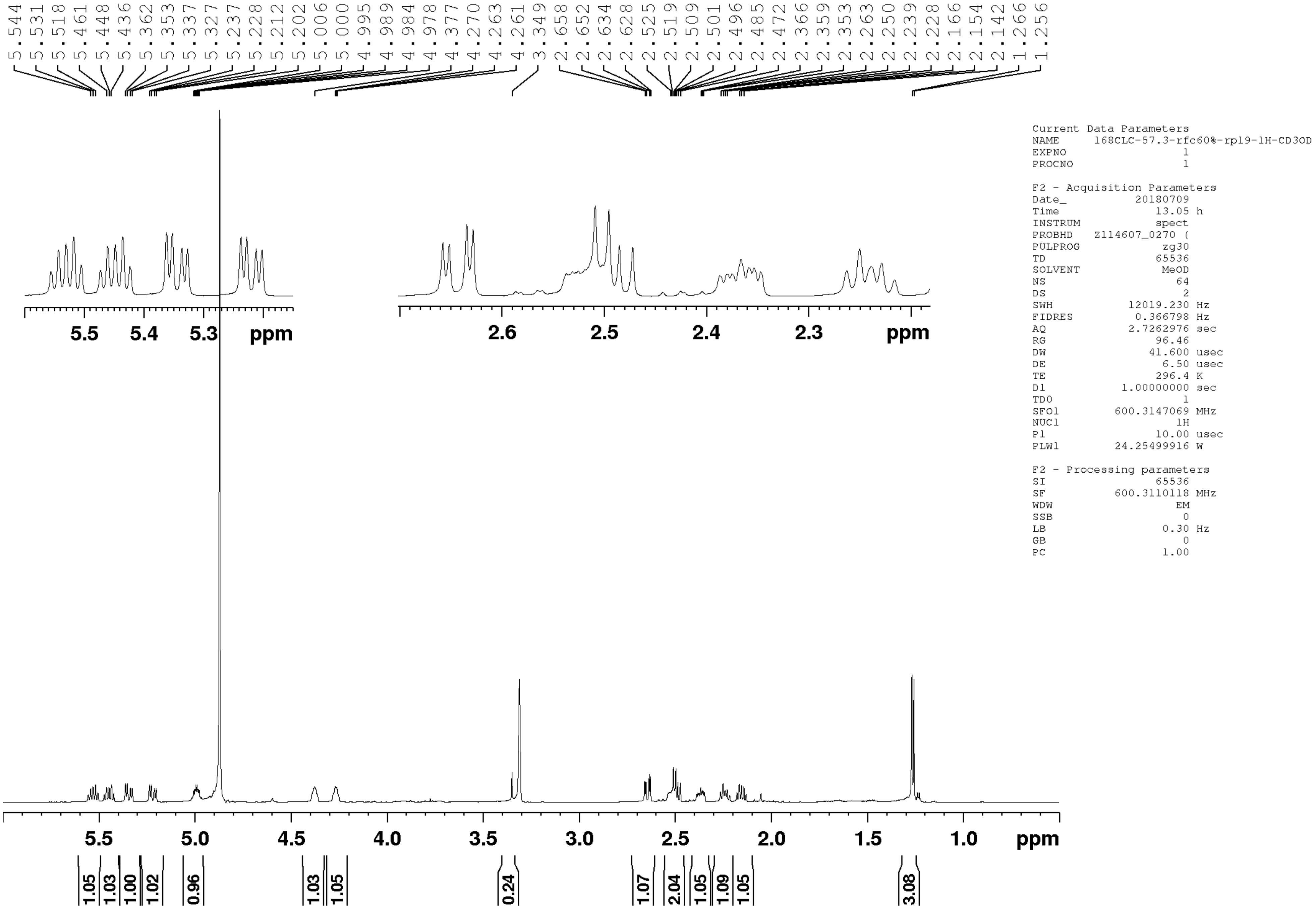

Figure S29. ^{13}C NMR spectrum of dendrodolide G (5)

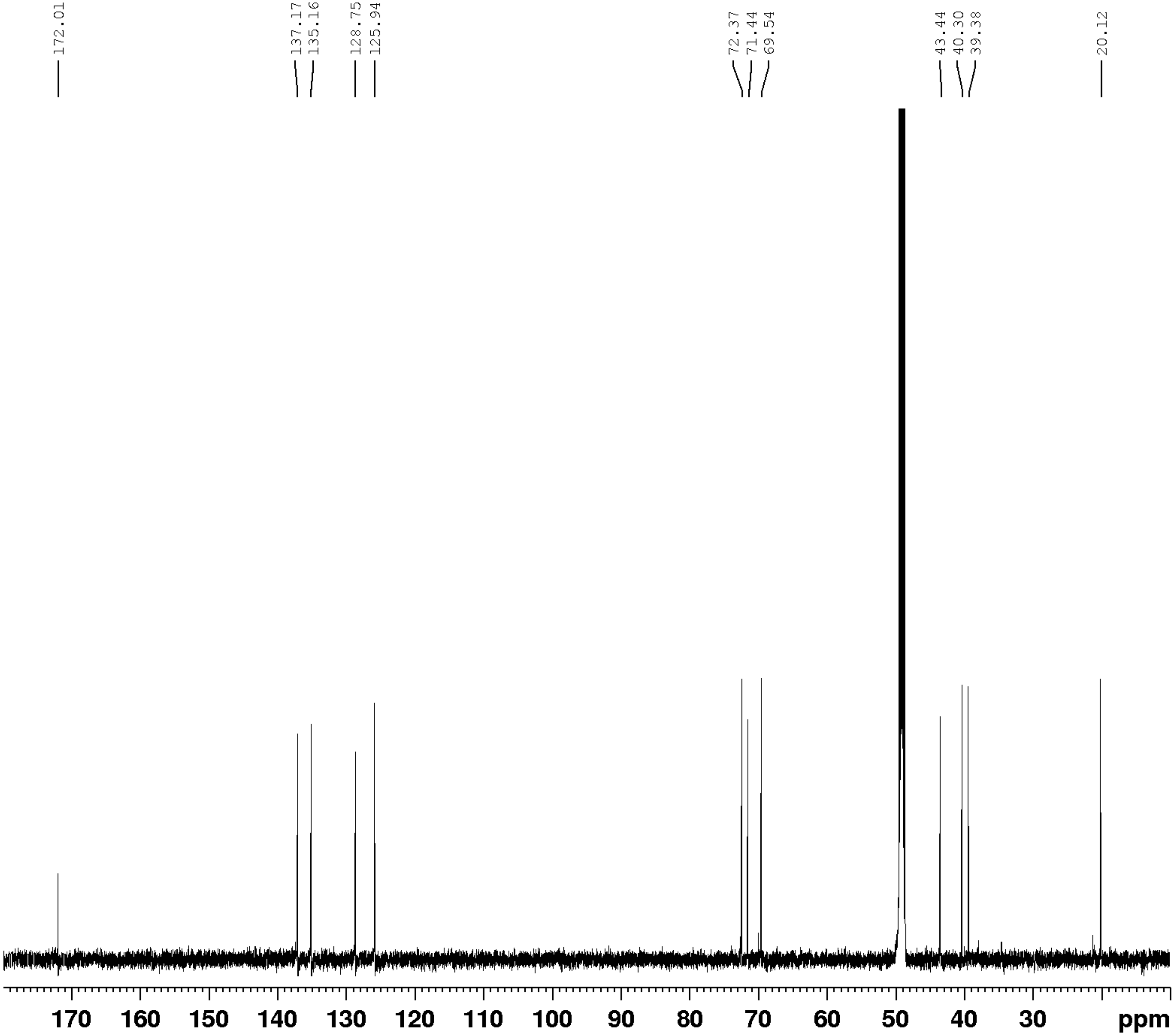


```
Current Data Parameters
NAME     168CLC-57.3-rfc60%-rp19-13C-CD3OD
EXPNO                    1
PROCNO                   1

F2 - Acquisition Parameters
Date_           20180710
Time                0.25 h
INSTRUM            spect
PROBHD   Z114607_0270 (
PULPROG          zgpg30
TD                 65536
SOLVENT             MeOD
NS                  1165
DS                     4
SWH           36231.883 Hz
FIDRES         1.105709 Hz
AQ            0.9043968 sec
RG                193.95
DW                13.800 usec
DE                  6.50 usec
TE                 298.4 K
D1            2.00000000 sec
D11           0.03000000 sec
TD0                    1
SFO1         150.9634158 MHz
NUC1                 13C
P1                 12.65 usec
PLW1         63.09600067 W
SFO2         600.3134012 MHz
NUC2                  1H
CPDPRG[2         waltz16
PCPD2              70.00 usec
PLW2         24.25499916 W
PLW12         0.49500000 W
PLW13         0.24898000 W

F2 - Processing parameters
SI                 32768
SF           150.9481099 MHz
WDW                   EM
SSB                    0
LB                  1.00 Hz
GB                     0
PC                  1.40
```

Figure S30. ^{1}H NMR spectrum of dihydroisocoumarine (6)

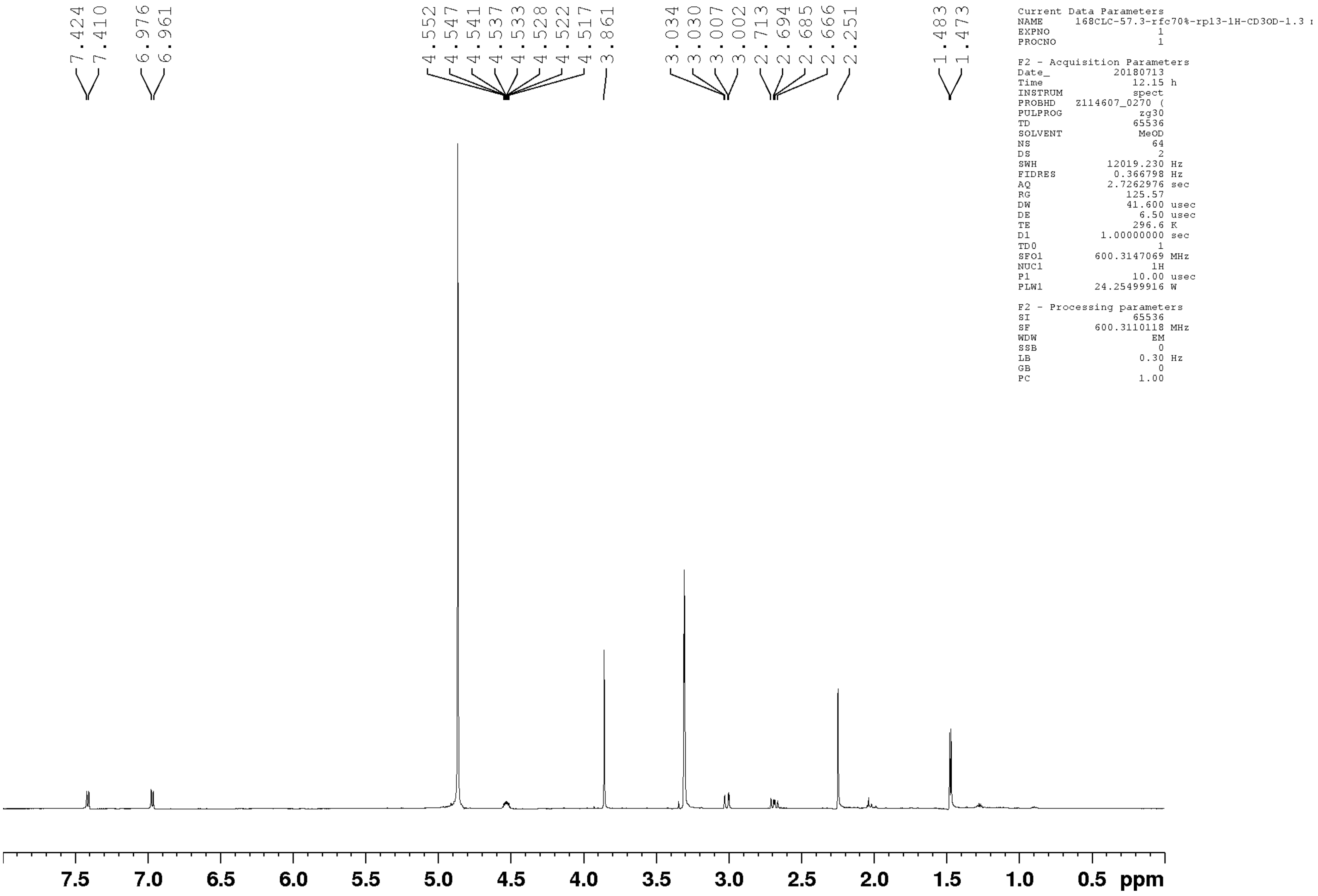

Figure S31. ^{13}C **NMR spectrum of dihydroisocoumarine (6)**

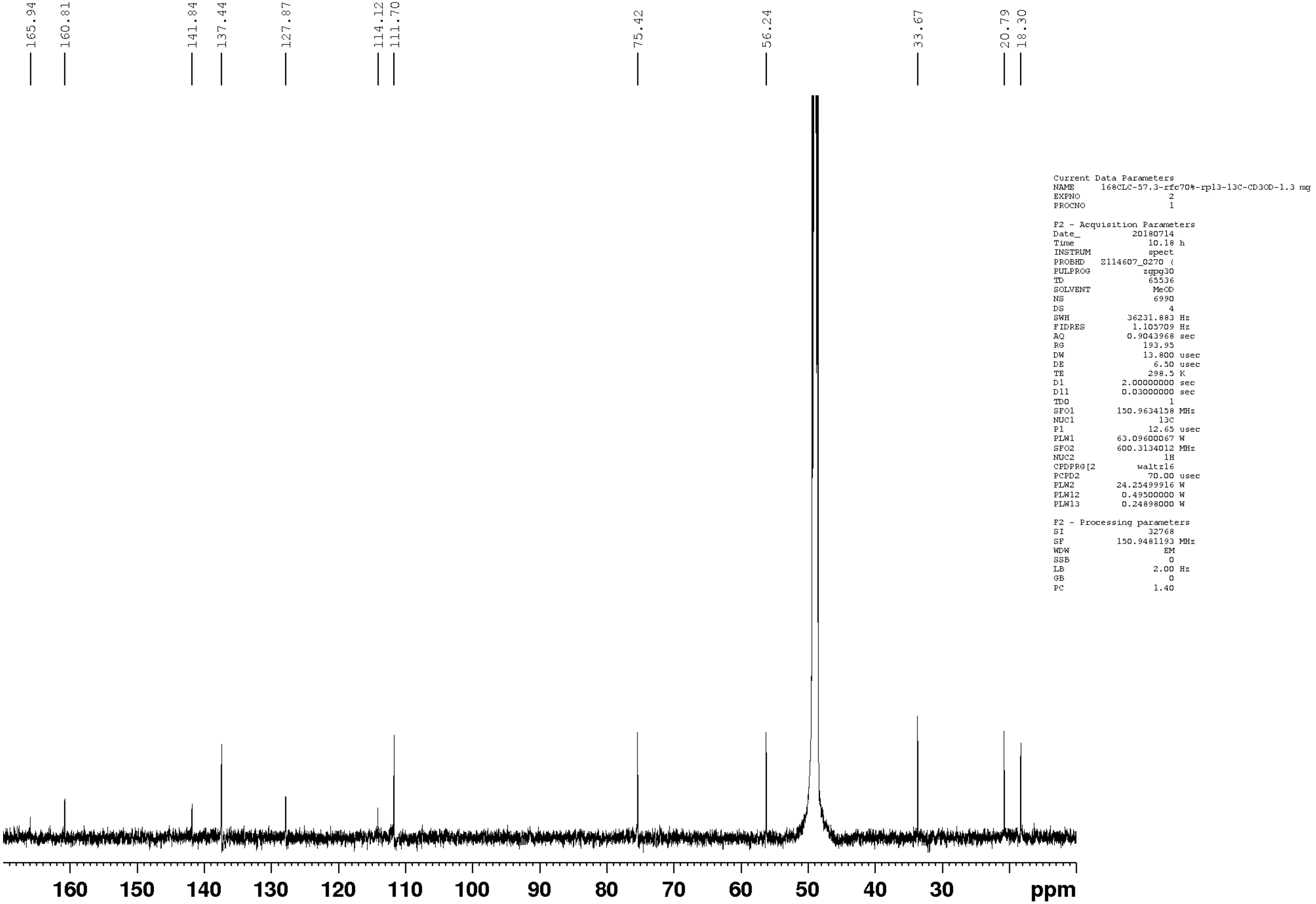

Figure S32. ^{1}H NMR spectrum of (–)-5-methylmellein (7)

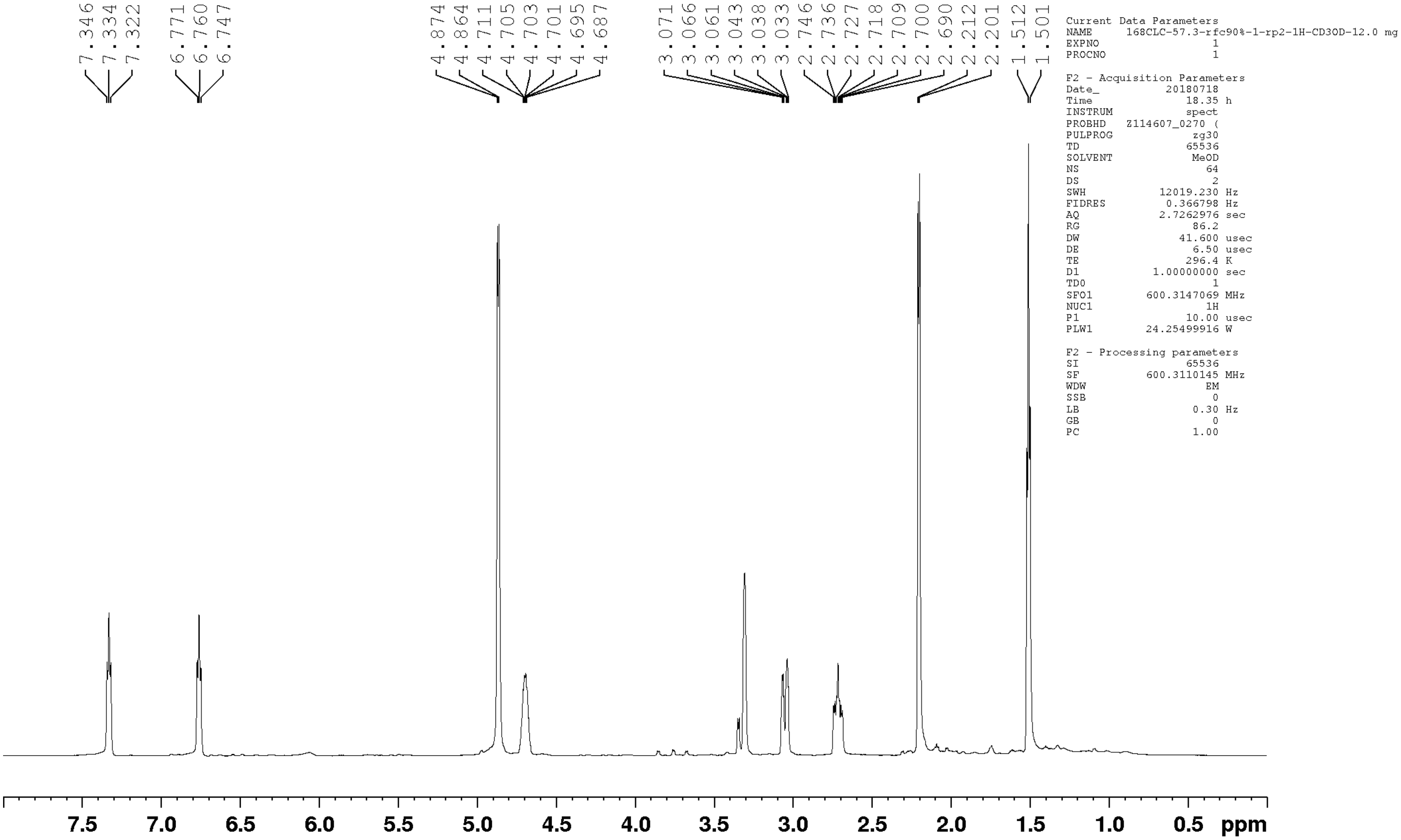

Figure S33. ^{13}C NMR spectrum of (–)-5-methylmellein (7)

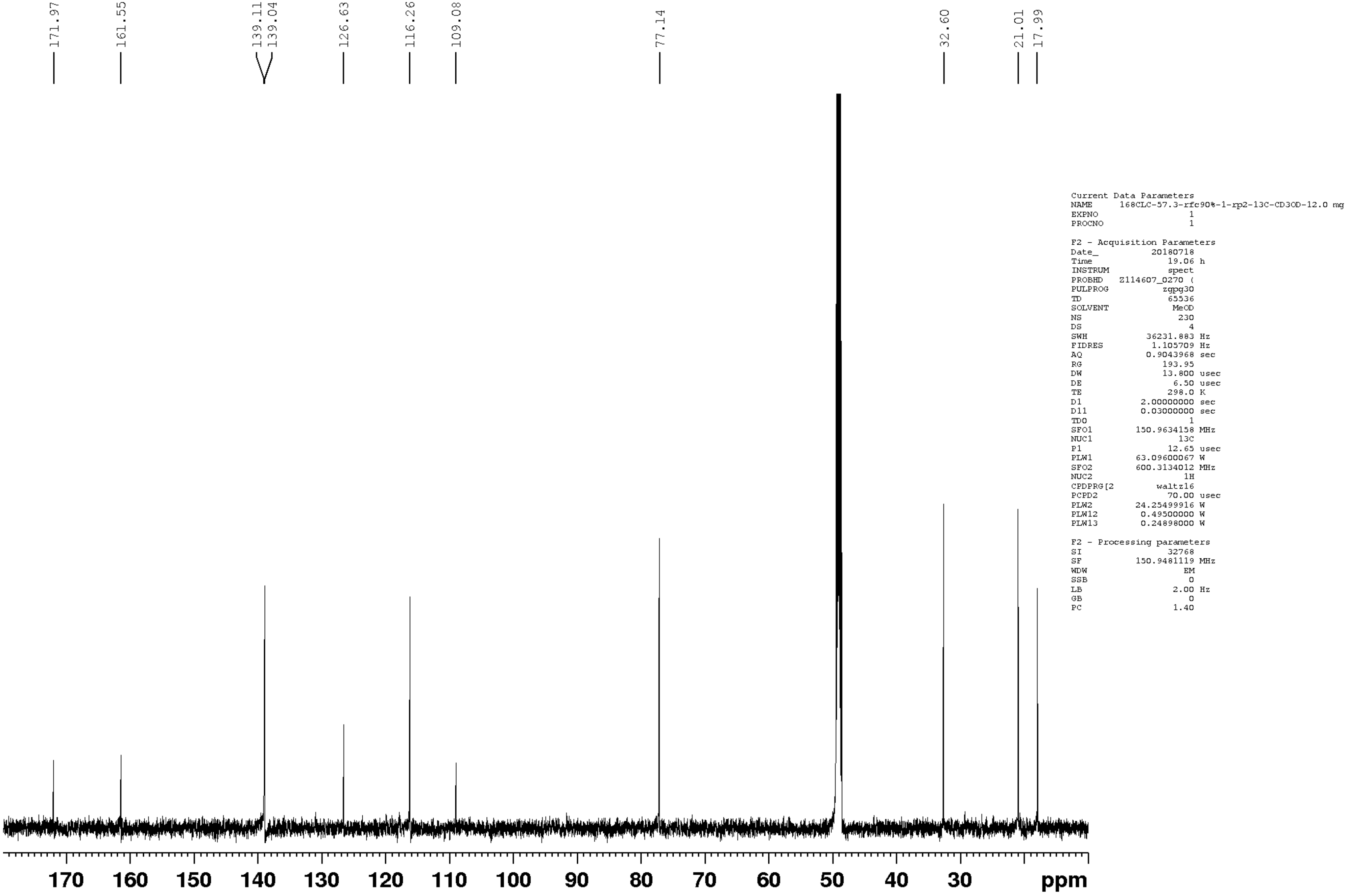

Figure S34. ^{1}**H NMR spectrum of dendrodochol B (8)**

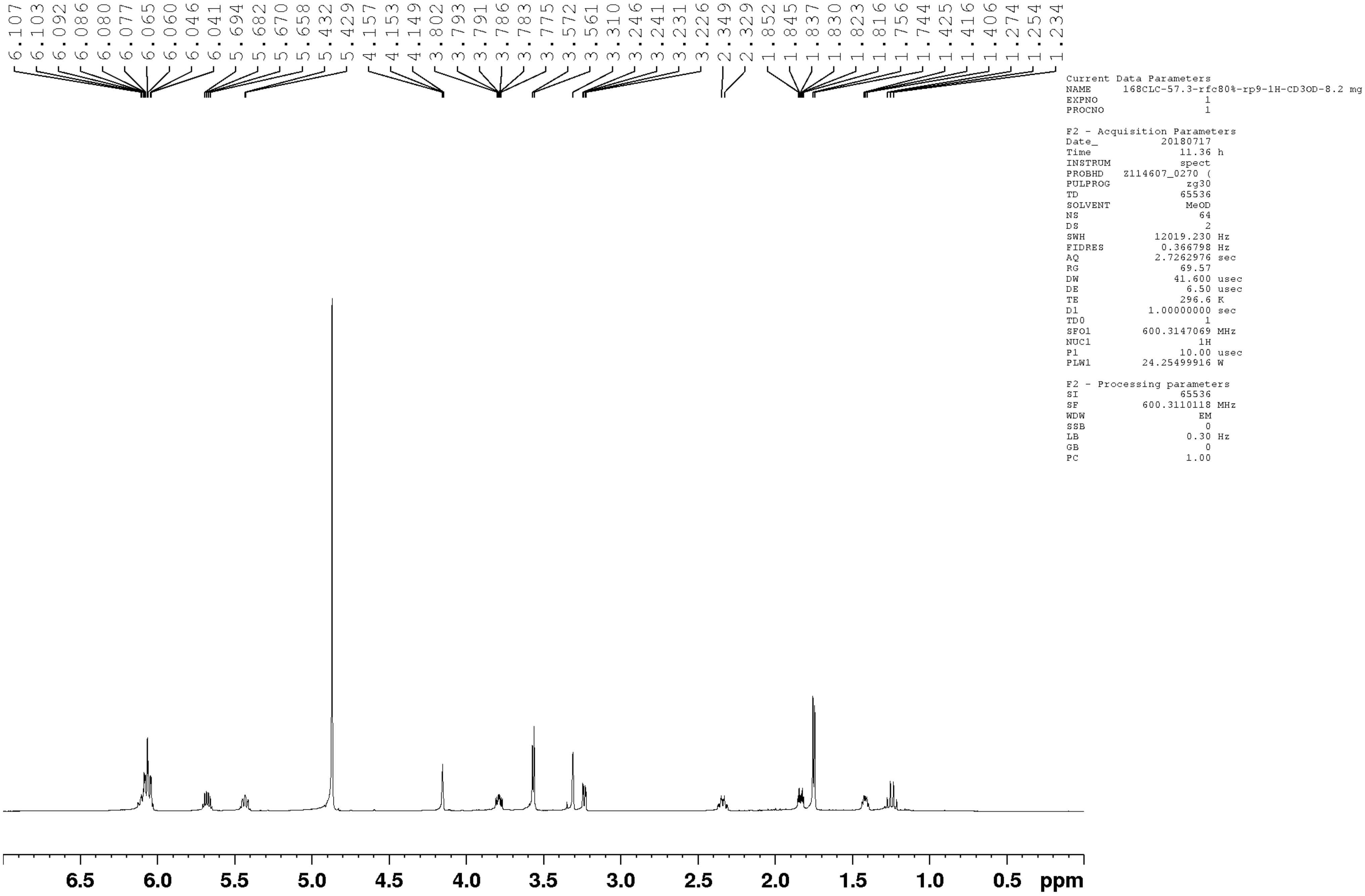

Figure S35. ^{13}C NMR spectrum of dendrodochol B (8)

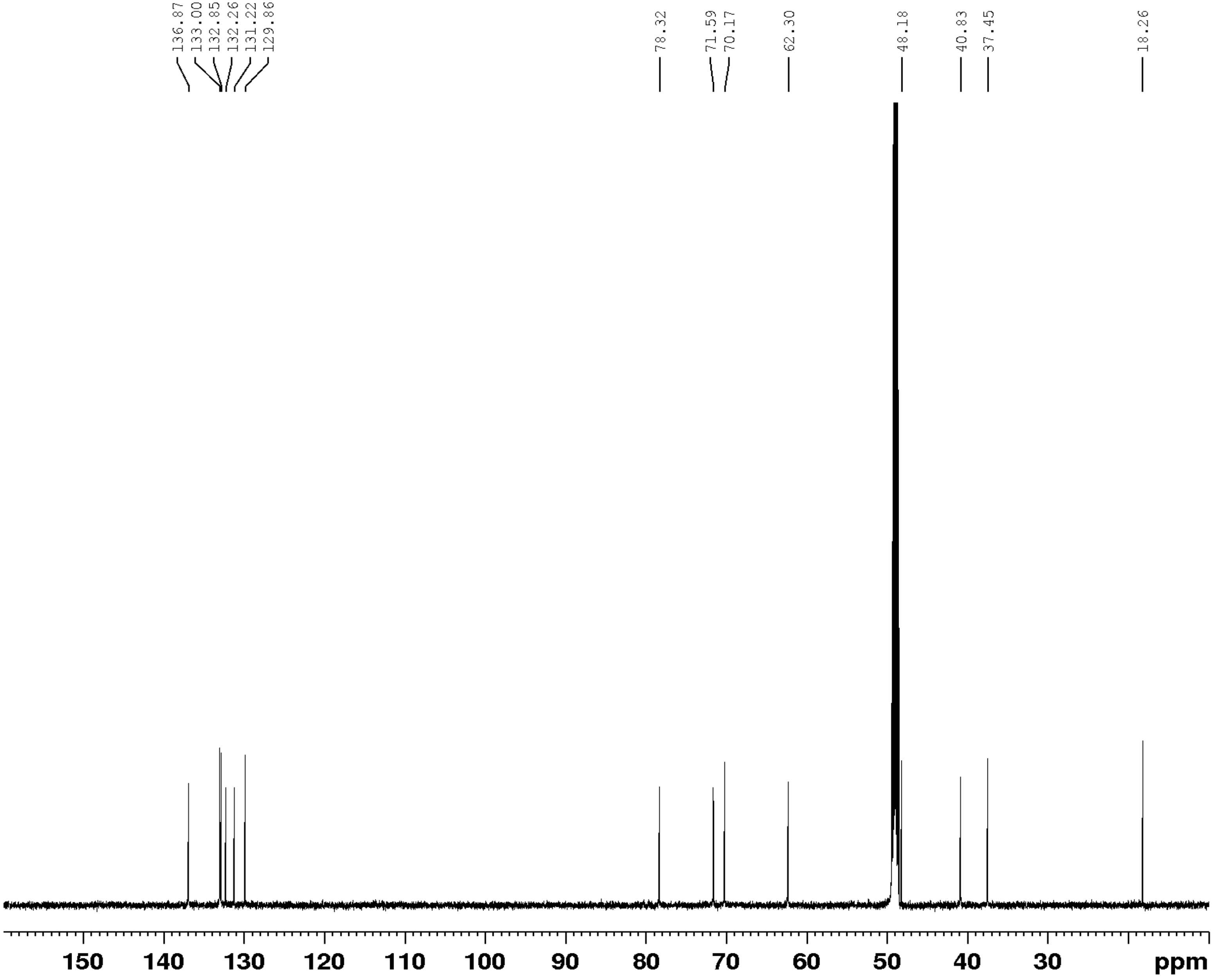


```
Current Data Parameters
NAME     168CLC-57.3-rfc80%-rp9-13c-CD3OD
EXPNO                 2
PROCNO                1

F2 - Acquisition Parameters
Date_          20180718
Time              13.41 h
INSTRUM           spect
PROBHD   Z114607_0270 (
PULPROG          zgpg30
TD                65536
SOLVENT            MeOD
NS                  532
DS                    4
SWH           36231.883 Hz
FIDRES         1.105709 Hz
AQ            0.9043968 sec
RG               193.95
DW               13.800 usec
DE                 6.50 usec
TE                298.0 K
D1           2.00000000 sec
D11          0.03000000 sec
TD0                   1
SFO1        150.9634158 MHz
NUC1                13C
P1                12.65 usec
PLW1        63.09600067 W
SFO2        600.3134012 MHz
NUC2                 1H
CPDPRG[2        waltz16
PCPD2             70.00 usec
PLW2        24.25499916 W
PLW12        0.49500000 W
PLW13        0.24898000 W

F2 - Processing parameters
SI                32768
SF          150.9481236 MHz
WDW                  EM
SSB                   0
LB                 1.00 Hz
GB                    0
PC                 1.40
```

Figure S36. ^{1}H **NMR spectrum of 1-O-acetyldendrodochol B (9)**

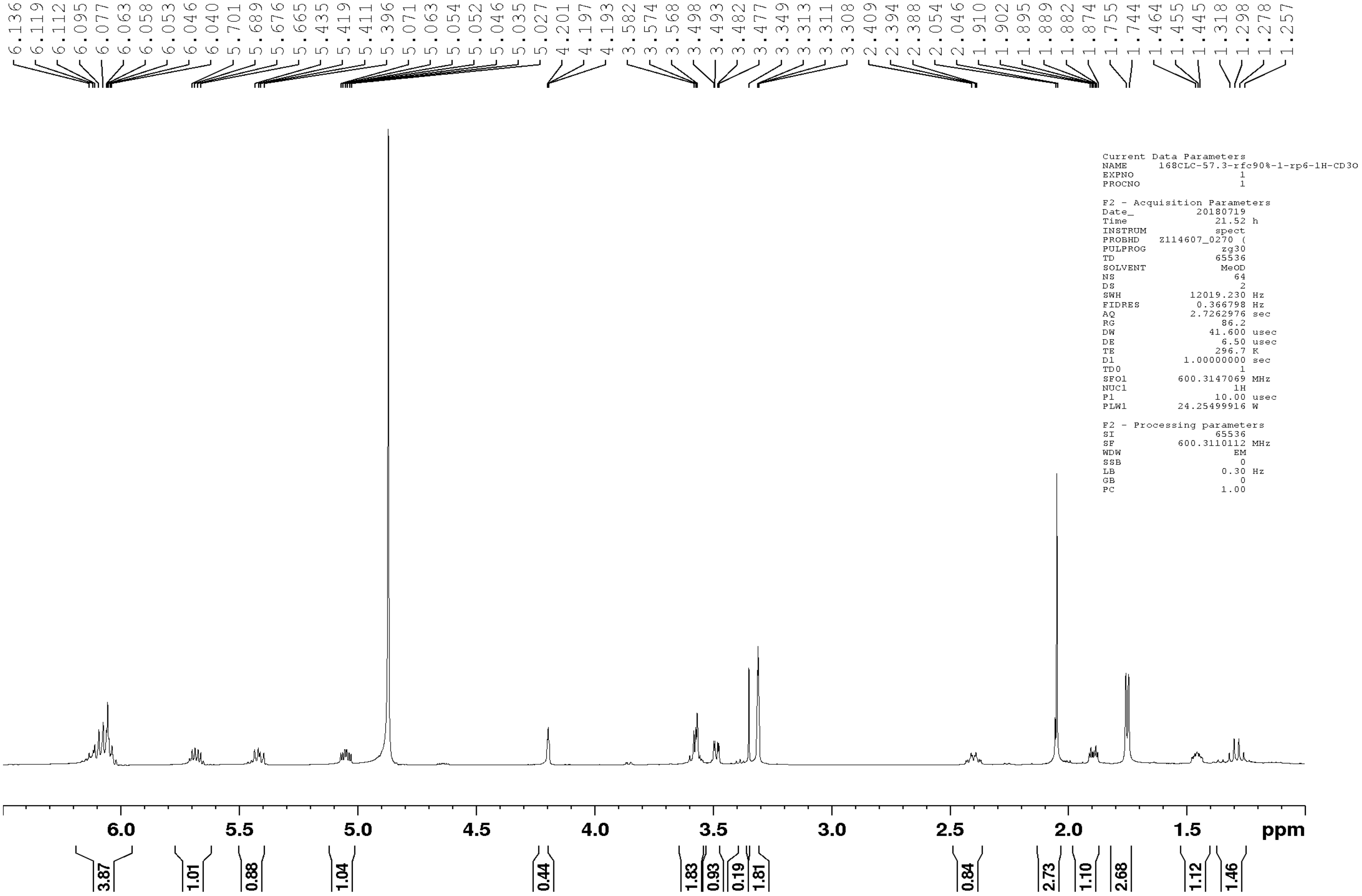

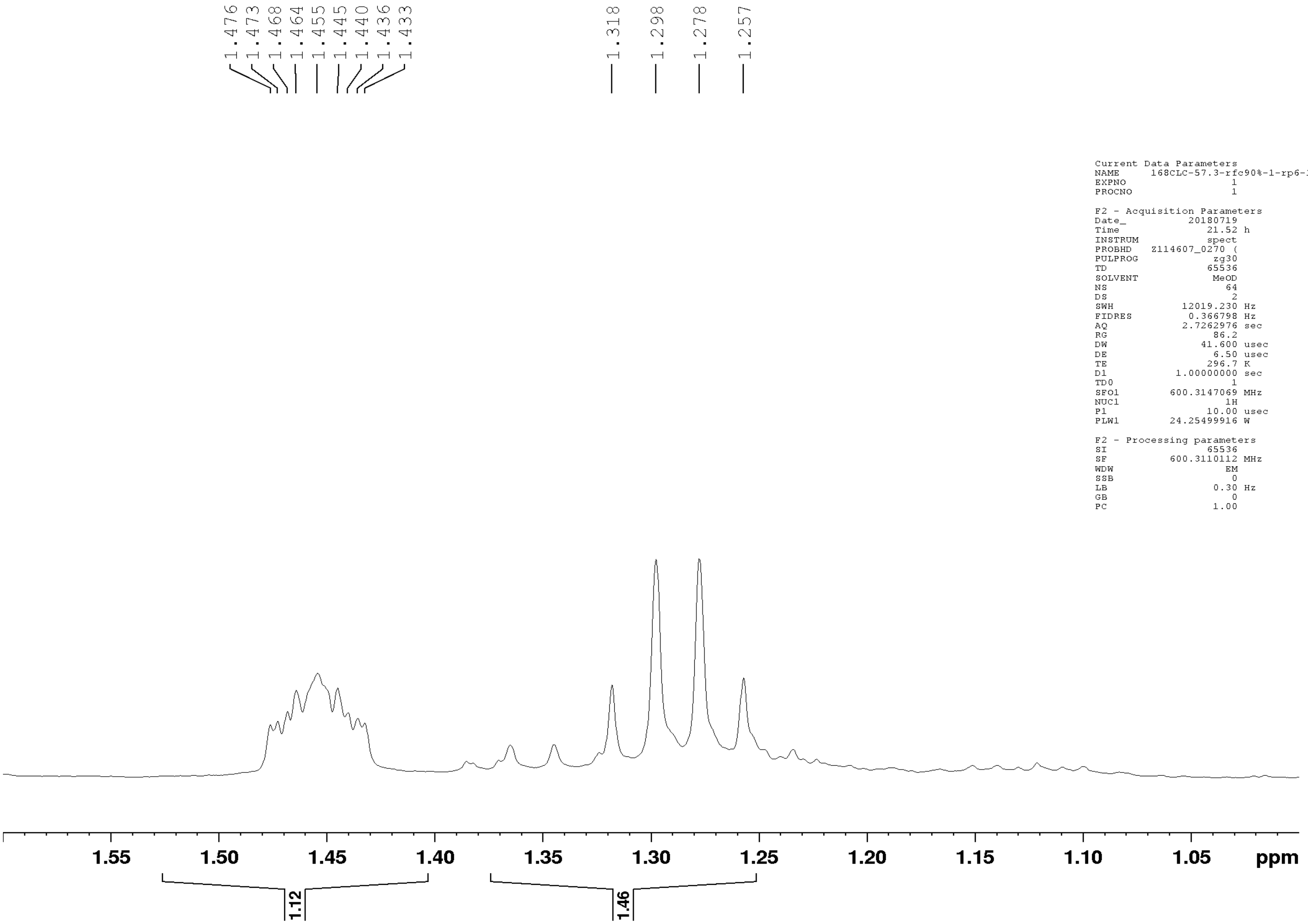

Current Data Parameters
NAME 168CLC-57.3-rfc90%-1-rp6-1H-CD3O
EXPNO 1
PROCNO 1

F2 - Acquisition Parameters
Date_ 20180719
Time 21.52 h
INSTRUM spect
PROBHD Z114607_0270 (
PULPROG zg30
TD 65536
SOLVENT MeOD
NS 64
DS 2
SWH 12019.230 Hz
FIDRES 0.366798 Hz
AQ 2.7262976 sec
RG 86.2
DW 41.600 usec
DE 6.50 usec
TE 296.7 K
D1 1.00000000 sec
TD0 1
SFO1 600.3147069 MHz
NUC1 1H
P1 10.00 usec
PLW1 24.25499916 W

F2 - Processing parameters
SI 65536
SF 600.3110112 MHz
WDW EM
SSB 0
LB 0.30 Hz
GB 0
PC 1.00

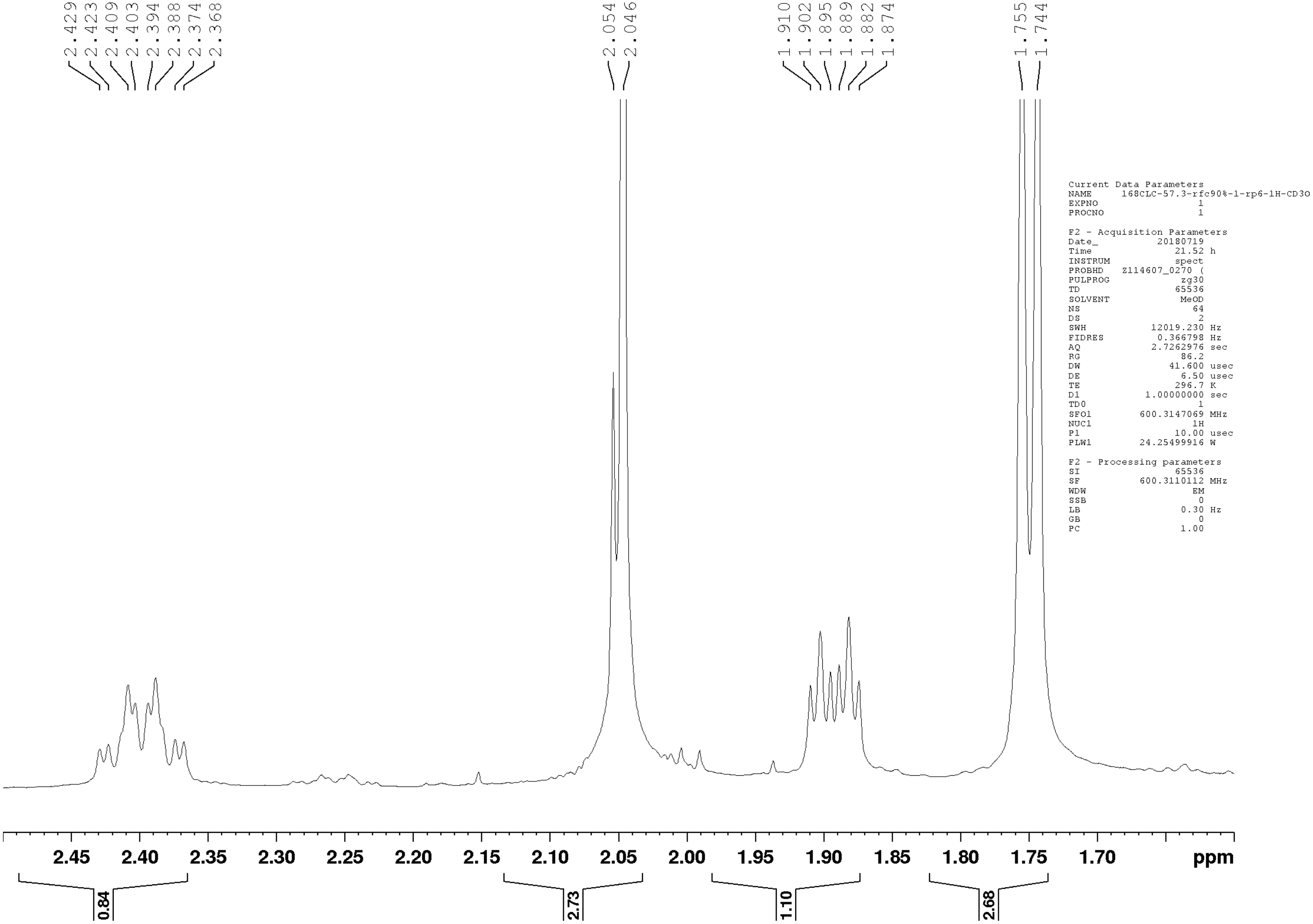
2.429
2.423
2.409
2.403
2.394
2.388
2.374
2.368
2.054
2.046
1.910
1.902
1.895
1.889
1.882
1.874
1.755
1.744
Current Data Parameters
NAME 168CLC-57.3-rfc90%-1-rp6-1H-CD3O
EXPNO 1
PROCNO 1
F2 - Acquisition Parameters
Date_ 20180719
Time 21.52 h
INSTRUM spect
PROBHD Z114607_0270 (
PULPROG zg30
TD 65536
SOLVENT MeOD
NS 64
DS 2
SWH 12019.230 Hz
FIDRES 0.366798 Hz
AQ 2.7262976 sec
RG 86.2
DW 41.600 usec
DE 6.50 usec
TE 296.7 K
D1 1.00000000 sec
TD0 1
SFO1 600.3147069 MHz
NUC1 1H
P1 10.00 usec
PLW1 24.25499916 W
F2 - Processing parameters
SI 65536
SF 600.3110112 MHz
WDW EM
SSB 0
LB 0.30 Hz
GB 0
PC 1.00
2.45
2.40
2.35
2.30
2.25
2.20
2.15
2.10
2.05
2.00
1.95
1.90
1.85
1.80
1.75
1.70
ppm
0.84
2.73
1.10
2.68

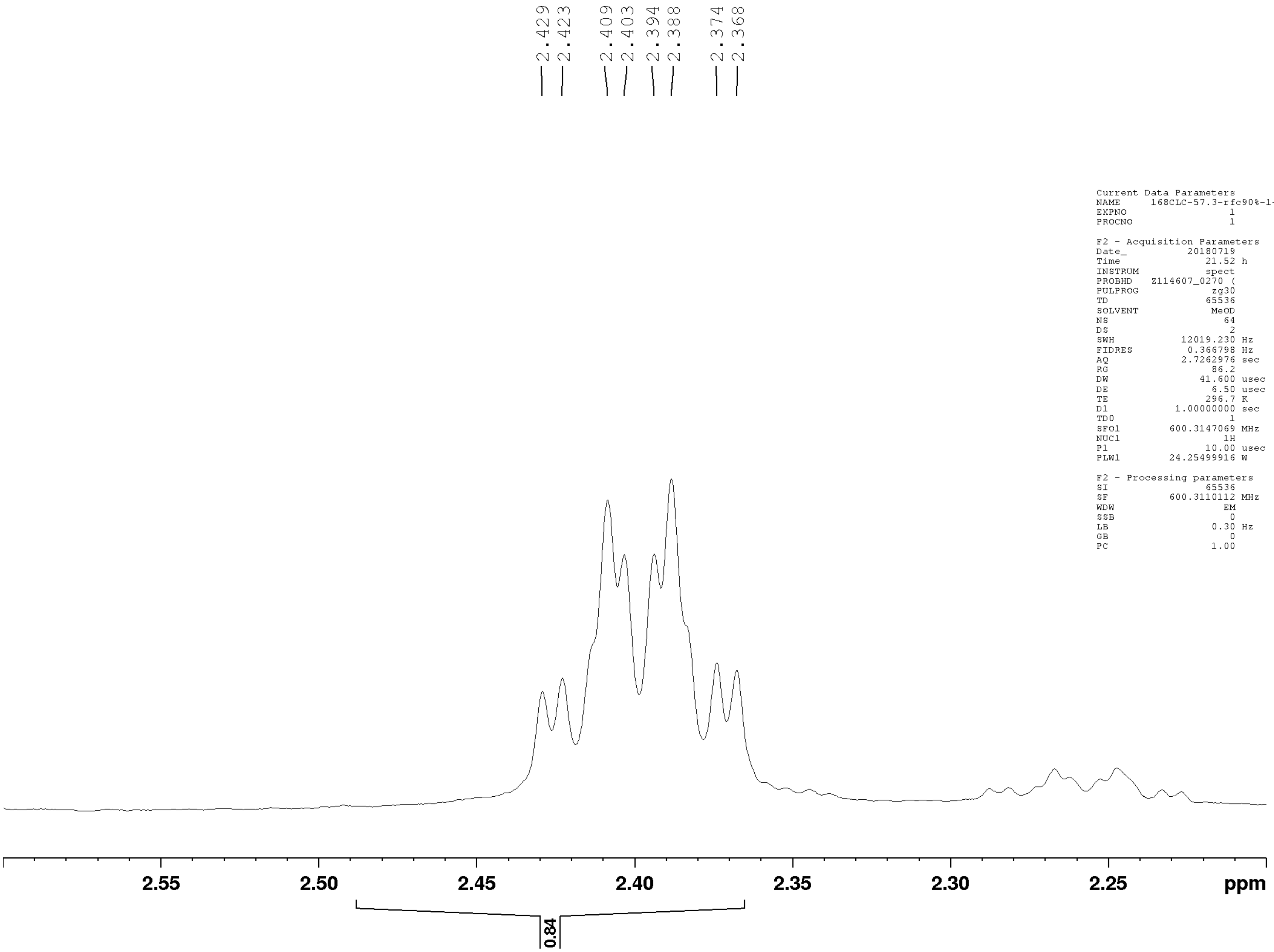

Current Data Parameters
NAME 168CLC-57.3-rfc90%-1-rp6-1H-CD3O
EXPNO 1
PROCNO 1

F2 - Acquisition Parameters
Date_ 20180719
Time 21.52 h
INSTRUM spect
PROBHD Z114607_0270 (
PULPROG zg30
TD 65536
SOLVENT MeOD
NS 64
DS 2
SWH 12019.230 Hz
FIDRES 0.366798 Hz
AQ 2.7262976 sec
RG 86.2
DW 41.600 usec
DE 6.50 usec
TE 296.7 K
D1 1.00000000 sec
TD0 1
SFO1 600.3147069 MHz
NUC1 1H
P1 10.00 usec
PLW1 24.25499916 W

F2 - Processing parameters
SI 65536
SF 600.3110112 MHz
WDW EM
SSB 0
LB 0.30 Hz
GB 0
PC 1.00

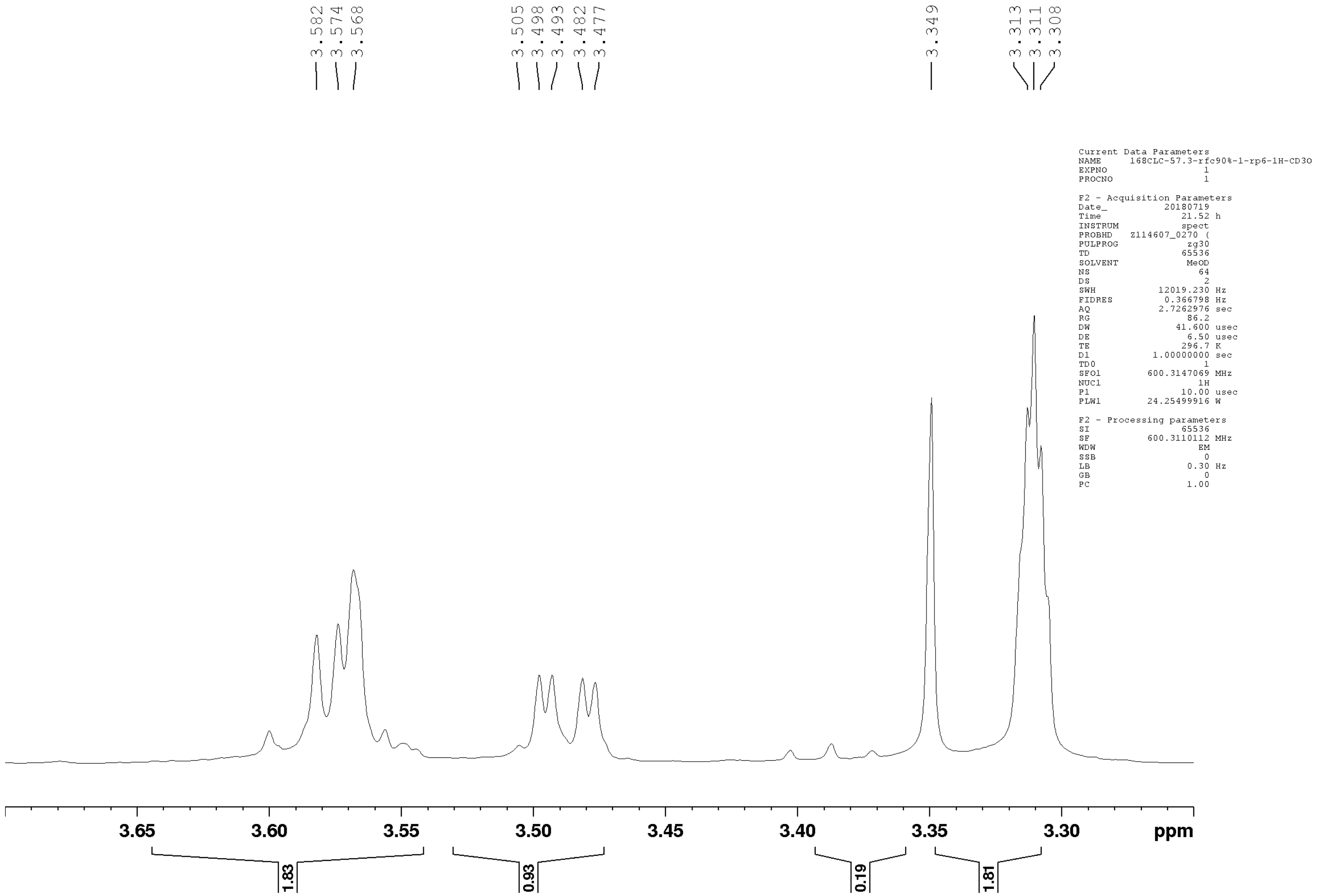
3.582
3.574
3.568
3.505
3.498
3.493
3.482
3.477
3.349
3.313
3.311
3.308
Current Data Parameters
NAME 168CLC-57.3-rfc90%-1-rp6-1H-CD3O
EXPNO 1
PROCNO 1
F2 - Acquisition Parameters
Date_ 20180719
Time 21.52 h
INSTRUM spect
PROBHD Z114607_0270 (
PULPROG zg30
TD 65536
SOLVENT MeOD
NS 64
DS 2
SWH 12019.230 Hz
FIDRES 0.366798 Hz
AQ 2.7262976 sec
RG 86.2
DW 41.600 usec
DE 6.50 usec
TE 296.7 K
D1 1.00000000 sec
TD0 1
SFO1 600.3147069 MHz
NUC1 1H
P1 10.00 usec
PLW1 24.25499916 W
F2 - Processing parameters
SI 65536
SF 600.3110112 MHz
WDW EM
SSB 0
LB 0.30 Hz
GB 0
PC 1.00
3.65
3.60
3.55
3.50
3.45
3.40
3.35
3.30
ppm
1.83
0.93
0.19
1.81

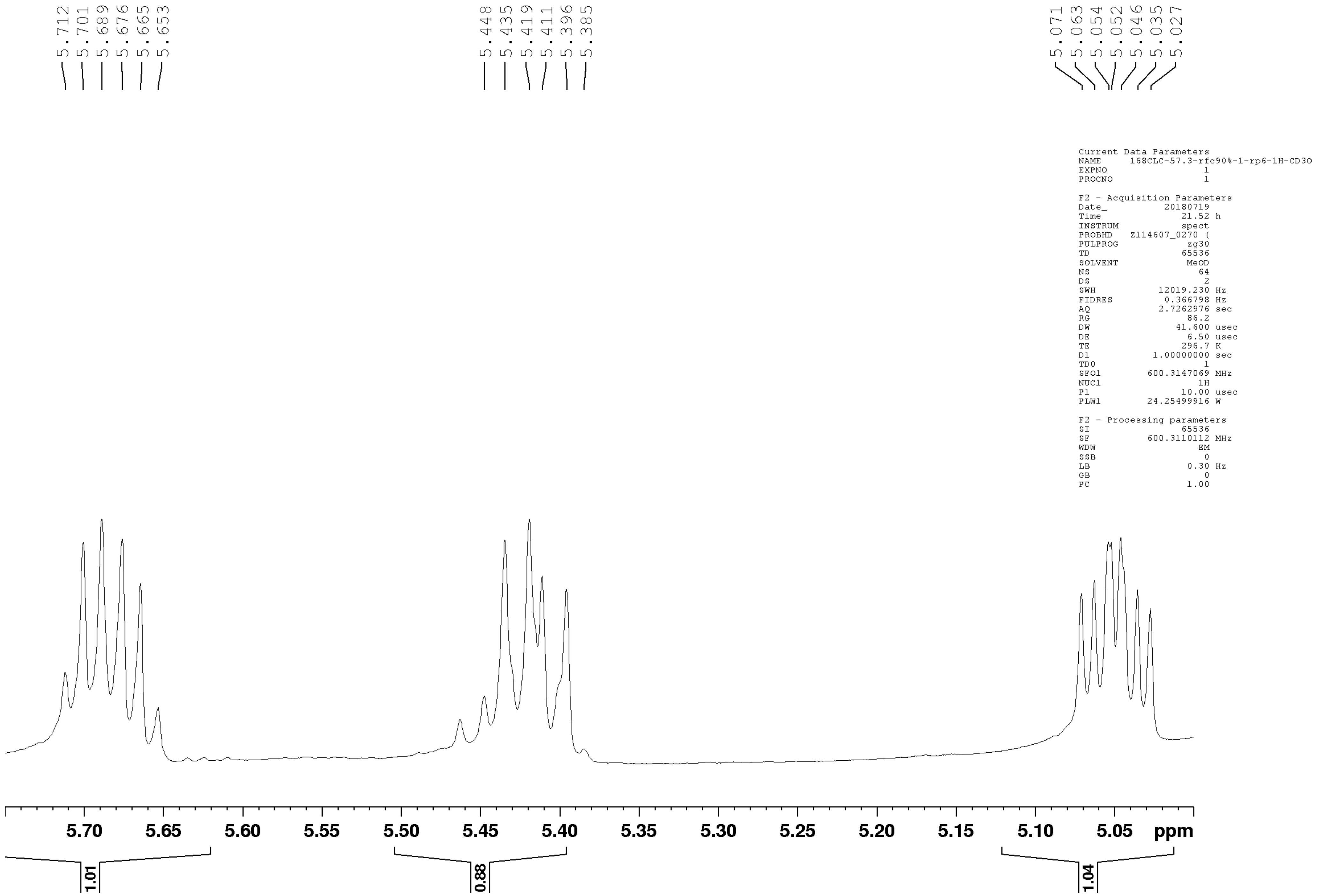

5.712
5.701
5.689
5.676
5.665
5.653
5.448
5.435
5.419
5.411
5.396
5.385
5.071
5.063
5.054
5.052
5.046
5.035
5.027
Current Data Parameters
NAME 168CLC-57.3-rfc90%-1-rp6-1H-CD3O
EXPNO 1
PROCNO 1
F2 - Acquisition Parameters
Date_ 20180719
Time 21.52 h
INSTRUM spect
PROBHD Z114607_0270 (
PULPROG zg30
TD 65536
SOLVENT MeOD
NS 64
DS 2
SWH 12019.230 Hz
FIDRES 0.366798 Hz
AQ 2.7262976 sec
RG 86.2
DW 41.600 usec
DE 6.50 usec
TE 296.7 K
D1 1.00000000 sec
TD0 1
SFO1 600.3147069 MHz
NUC1 1H
P1 10.00 usec
PLW1 24.25499916 W
F2 - Processing parameters
SI 65536
SF 600.3110112 MHz
WDW EM
SSB 0
LB 0.30 Hz
GB 0
PC 1.00
5.70
5.65
5.60
5.55
5.50
5.45
5.40
5.35
5.30
5.25
5.20
5.15
5.10
5.05
ppm
1.01
0.88
1.04

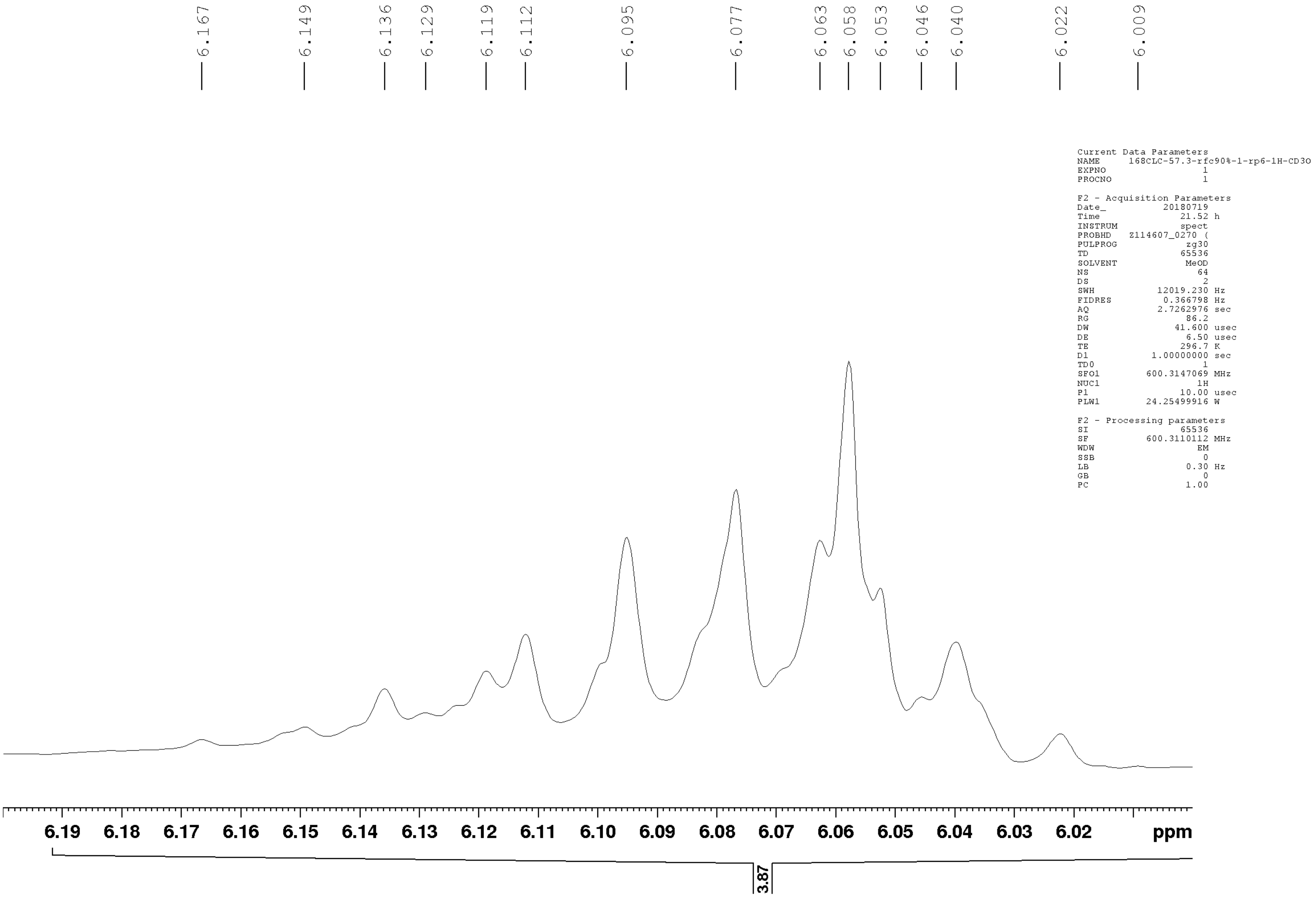
6.167
6.149
6.136
6.129
6.119
6.112
6.095
6.077
6.063
6.058
6.053
6.046
6.040
6.022
6.009
Current Data Parameters
NAME 168CLC-57.3-rfc90%-1-rp6-1H-CD3O
EXPNO 1
PROCNO 1
F2 - Acquisition Parameters
Date_ 20180719
Time 21.52 h
INSTRUM spect
PROBHD Z114607_0270 (
PULPROG zg30
TD 65536
SOLVENT MeOD
NS 64
DS 2
SWH 12019.230 Hz
FIDRES 0.366798 Hz
AQ 2.7262976 sec
RG 86.2
DW 41.600 usec
DE 6.50 usec
TE 296.7 K
D1 1.00000000 sec
TD0 1
SFO1 600.3147069 MHz
NUC1 1H
P1 10.00 usec
PLW1 24.25499916 W
F2 - Processing parameters
SI 65536
SF 600.3110112 MHz
WDW EM
SSB 0
LB 0.30 Hz
GB 0
PC 1.00
6.19 6.18 6.17 6.16 6.15 6.14 6.13 6.12 6.11 6.10 6.09 6.08 6.07 6.06 6.05 6.04 6.03 6.02 ppm
3.87

Figure S37. ^{13}C NMR spectrum of 1-O-acetyldendrodochol B (9)

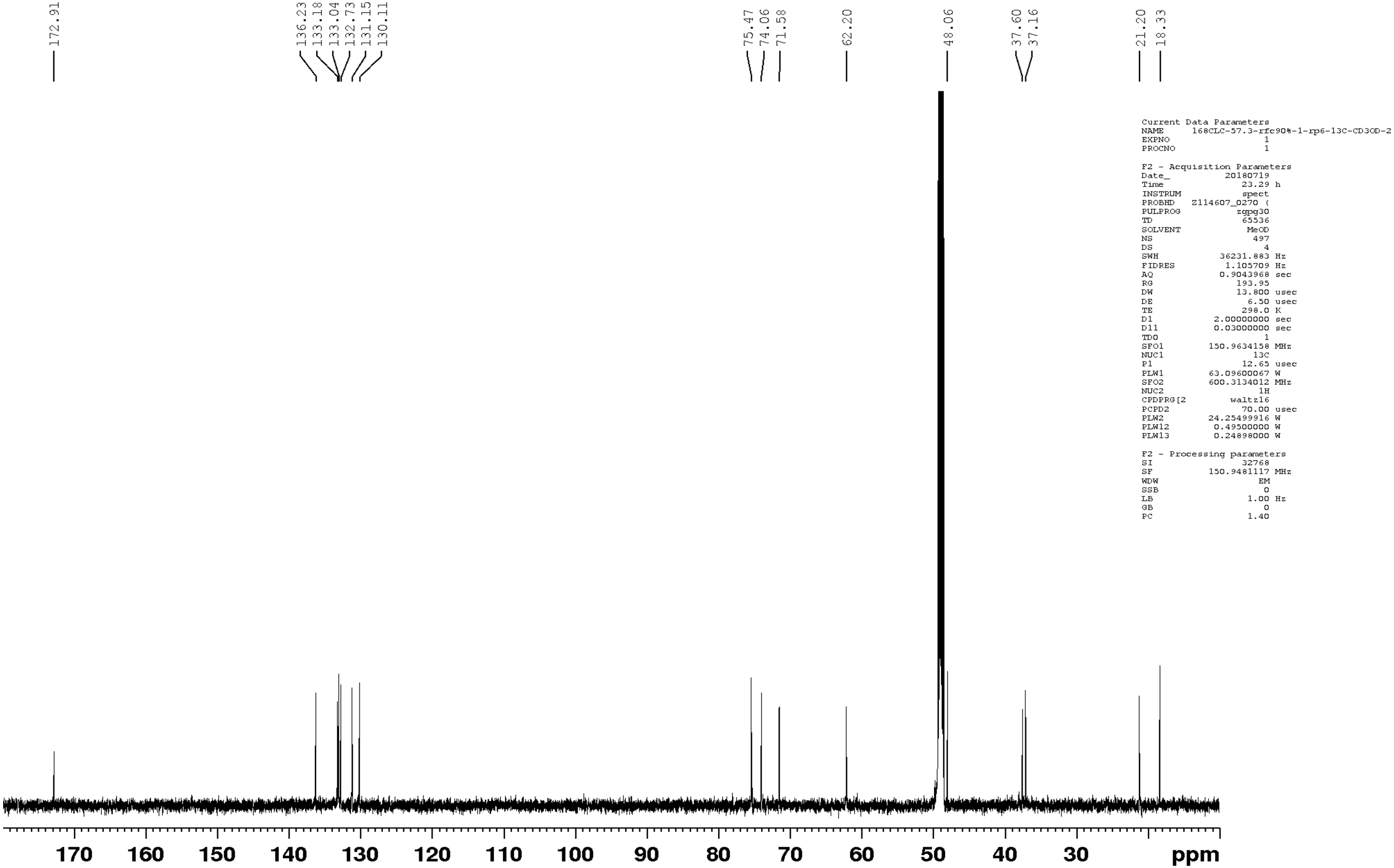

Figure S38. ¹H-¹ spectrum of 1-O-acetyldendrodochol B (9)

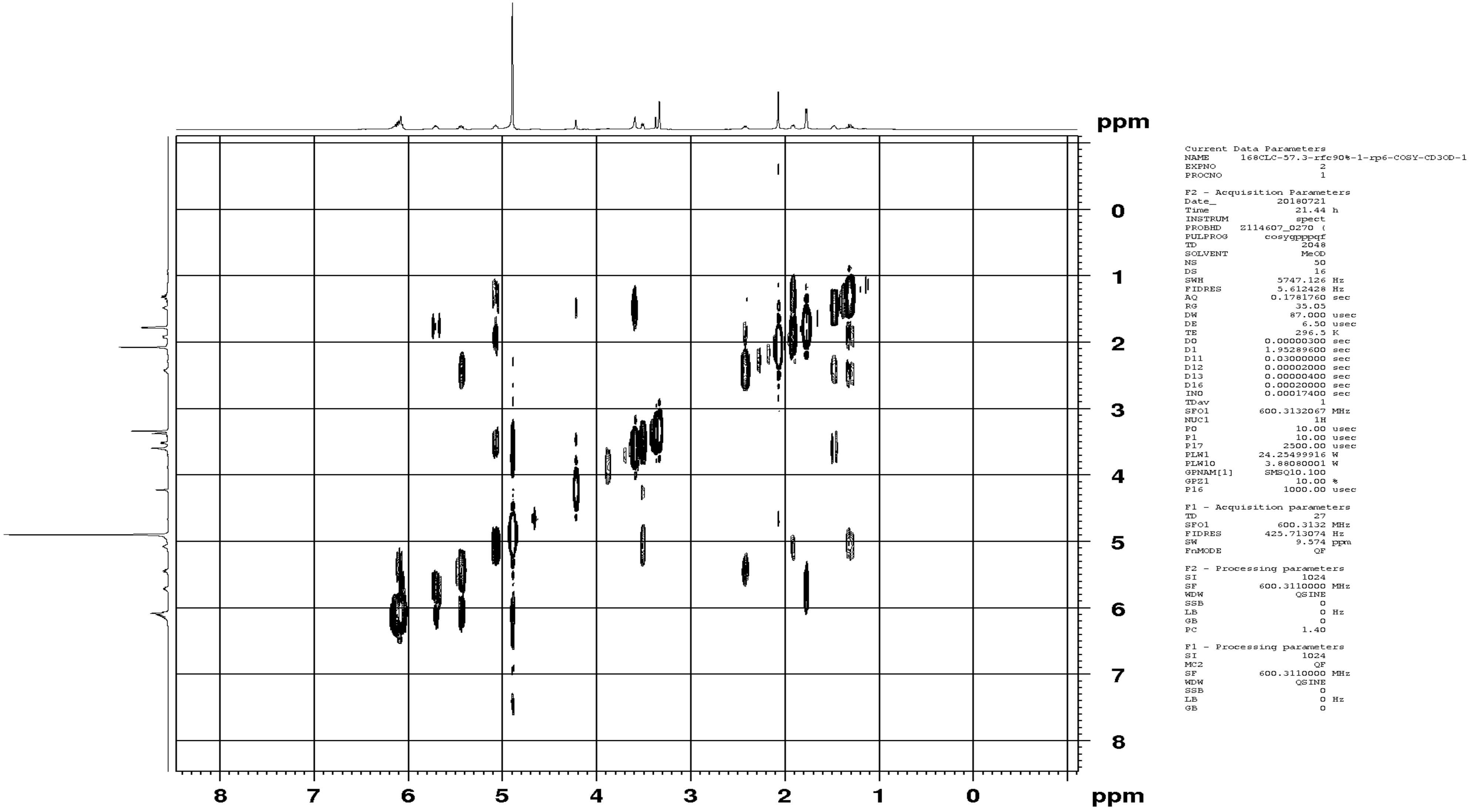

Figure S39. HMBC spectrum of 1-O-acetyldendrodochol B (9)

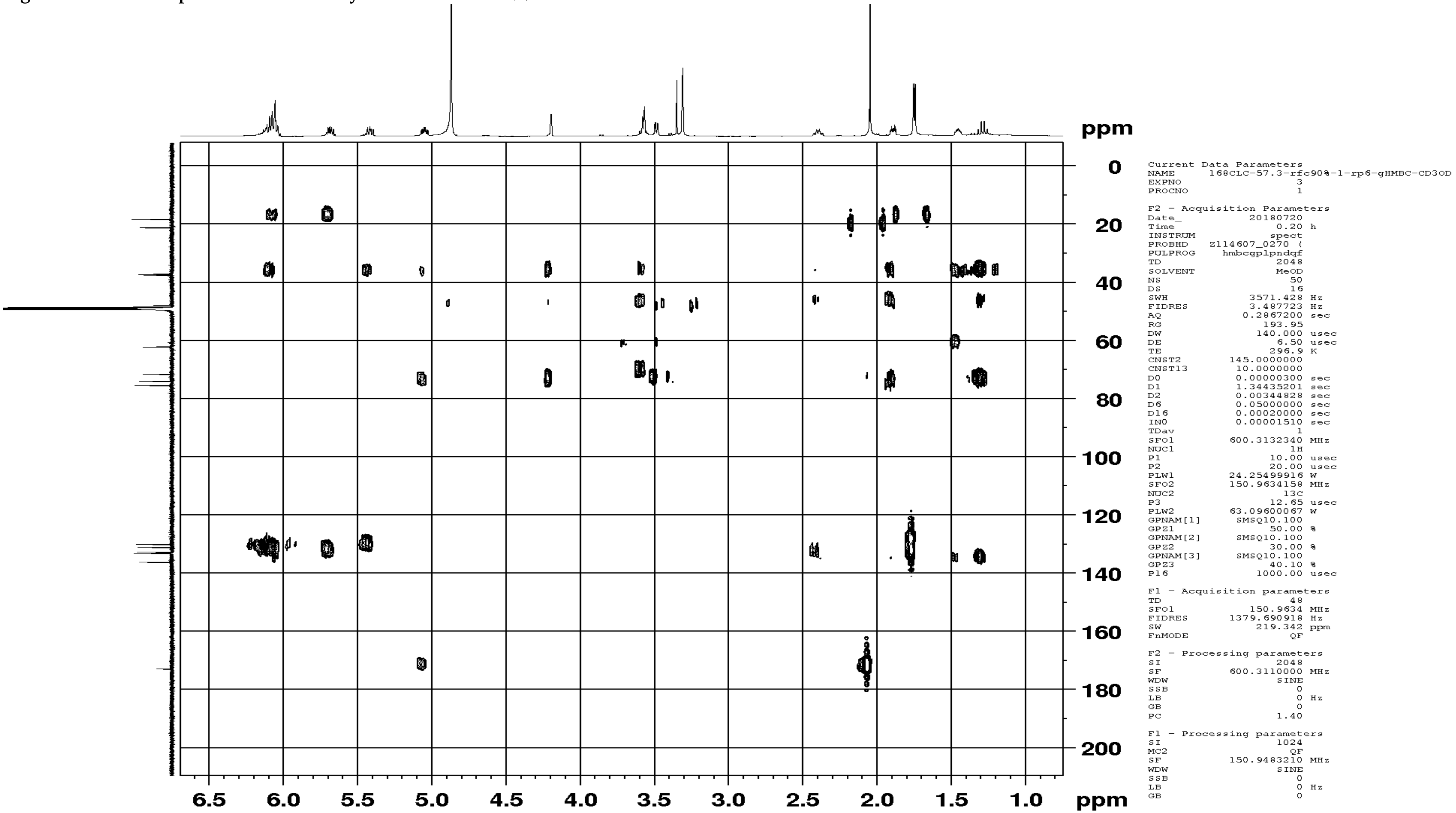

Figure S40. HSQC spectrum of 1-O-acetyldendrodochol B (9)

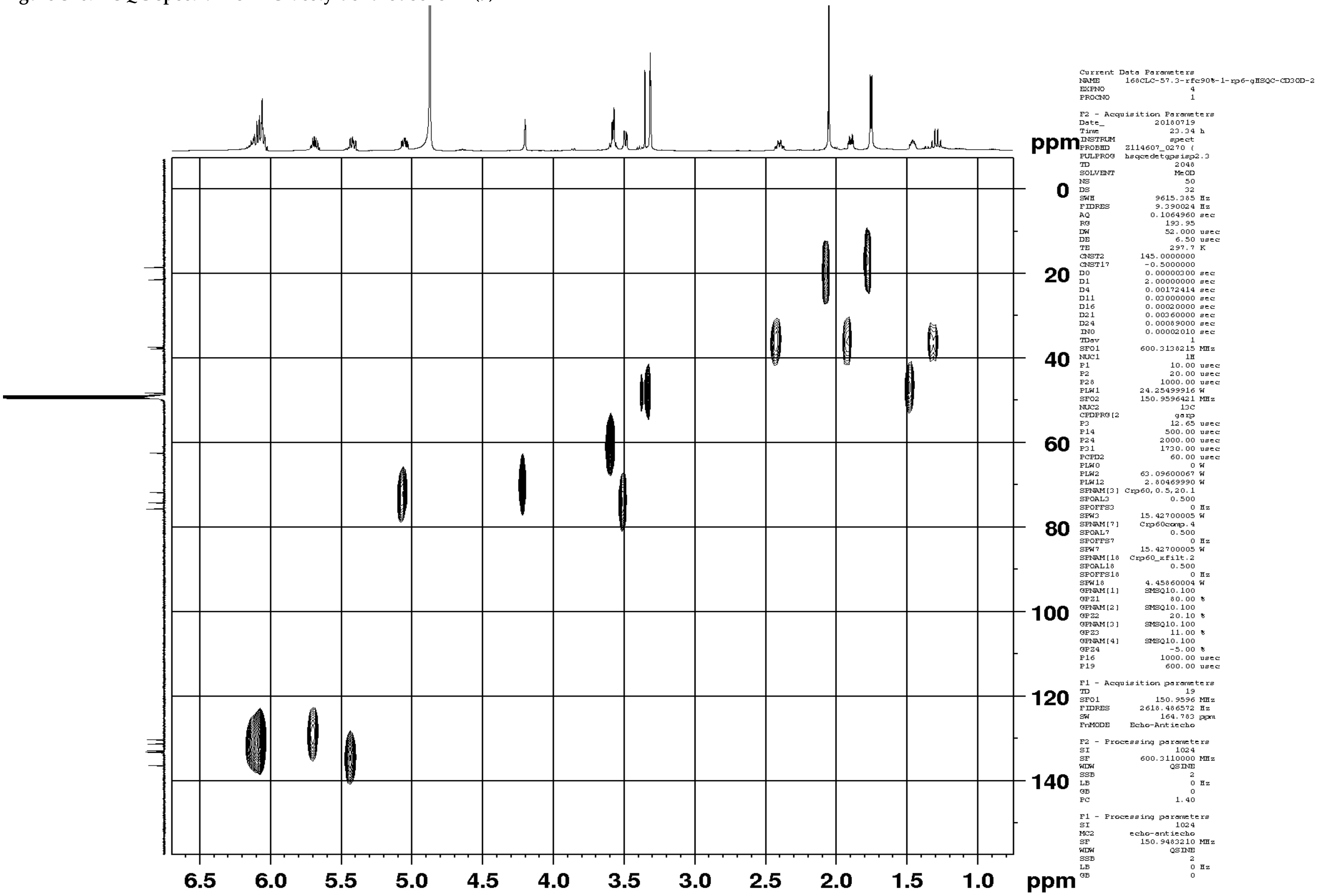

Figure S41. CD spectrum of 1-O-acetyldendrodochol B (9)

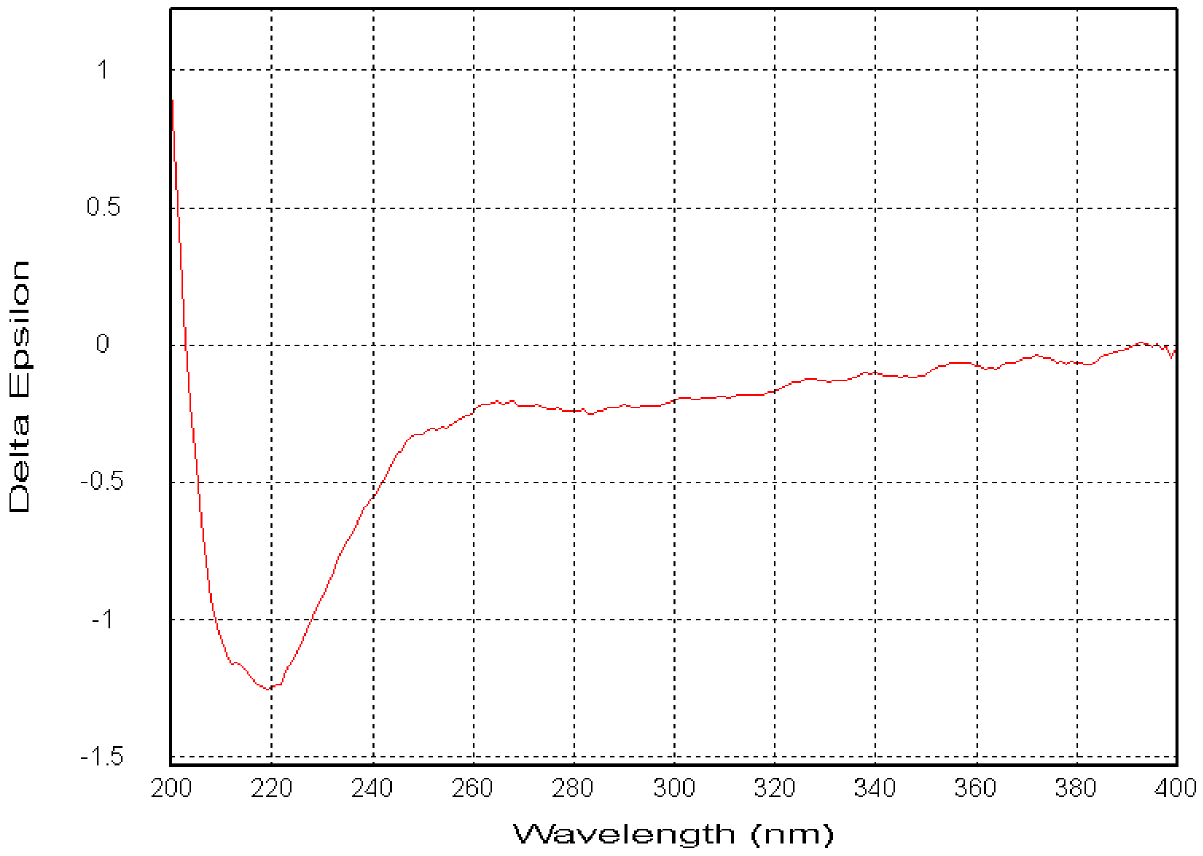

Figure S42. ^{1}**H NMR spectrum of 2-O-acetyldendrodochol B (10)**

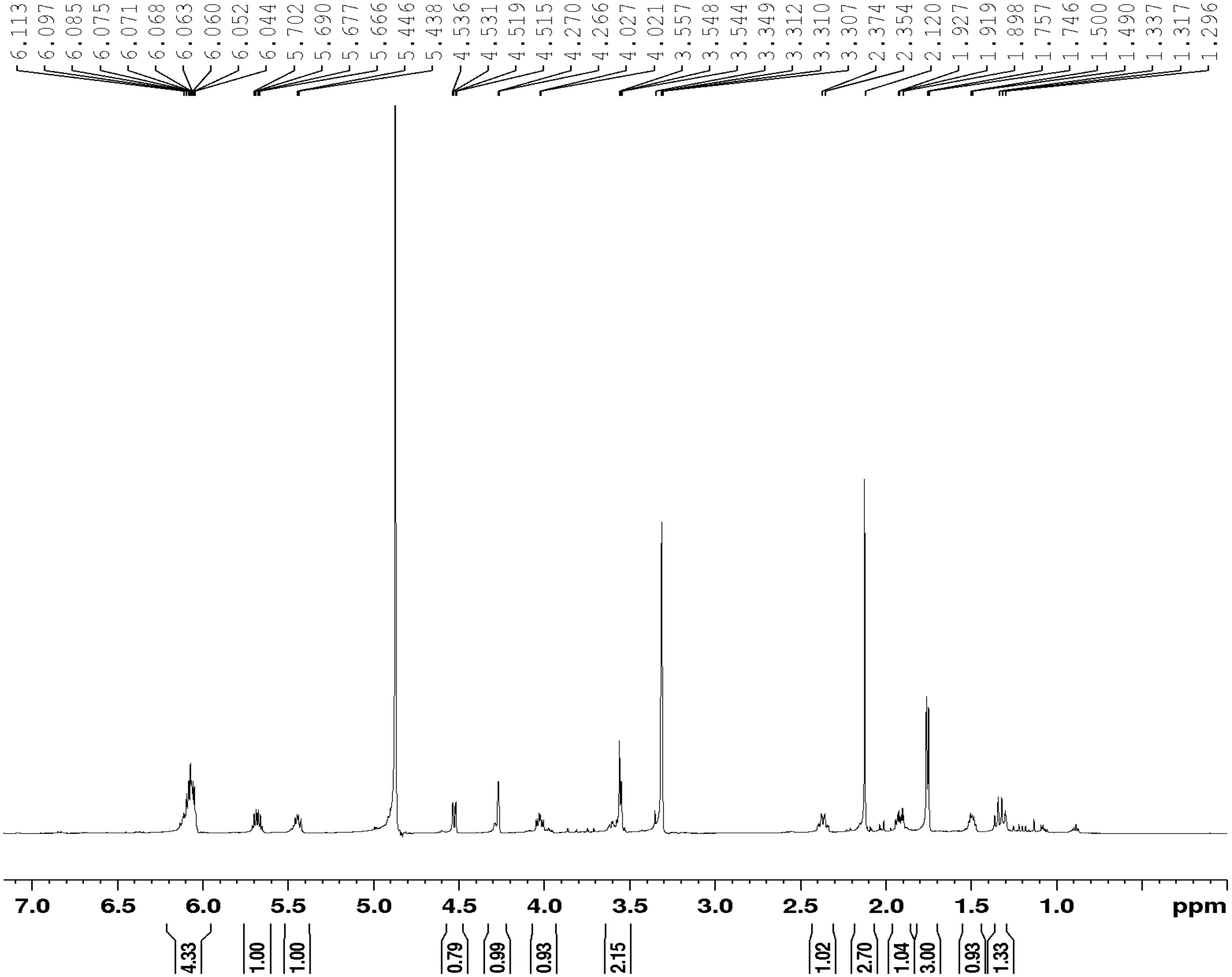


```
Current Data Parameters
NAME      168CLC-57.3-rfc80%-rp10-1H-CD3OD-2.9 mg
EXPNO                1
PROCNO               1

F2 - Acquisition Parameters
Date_          20180717
Time              11.43 h
INSTRUM           spect
PROBHD   Z114607_0270 (
PULPROG            zg30
TD                65536
SOLVENT            MeOD
NS                   64
DS                    2
SWH           12019.230 Hz
FIDRES         0.366798 Hz
AQ            2.7262976 sec
RG               109.62
DW               41.600 usec
DE                 6.50 usec
TE                296.5 K
D1           1.00000000 sec
TD0                   1
SFO1        600.3147069 MHz
NUC1                 1H
P1                10.00 usec
PLW1        24.25499916 W

F2 - Processing parameters
SI                65536
SF          600.3110118 MHz
WDW                  EM
SSB                   0
LB                 0.30 Hz
GB                    0
PC                 1.00
```

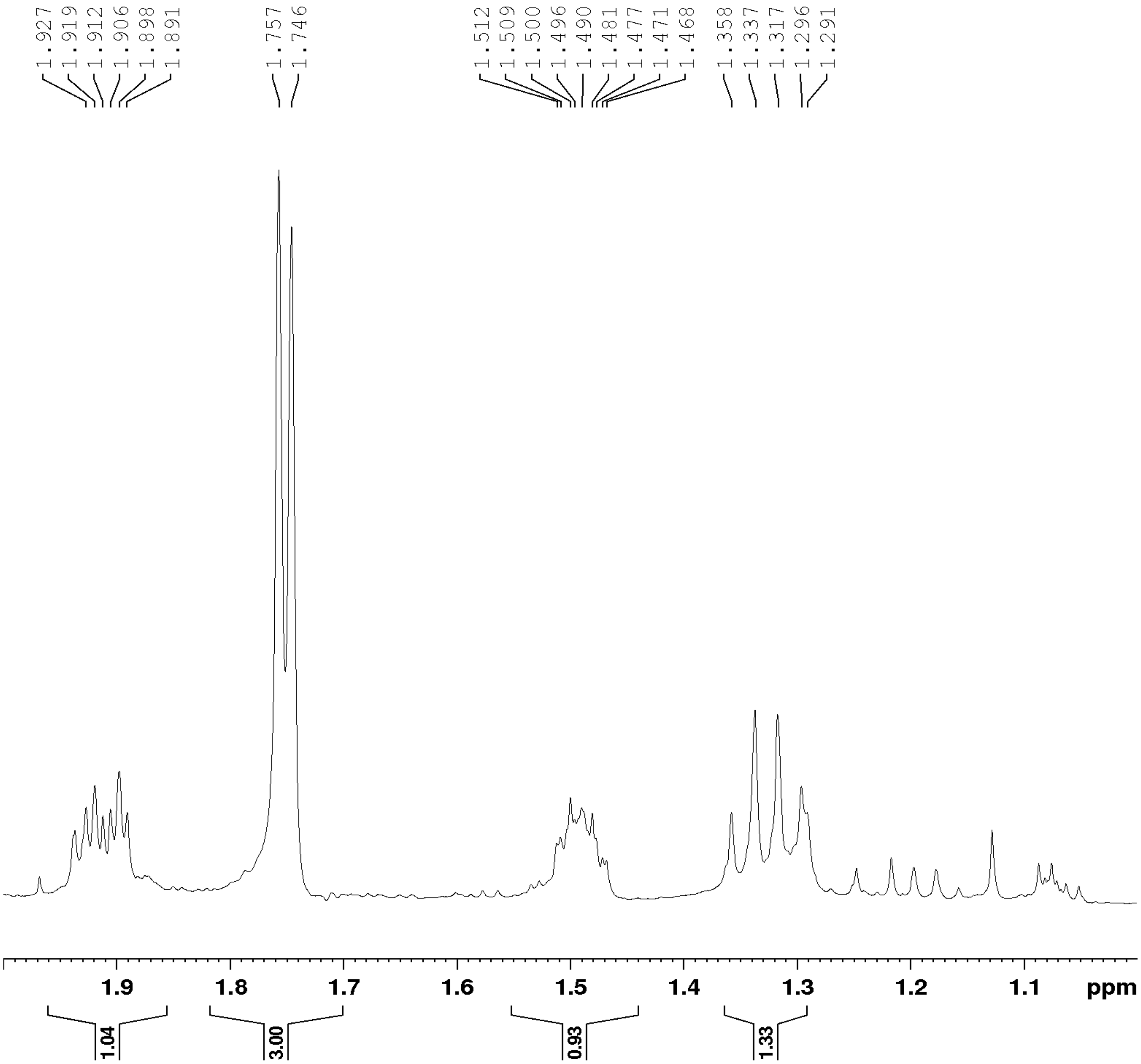


```
Current Data Parameters
NAME     168CLC-57.3-rfc80%-rp10-1H-CD3OD-2.9 mg
EXPNO                  1
PROCNO                 1

F2 - Acquisition Parameters
Date_           20180717
Time               11.43 h
INSTRUM            spect
PROBHD   Z114607_0270 (
PULPROG             zg30
TD                 65536
SOLVENT             MeOD
NS                    64
DS                     2
SWH            12019.230 Hz
FIDRES          0.366798 Hz
AQ             2.7262976 sec
RG                109.62
DW                41.600 usec
DE                  6.50 usec
TE                 296.5 K
D1            1.00000000 sec
TD0                    1
SFO1         600.3147069 MHz
NUC1                  1H
P1                 10.00 usec
PLW1         24.25499916 W

F2 - Processing parameters
SI                 65536
SF           600.3110118 MHz
WDW                   EM
SSB                    0
LB                  0.30 Hz
GB                     0
PC                  1.00
```

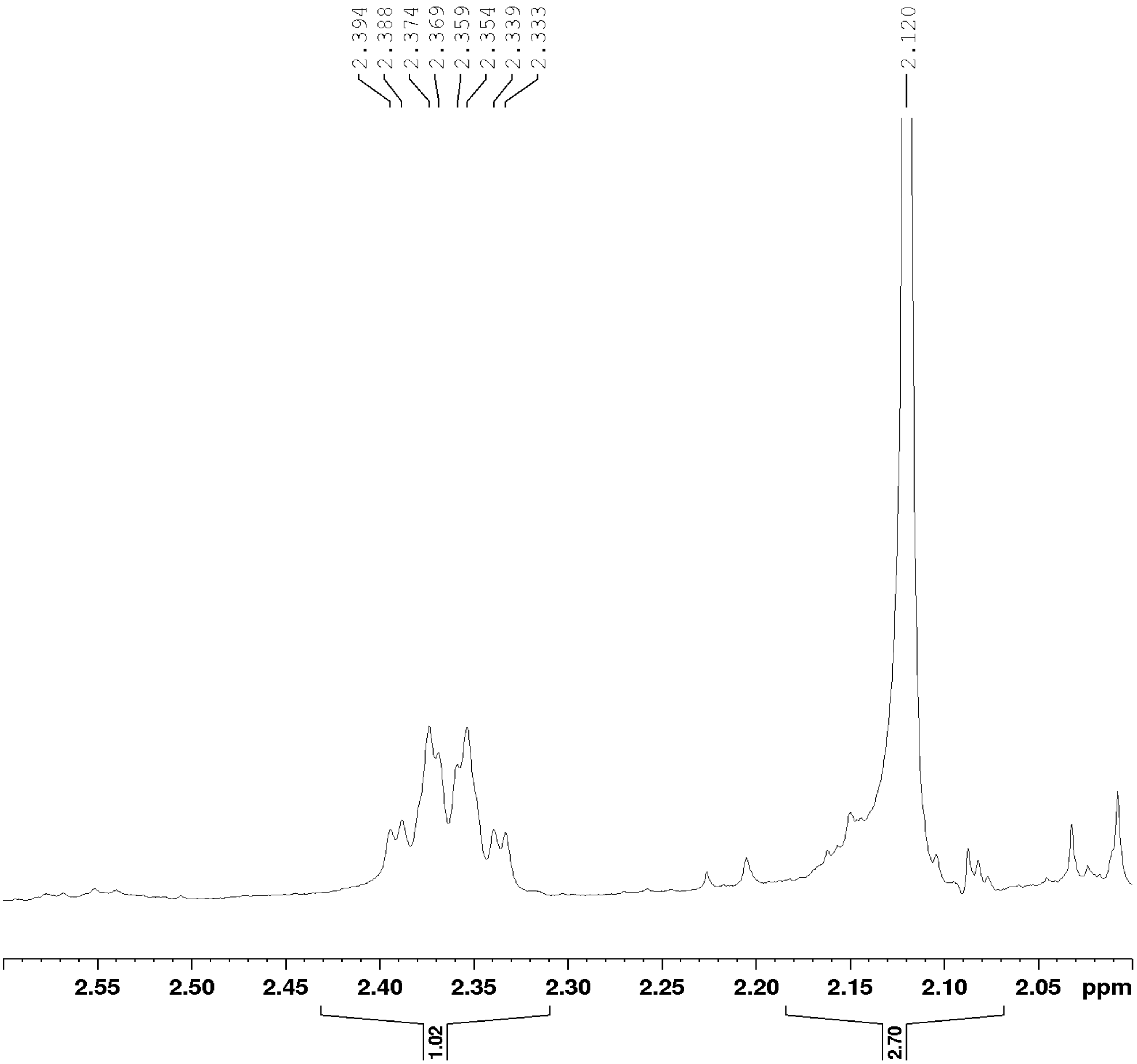


```
Current Data Parameters
NAME     168CLC-57.3-rfc80%-rp10-1H-CD3OD-2.9 mg
EXPNO                 1
PROCNO                1

F2 - Acquisition Parameters
Date_          20180717
Time              11.43 h
INSTRUM           spect
PROBHD   Z114607_0270 (
PULPROG            zg30
TD                65536
SOLVENT            MeOD
NS                   64
DS                    2
SWH           12019.230 Hz
FIDRES         0.366798 Hz
AQ            2.7262976 sec
RG               109.62
DW               41.600 usec
DE                 6.50 usec
TE                296.5 K
D1           1.00000000 sec
TD0                   1
SFO1        600.3147069 MHz
NUC1                 1H
P1                10.00 usec
PLW1        24.25499916 W

F2 - Processing parameters
SI                65536
SF          600.3110118 MHz
WDW                  EM
SSB                   0
LB                 0.30 Hz
GB                    0
PC                 1.00
```

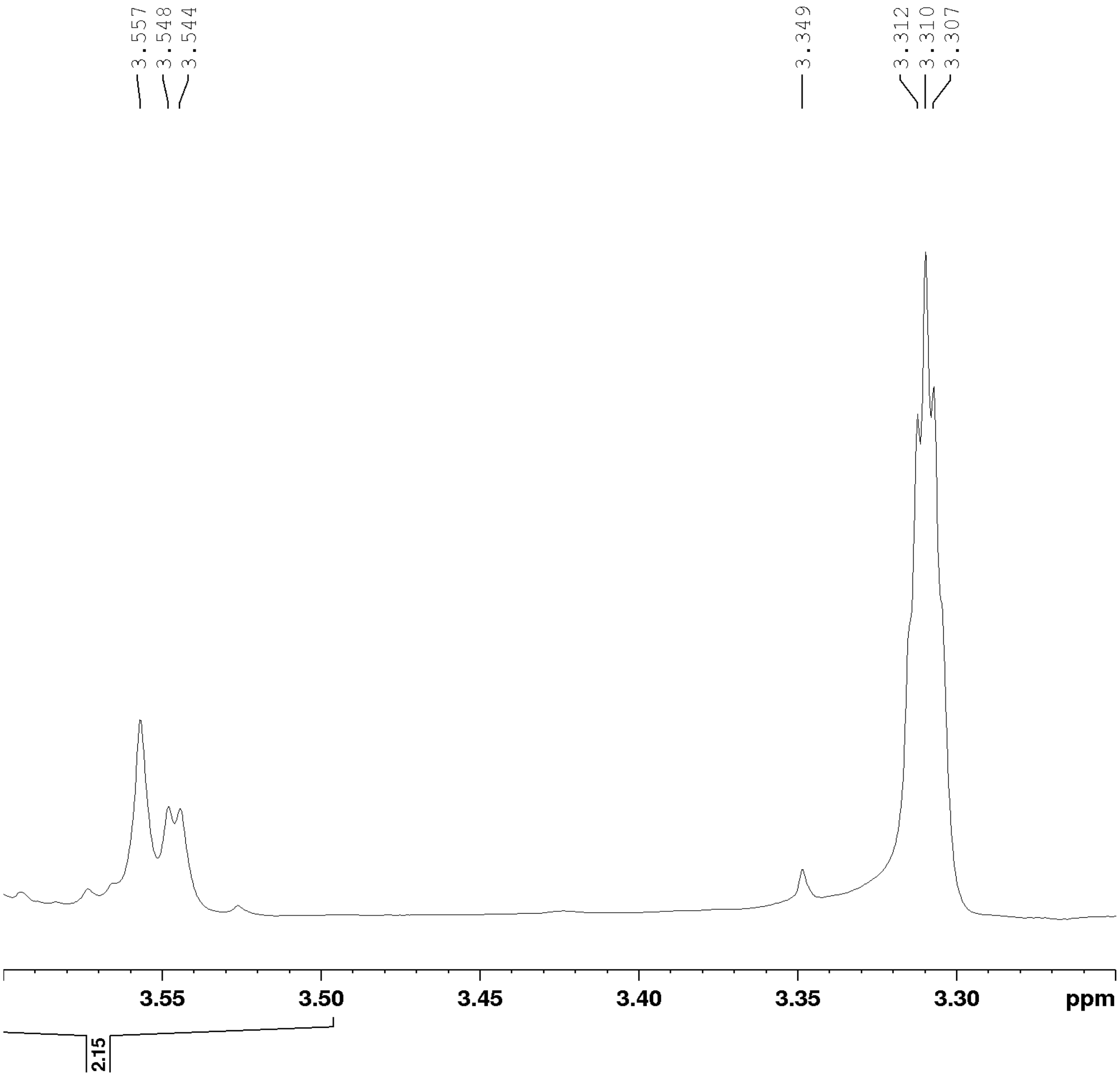


```
Current Data Parameters
NAME     168CLC-57.3-rfc80%-rp10-1H-CD3OD-2.9 mg
EXPNO                  1
PROCNO                 1

F2 - Acquisition Parameters
Date_           20180717
Time               11.43 h
INSTRUM            spect
PROBHD   Z114607_0270 (
PULPROG             zg30
TD                 65536
SOLVENT             MeOD
NS                    64
DS                     2
SWH            12019.230 Hz
FIDRES          0.366798 Hz
AQ             2.7262976 sec
RG                109.62
DW                41.600 usec
DE                  6.50 usec
TE                 296.5 K
D1            1.00000000 sec
TD0                    1
SFO1         600.3147069 MHz
NUC1                  1H
P1                 10.00 usec
PLW1         24.25499916 W

F2 - Processing parameters
SI                 65536
SF           600.3110118 MHz
WDW                   EM
SSB                    0
LB                  0.30 Hz
GB                     0
PC                  1.00
```

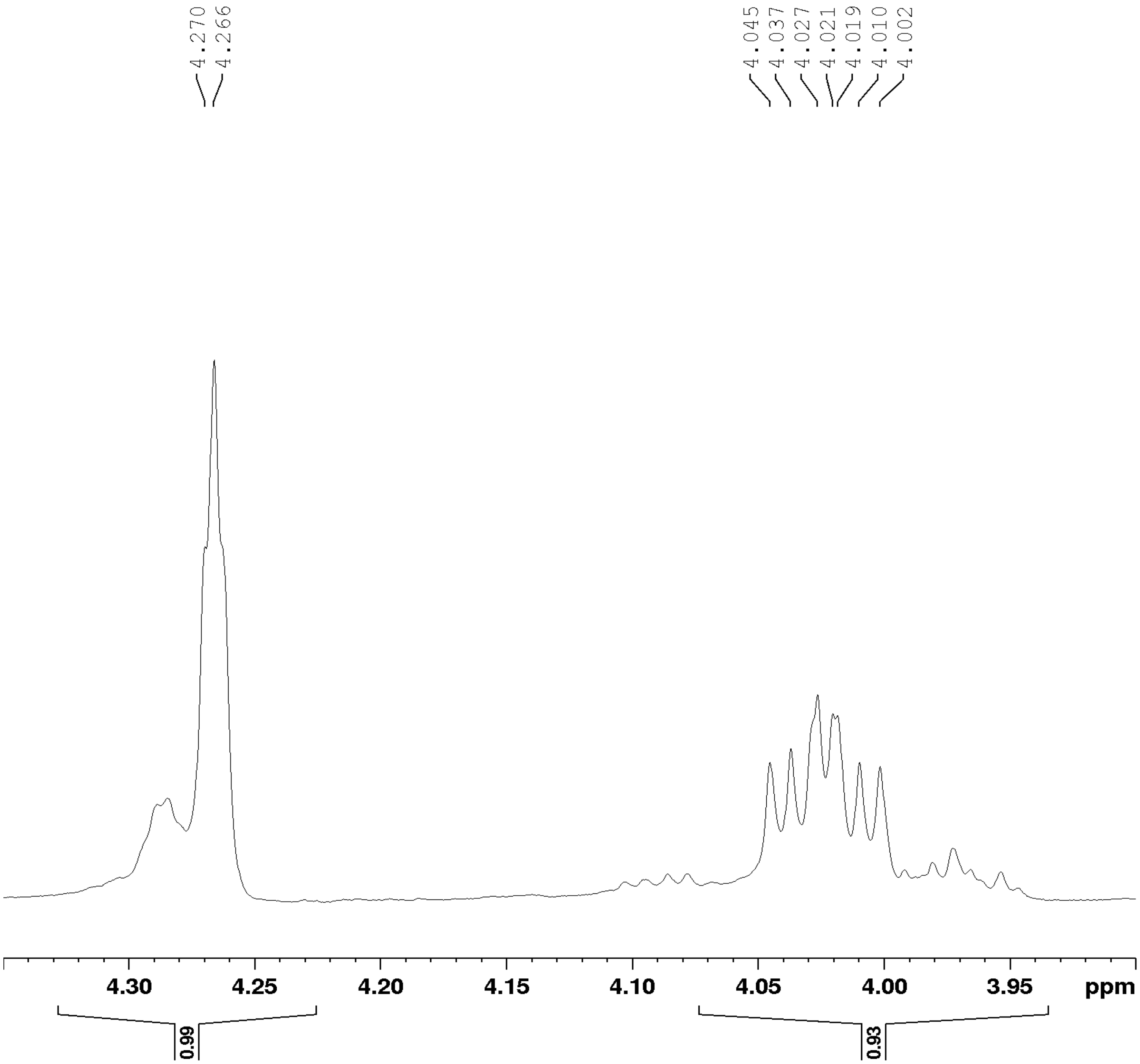


```
Current Data Parameters
NAME     168CLC-57.3-rfc80%-rp10-1H-CD3OD-2.9 mg
EXPNO              1
PROCNO             1

F2 - Acquisition Parameters
Date_          20180717
Time              11.43 h
INSTRUM           spect
PROBHD    Z114607_0270 (
PULPROG            zg30
TD                65536
SOLVENT            MeOD
NS                   64
DS                    2
SWH           12019.230 Hz
FIDRES         0.366798 Hz
AQ            2.7262976 sec
RG               109.62
DW               41.600 usec
DE                 6.50 usec
TE                296.5 K
D1           1.00000000 sec
TD0                   1
SFO1        600.3147069 MHz
NUC1                 1H
P1                10.00 usec
PLW1        24.25499916 W

F2 - Processing parameters
SI                65536
SF          600.3110118 MHz
WDW                  EM
SSB                   0
LB                 0.30 Hz
GB                    0
PC                 1.00
```

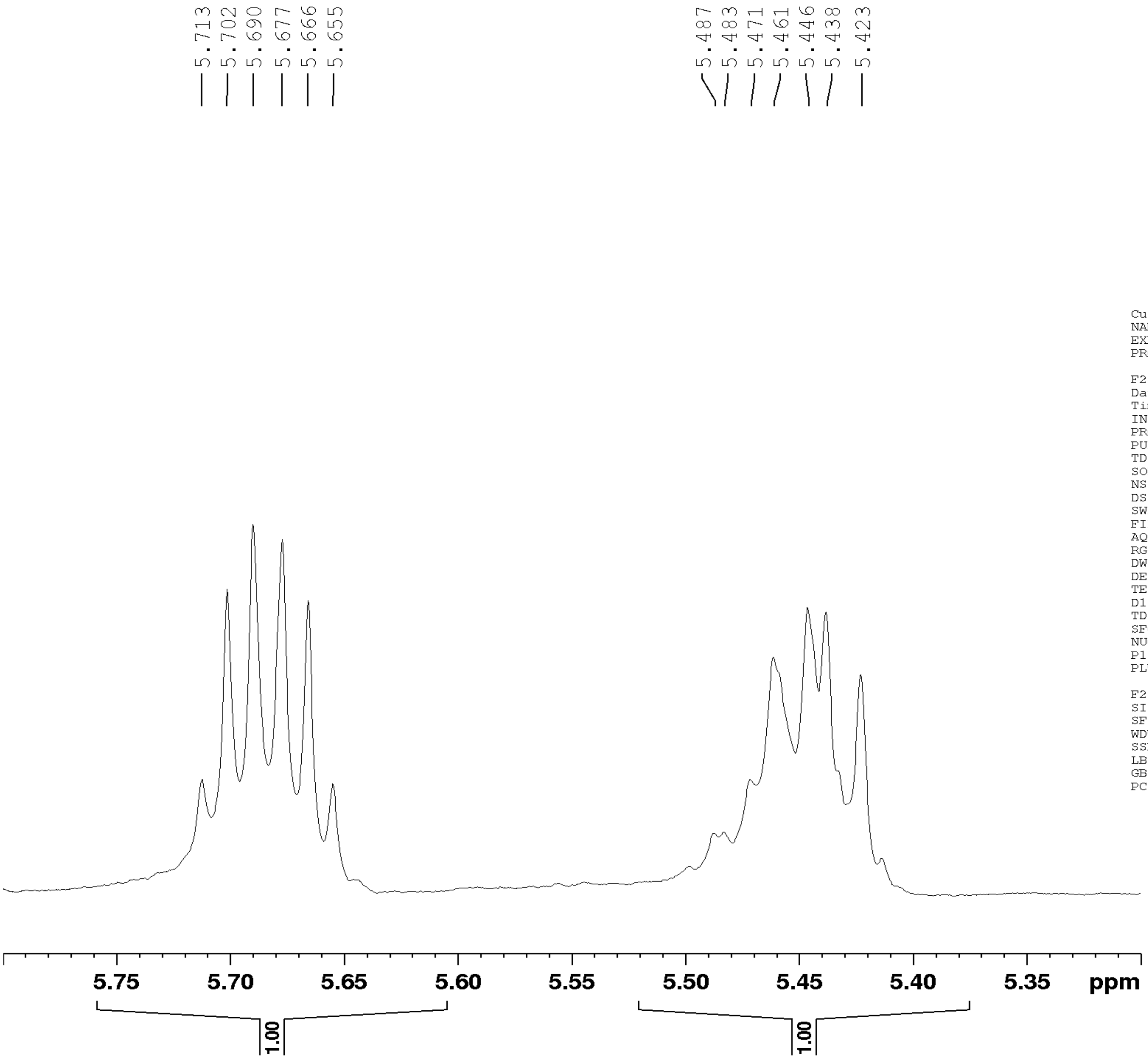

Current Data Parameters
NAME 168CLC-57.3-rfc80%-rp10-1H-CD3OD-2.9 mg
EXPNO 1
PROCNO 1

F2 - Acquisition Parameters
Date_ 20180717
Time 11.43 h
INSTRUM spect
PROBHD Z114607_0270 (
PULPROG zg30
TD 65536
SOLVENT MeOD
NS 64
DS 2
SWH 12019.230 Hz
FIDRES 0.366798 Hz
AQ 2.7262976 sec
RG 109.62
DW 41.600 usec
DE 6.50 usec
TE 296.5 K
D1 1.00000000 sec
TD0 1
SFO1 600.3147069 MHz
NUC1 1H
P1 10.00 usec
PLW1 24.25499916 W

F2 - Processing parameters
SI 65536
SF 600.3110118 MHz
WDW EM
SSB 0
LB 0.30 Hz
GB 0
PC 1.00

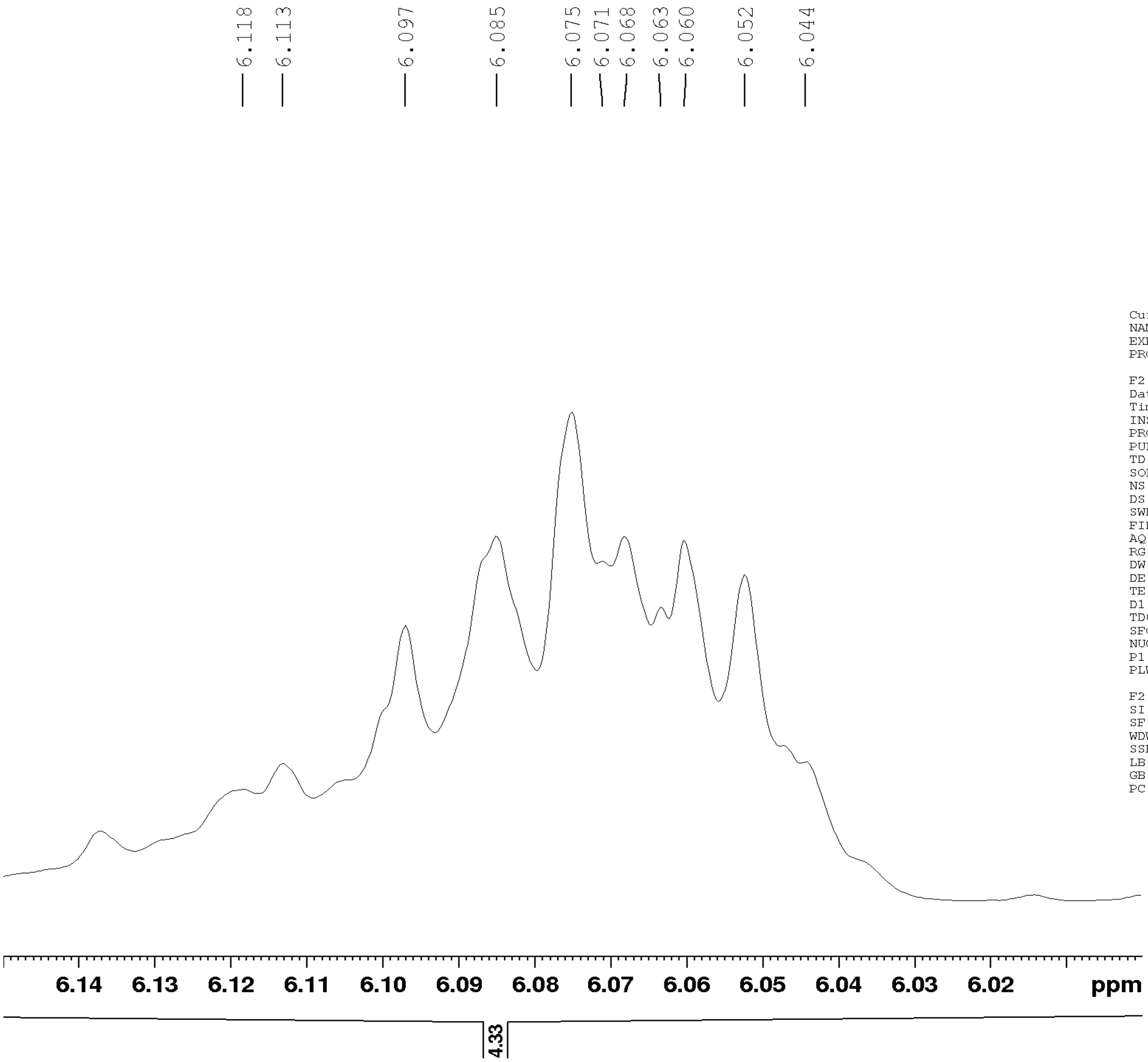


```
Current Data Parameters
NAME     168CLC-57.3-rfc80%-rp10-1H-CD3OD-2.9 mg
EXPNO              1
PROCNO             1

F2 - Acquisition Parameters
Date_          20180717
Time              11.43 h
INSTRUM           spect
PROBHD   Z114607_0270 (
PULPROG            zg30
TD                65536
SOLVENT            MeOD
NS                   64
DS                    2
SWH           12019.230 Hz
FIDRES         0.366798 Hz
AQ            2.7262976 sec
RG               109.62
DW               41.600 usec
DE                 6.50 usec
TE                296.5 K
D1           1.00000000 sec
TD0                   1
SFO1        600.3147069 MHz
NUC1                 1H
P1                10.00 usec
PLW1        24.25499916 W

F2 - Processing parameters
SI                65536
SF          600.3110118 MHz
WDW                  EM
SSB                   0
LB                 0.30 Hz
GB                    0
PC                 1.00
```

Figure S43. ^{13}C NMR spectrum of 2-O-acetyldendrodochol B (10)

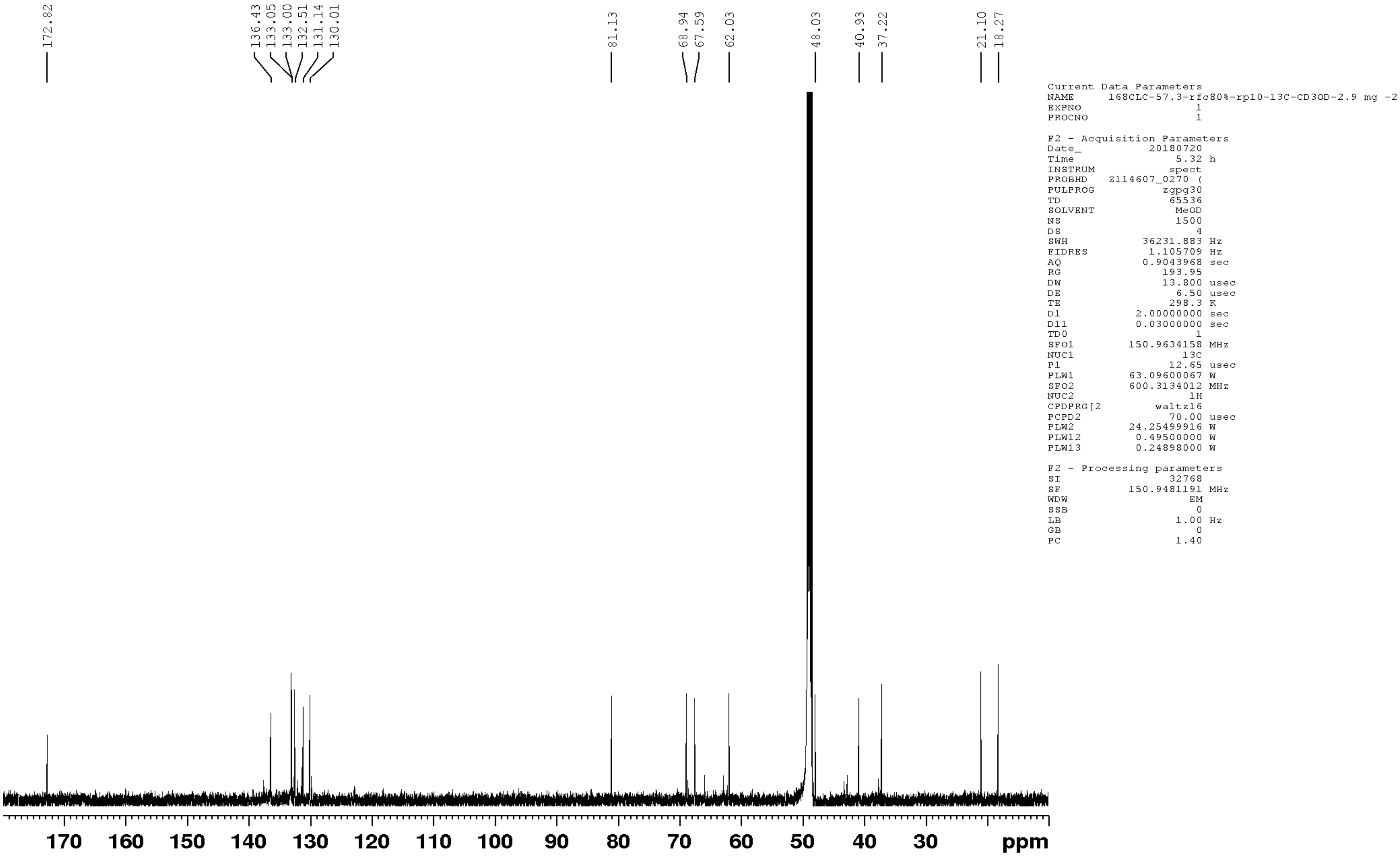


```
Current Data Parameters
NAME      168CLC-57.3-rfc80%-rp10-13C-CD3OD-2.9 mg -2
EXPNO                 1
PROCNO                1

F2 - Acquisition Parameters
Date_           20180720
Time                5.32 h
INSTRUM            spect
PROBHD   Z114607_0270 (
PULPROG           zgpg30
TD                 65536
SOLVENT             MeOD
NS                  1500
DS                     4
SWH            36231.883 Hz
FIDRES          1.105709 Hz
AQ             0.9043968 sec
RG                193.95
DW                13.800 usec
DE                  6.50 usec
TE                 298.3 K
D1            2.00000000 sec
D11           0.03000000 sec
TD0                    1
SFO1         150.9634158 MHz
NUC1                 13C
P1                 12.65 usec
PLW1         63.09600067 W
SFO2         600.3134012 MHz
NUC2                  1H
CPDPRG[2         waltz16
PCPD2              70.00 usec
PLW2         24.25499916 W
PLW12         0.49500000 W
PLW13         0.24898000 W

F2 - Processing parameters
SI                 32768
SF           150.9481191 MHz
WDW                   EM
SSB                    0
LB                  1.00 Hz
GB                     0
PC                  1.40
```

Figure S44. HMBC spectrum of 2-O-acetyldendrodochol B (10)

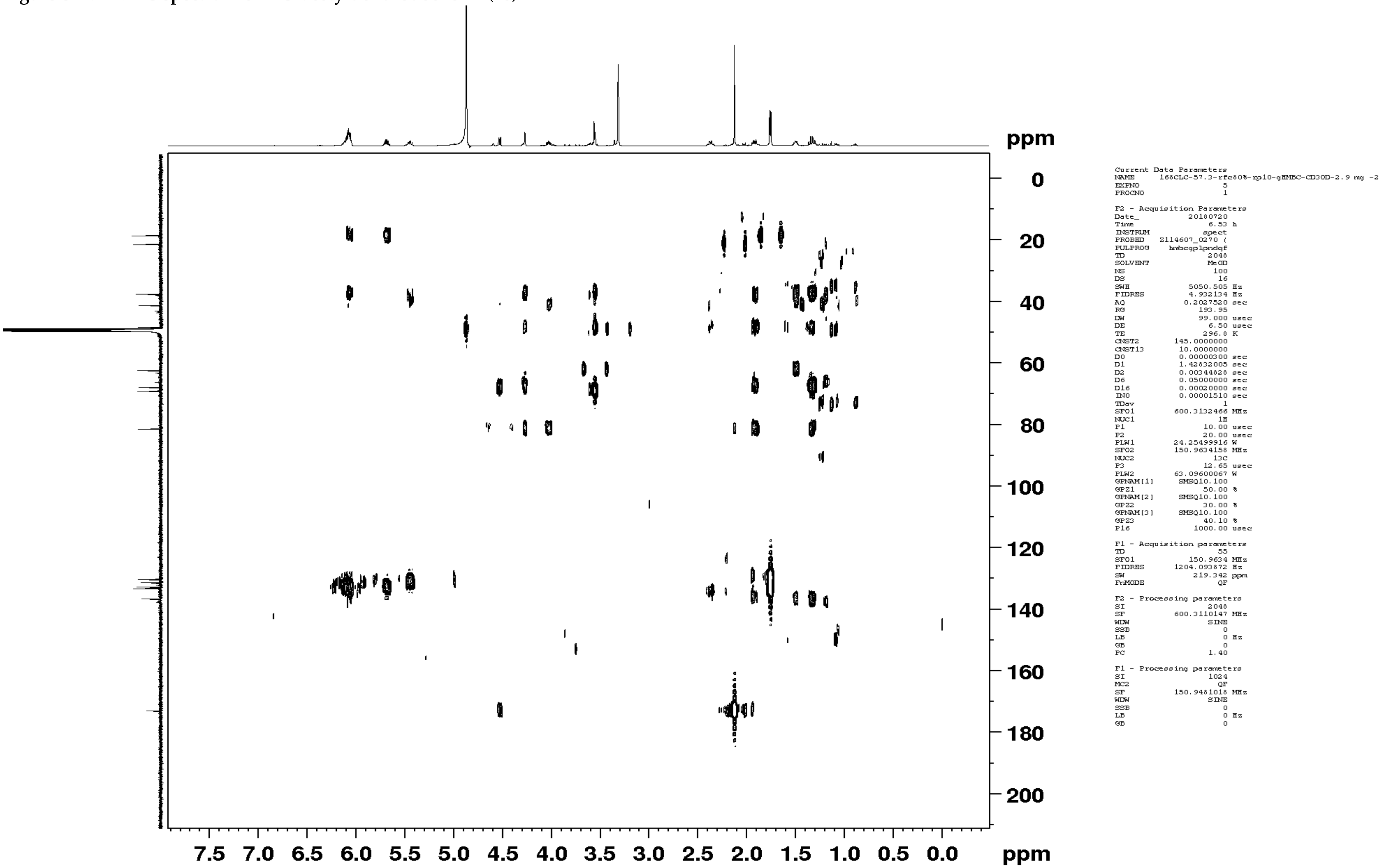

Figure S45. HSQC spectrum of 2-O-acetyldendrodochol B (10)

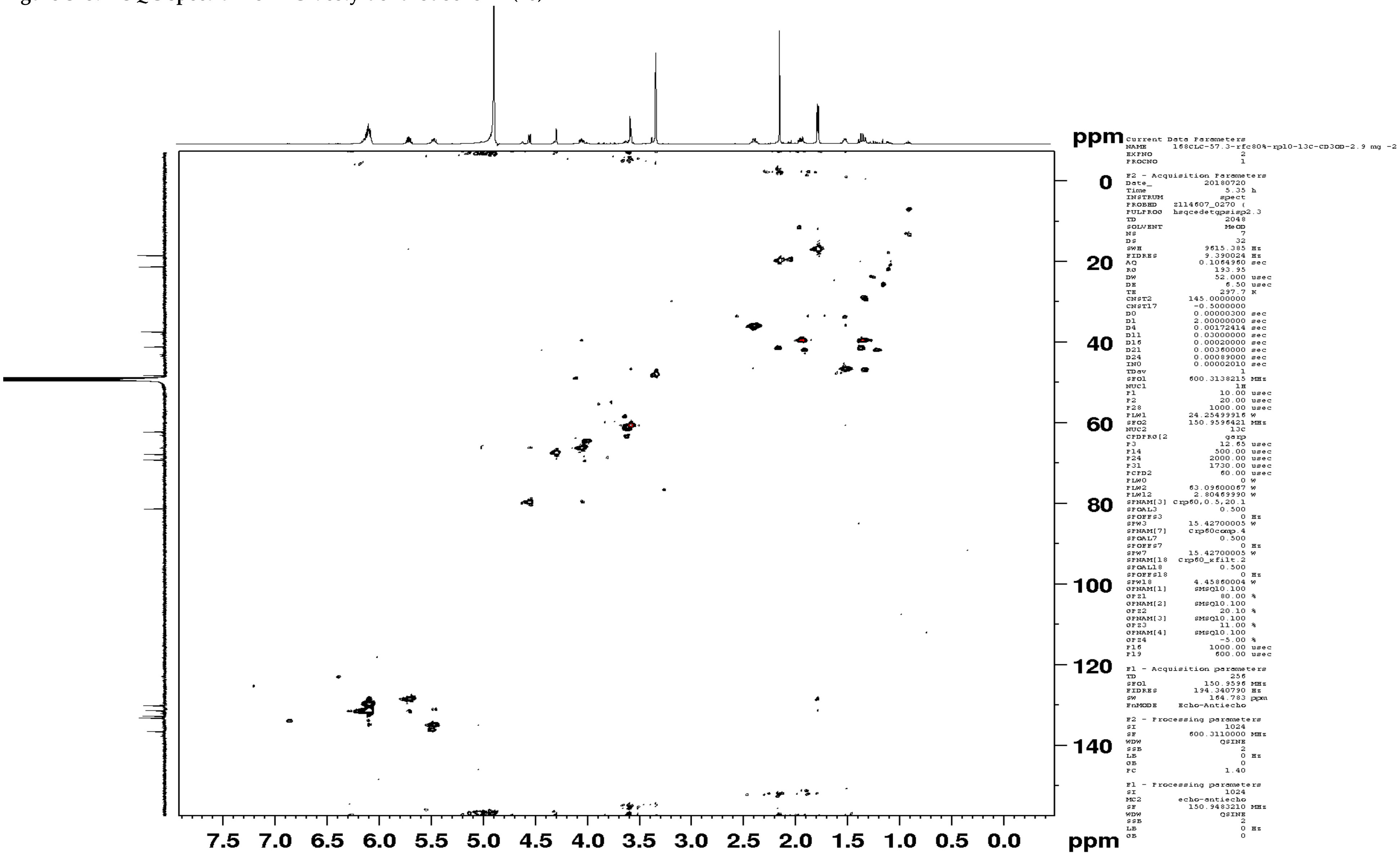

Figure S46. HR (+)ESI MS spectrum of lopouzanone A (1)

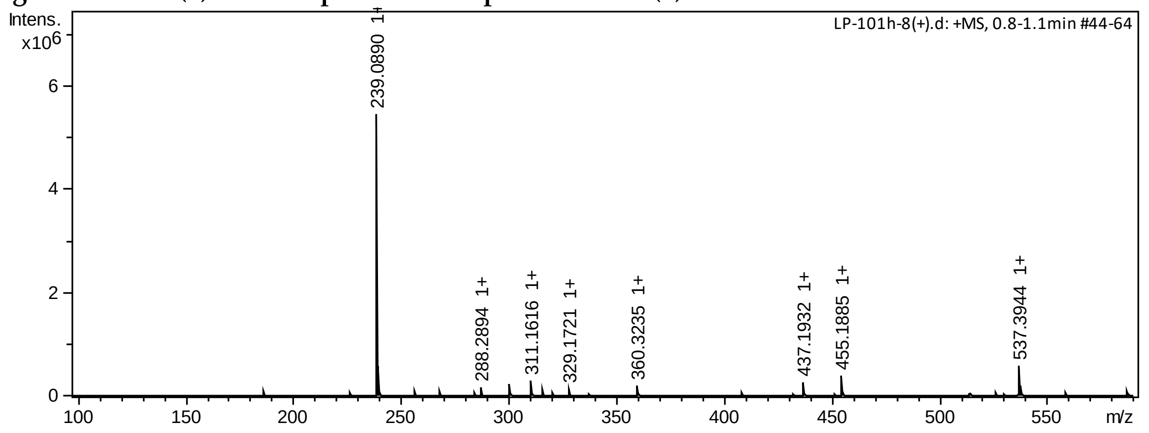

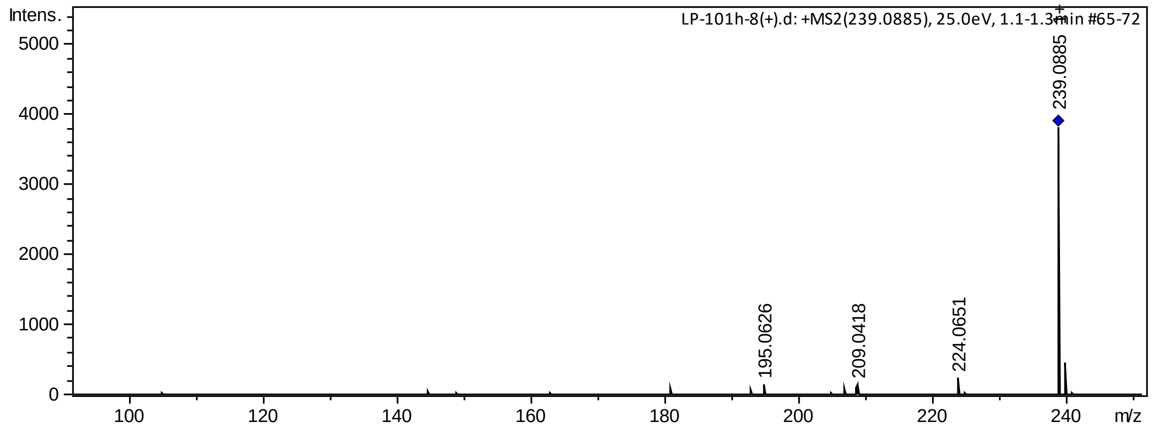

Figure S47. HR (+)ESI MS spectrum of lopouzanone B (2)

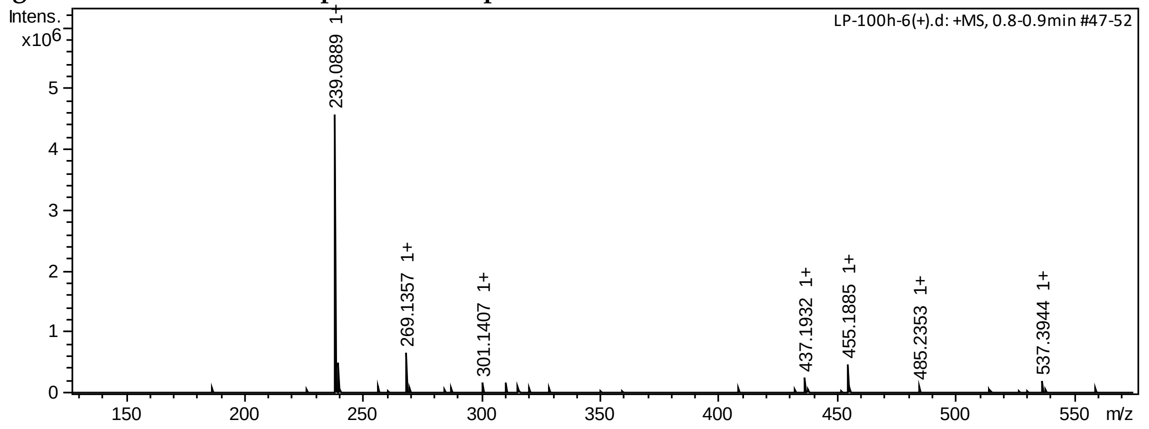

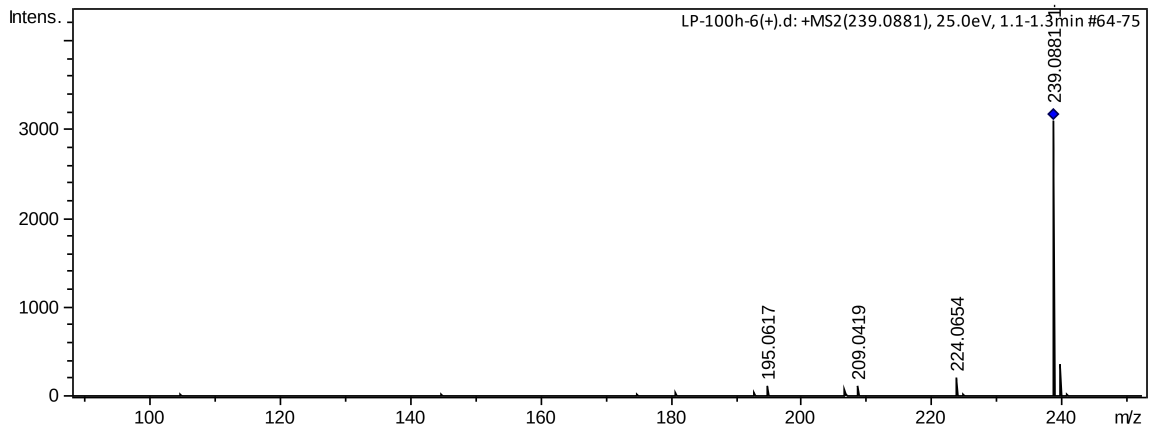

Figure S48. HR (+)ESI MS spectrum of dendrodochol B (8)

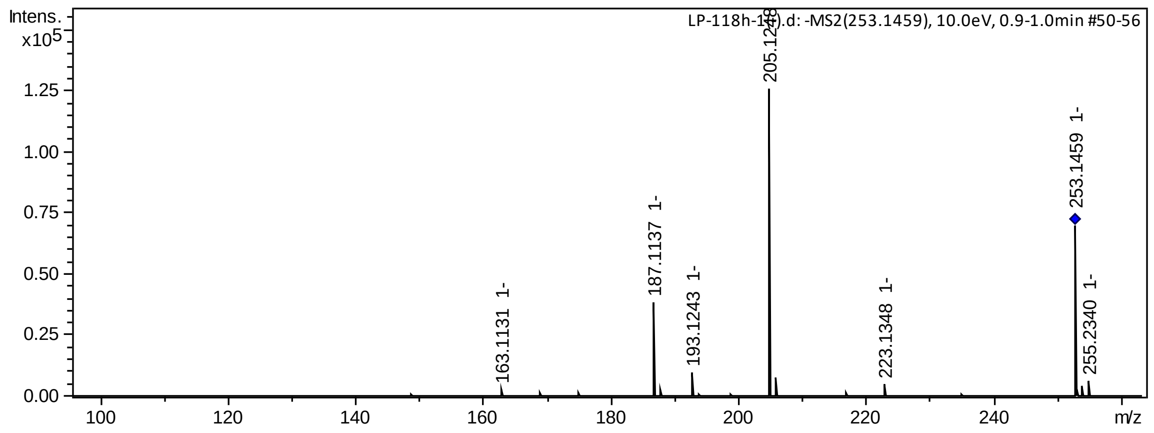

Figure S49. HR (+)ESI MS spectrum of 1-O-acetyldendrodochol B (9)

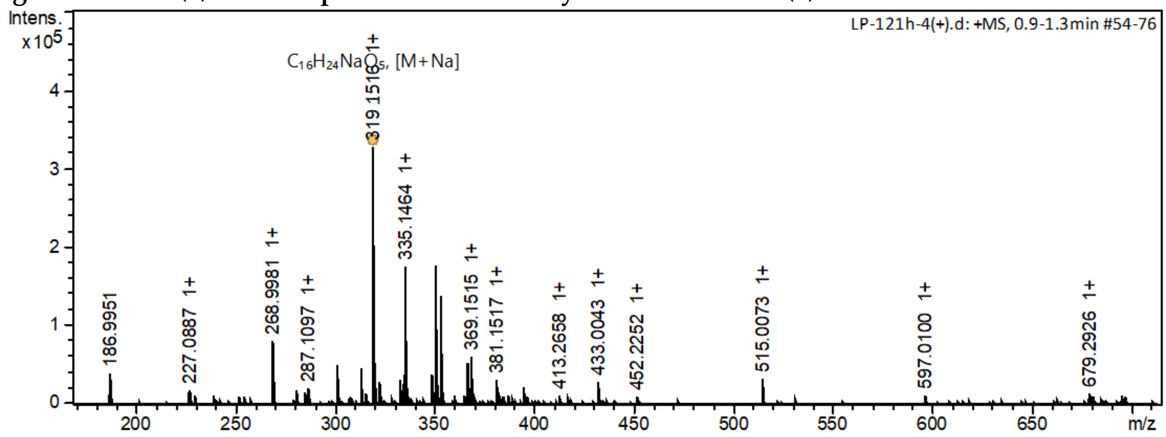

Figure S50. HR (+)ESI MS spectrum of 2-O-acetyldendrodochol B (10)

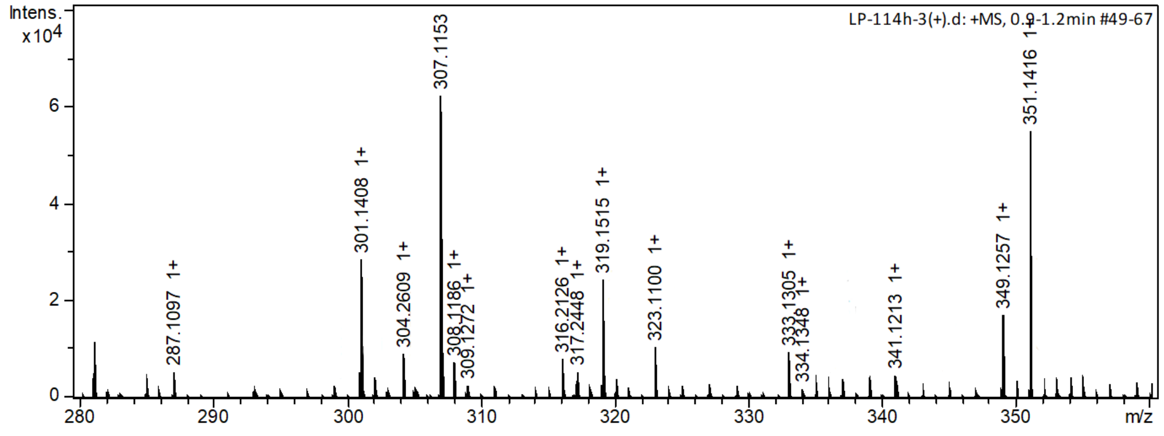

Supplement: Supplementary file 1 [file molecules-27-07650-s001.zip › molecules-1980047-supplementary.pdf]
